# *In silico* prediction of neuropeptides in Hymenoptera parasitoid wasps

**DOI:** 10.1371/journal.pone.0193561

**Published:** 2018-02-28

**Authors:** Juhua Chang, Jianhua Zhao, Xiaoli Tian

**Affiliations:** 1 College of Life Science, Yangtze University, Jingzhou, China; 2 Pesticide Research Institute, Yangtze University, Jingzhou, China; 3 Vegetable Technology Center of Xiyang County, Xiyang, China; Biocenter, Universität Würzburg, GERMANY

## Abstract

Parasitoid wasps of the order Hymenoptera, the most diverse groups of animals, are important natural enemies of arthropod hosts in natural ecosystems and can be used in biological control. To date, only one neuropeptidome of a parasitoid wasp, *Nasonia vitripennis*, has been identified. This study aimed to identify more neuropeptides of parasitoid wasps, by using a well-established workflow that was previously adopted for predicting insect neuropeptide sequences. Based on publicly accessible databases, totally 517 neuropeptide precursors from 24 parasitoid wasp species were identified; these included five neuropeptides (CNMamide, FMRFamide-like, ITG-like, ion transport peptide-like and orcokinin B) that were identified for the first time in parasitoid wasps, to our knowledge. Next, these neuropeptides from parasitoid wasps were compared with those from other insect species. Phylogenetic analysis suggested the divergence of AST-CCC within Hymenoptera. Further, the encoding patterns of CAPA/PK family genes were found to be different between Hymenoptera species and other insect species. Some neuropeptides that were not found in some parasitoid superfamilies (*e*.*g*., sulfakinin), or considerably divergent between different parasitoid superfamilies (*e*.*g*., sNPF) might be related to distinct physiological processes in the parasitoid life. Information of neuropeptide sequences in parasitoid wasps can be useful for better understanding the phylogenetic relationships of Hymenoptera and further elucidating the physiological functions of neuropeptide signaling systems in parasitoid wasps.

## Introduction

Parasitoid wasps (Order: Hymenoptera) are one of the most species-rich groups of animals, potentially accounting for more than 20% of the insects found globally [1]. Studies on insect parasitoids are important to characterize their biodiversity, understand their evolution and, in some cases, apply their parasitic abilities for practical purposes such as biological control of agricultural pests.

Neuropeptides represent the most diverse group of messenger molecules with regard to numbers and primary structures. They are important regulators of many physiological processes in insects, such as development, reproduction, feeding, locomotion, courtship, olfaction, and circadian rhythm [2–4]. Recent advances in genomic analysis have led to the discovery of neuropeptides and their receptors in several insects, *e*.*g*., *Drosophila melanogaster* [5], *Anopheles gambiae* [6], *Apis mellifera* [7], *Bombyx mori* [8], *Tribolium castaneum* [9], *Acyrthosiphon pisum* [10], *Rhodnius prolixus* [11], *Zootermopsis nevadensis* [12], *Locusta migratoria* [12], *Nilaparvata lugens* [13], *Mastotermes darwiniensis* [14], *Chilo suppressalis* [15] and *Lygus Hesperus* [16]. Peptidomic analysis using mass spectrometric analyses are also available for boosting the identification of neuropeptides in insects, e.g., *Tribolium castaneum* [9], *Apis mellifera* [17], *Rhodnius prolixus* [18], *Pseudatomoscelis seriatus* [19], *Drosophila suzukii* [20], *Camponotus floridanus* [21] and *Oligotoma saundersii* [22]. However, thus far, only one neuropeptide set of a parasitoid wasp, *Nasonia vitripennis*, has been identified [23].

In the present study, the publicly accessible sequence data were mined for identifying putative precursor sequences of neuropeptides in parasitoid wasps, and mature bioactive peptide sequences were predicted using a well-established *in silico* workflow (*e*.*g*. Huybrechts et al. [10], Veenstra [12], Christie [14], Xu et al. [15], Christie et al. [16], Christie [24]). Totally more than 500 neuropeptide precursors were found from 24 parasitoid wasp species belonging to six superfamilies: Chalcidoidea, Ichneumonoidea, Cynipoidea, Chrysidoidea, Orussoidea and Platygastroidea. All of these superfamilies except Orussoidea belong to Suborder Apocrita. All parasitoid taxa from Orussoidea and most of parasitoid taxa from Chrysidoidea are ectoparasitoids or cleptoparasitoids; all parasitoid taxa from Cynipoidea and Platygastroidea are endoparasitoids; and both ectoparasitoids and endoparasitoids are included from two of the largest superfamilies (Chalcidoidea and Ichneumonoidea), as reviewed by Whitfield [25]. Mining of neuropeptides of parasitoid wasps might be helpful for further understanding their physiological roles.

## Materials and methods

### Database searches

Database searches were conducted on or before Jan 14, 2017, by using the methods modified from a well-established protocol (*e*.*g*. Huybrechts et al. [10], Veenstra [12], Christie [14], Xu et al. [15], Christie et al. [16], Christie [24]). Known neuropeptide precursor sequences of two Hymenoptera species, *A*. *mellifera* [7] and *N*. *vitripennis* [23], and of other insect species [5, 8–13] were used as reference. The putative neuropeptide precursor sequences of different representative parasitoid wasp species were detected using the tblastn program for online searchers in the NCBI databases (http://blast.ncbi.nlm.nih.gov/Blast.cgi) [26]. The database was set to “Non-redundant protein sequences” or “Transcriptome Shotgun Assembly” (restricted from “Ichneumonoidea taxid: 7401, Chalcidoidea taxid: 7422, Chrysidoidea taxid:40304, Orussoidea taxid: 222831, Cynipoidea taxid: 40307 or Platygastroidea taxid: 81084, Stephanoidea taxid: 85766, Trigonalyoidea taxid: 27487, Megalyroidea taxid: 44356, Evanioidea taxid: 27483, Ceraphronoidea taxid: 44357 and Proctotrupoidea taxid: 40308”; these superfamilies were chosen based on an article previously reported [25], except Vespoidea and Apoidea containing many species with pollen and nectar feeding or predation habits). The algorithm parameters were set to default. All hits by a given search were completely translated using ExPASy (http://web.expasy.org/translate/), and then checked manually for homology to the target query. All neuropeptide precursor sequences used in this study are listed in S1 Table.

### Peptide prediction

The workflow mentioned above was used for predicting the structures of mature peptides. In particular, each deduced precursor protein was assessed for the presence of a signal peptide by using the online program SignalP 4.1 (http://www.cbs.dtu.dk/services/SignalP/) [27], and prohormone cleavage sites were identified based on similarities with known neuropeptides by using the information in Veenstra [28].

### Phylogenetic analysis and sequence alignment

ClustalX software [29] was used to perform multiple sequence alignments, by using the slow-accurate mode with a gap-opening penalty of 10 and gap-extension penalty of 0.1, and applying the default Gonnet protein weight matrix. Alignments were visualized using Bioedit v7.0.5.3. Phylogenetic trees of precursor sequences were constructed in MEGA v6.06 [30] by using the neighbor-joining method and bootstrap analysis with 1000 replicates. Sequence logos of manually aligned homologous neuropeptide sequences were generated using the online tool WebLogo (http://weblogo.berkeley.edu/logo.cgi) [31].

## Results and discussion

### The *in silico* mining of neuropeptides of parasitoid wasp species

Mining from the publicly accessible databases led to the *in silico* prediction of a total of 517 precursors from 24 parasitoid wasp species belonging to six superfamilies: Chalcidoidea, Ichneumonoidea, Cynipoidea, Chrysidoidea, Orussoidea and Platygastroidea (S1 Table). All the neuropeptide precursors with the predicted putative mature peptide structures are shown in S1 Fig. The putative mature peptides in three representative species (*N*. *vitripennis*, *Fopius arisanus*, and *Argochrysis armilla*) are shown in Table 1. Hauser et al. [23] found 30 precursor genes encoding neuropeptides of *N*. *vitripennis*. Allatotropin of *N*. *vitripennis* was previously identified by Veenstra et al. [32]. Additional neuropeptide genes of *N*. *vitripennis* were confirmed in this study, such as CNMamide (*CNMa*), FMRFamide-like (*FMRFa*), *ITG-like*, *orcokinin B* and an orthologous gene of ion transport peptide-like (*ITPL*) (S1 Table, S1 Fig). Some neuropeptides were not found in any parasitoid wasp species or other Hymenoptera species, such as allatostatin B (AST-B), glycoprotein hormone (alpha2 and beta5, GPA2 and GPB5), and proctolin.

**Table 1:** The putative mature peptides in *Nasonia vitripennis*, *Fopius arisanus* and *Argochrysis armilla*.

Peptide	*Nasonia vitripennis*	*Fopius arisanus*	*Argochrysis armilla*
ACP	pQVTFSKGWGPa	-	-
AKH	pQLNFSTGWa	pQLTFSTGWa	pQLNFSTGWa
AST-A	LPIYQFGLa, SQPFSFGLa, TRPYSFGLa, TGGFNFGLa, DKYLFGLa	FPDYLYSFGIa, NSPYSFGVa, NSPSHAYGFGIa	LPVYNFGIa, SRTYSFGLa, GRNYDFGLa, AGYVYRFGLa, PNEDVLHRYNFGIa
AST-CC	GQAKGRVYWRCYFNAVTCF	GPVNGSVYWRCYFNAVTCF	GQAKGRIYWRCYFNAVTCF
AST-CCC	NYWRQCAFNAVSCF	TYWKQCAFNAVSCF	SYWKQCAFNAVSCF
AT	GFQPEYISTAYGFa	GIRPGSLQTARDFa	GYKPEYISTAIGFa
AVLP	CLITNCPRGa	CLITNCPRGa	-
CCAP	PFCNAFTGCa	PFCNAFTGCa	PFCNAFTGCa
CCHa-1	SCLSYGHSCWGAHa	-	SCAQYGHSCWGGHa
CCHa-2	GCSAFGHSCYGGHa	-	GCSAFGHSCFGGHa
CNMa	TSYMALCHFKICNMa	TSYMGLCHFKICNMa	AKPASYMSLCYFKICNMa
CRZ	pQTFQYSRGWTNa	pQTFQYSRGWTTa	pQTFQYSHGWTNa
DH31	GLDLGLNRGFSGSQAAKHLMGLAAANYAGGPa	GGFGLDFGLNRGFSGAQAAKHLMGMAAANYAGGPa	GLDLGLSRGFSGSQAAKHLMGLAAANYAGGPa
DH44	IGSLSVVNSVDVLRERVLLELARRKAMENQQQLGENQYVFKSVa	ISSLSITNPMDVLRQRFILELARRRQMQQQEQAKANREILNDIa	-
ELN	-	GRQVRPLDCEKYVFHPHCRGTQA	RMVDCERYPFHSTCRGTMS
ETH	DEPPAFFLKIAKNIPRIa	DEVPAFFLKIAKNVPRIa	-
FMRFa	SSSSGGNLGSSFIRYa, SDVIIRYa	SQMGSSFIRFa, SDVIIRFa	…MGASFIRFa, FKSPDIVIRFa, ARSDLNFIRFa
ITG	ITGKNNRLY	ITQGQHNRNLLY	ITGQGNRLF
Kinin	-	NPSFSPWGa, PARVPFNSWGa, PFNSWGa	-
MS	pQDVDHVFLRFa	pQDVDHVFLRFa	pQDVDHVFLRFa
NTL	-	TIRQWTMQEPLYVEEPRWIPLDVKGDFNEEPGFNEEDPFILARa, NKHSDIMDILNEPFFISRa, GEIVEQLLKEQDPFYIARa	-
NPF	EPEPMARPTRPKVFESPEELRQYLDLVKEYYSLSGKARYa	-	EPEPMARPTRPKVITSPEELRRYLDSVKDFYTLNGKARYa
NPLP1	-	SLATLAKNGDLPVSIQERAQDGQEDDE, NLAALARESALPa, NIASMAREFGLPTa, NVGTLARDRQLPSa, PSYTLGRLFVIPVATa, NVGSLARDSALPPYa, GIASLAKNGDFPFP NVGTLARDWSLPQTRHa	SLATLAKNDDLPVTIRD, NIASLARDYGLPSa, NVASLARDFALPNa, NIGAMAREHLLPMGa, NVASLARVYMLPQNa, aNIAALARDYSLPS, NIASLARNADWPGLA
OK- A	NFDEIDRSGFSGFS, NFDEIDRSGFSGFN, NFDEIDRTGFSGFN, NFDEIDRSGVPGFA	NIDEIDRAGFDSFS, NFDEIDRAGWDSFVK	NIDEIDRTAFDSFF, NFDEIDRDGFDGLDDFT
OK- B	-	NLDHIGGGNLL	NLDQIGGGNLV, NLDHIGGGNLL
PDF	NSELINSLLSLPKNMNNAa	-	NSELINSLLGLPKNMNNAa
PVK	-	AAGILAQPRIa, ADGAAGLVQYPRVa	TAGLVPYPRIa, ASGLLHYPRVa, SSQGQLTLGGYTPRLa
PK	QETTFTPRLa, DQQAPPPMFPPRLa	HTTQFTPRLa, EFEDMTINHHQPPTPPPQFAPRLa, HLPFNPSPRLa	QSTSFTPRLa, SPSLYSPRLa
trypto-PK	QYDGRGSDMVEGPRVERMHPETSGGCVGAHCLTQNSEGPVGAMWFGPRLa	VDYDGDQPTSLDFNGLCNGGRCSETGDGIAGAMWFGPRLa	TTQEITSGMWFGPRLa
RYa	pQDNFYASGRFa, SEDRSAGNSLKDSSLFSSARFa, SEDRNTGNSLRDSSSFFPARYa, SEDRSTGNSLRDSSSFFPARFa, SEDRSTGNSLKDSSSFSPARYa, SEDRSSGNSLKESSFFSPGRYa, SEGHKNPKELPKFFEIKPRVDQFFIGSRYa	NNFYTQGRYa, PLFFPSRYa, SIPNSDETSGGGSKIVEISPRPDRFYLGSRYa	-
SIFa	AYRKPPFNGSIFa	AYRKPPFNGSIFa	AYKKPPFNGSIFa
sNPF	AAERSPSLRLRFa, SYPKYPRSPSLRLRFa	SQRSPSLRLRFa	SQRSPSLRLRFa
SK	-	-	QQFDDYGHMRFa, EKFDDYGHMRFa
TK	ASMRGFQGMRa, APMGFQGMRa, AMMGGFQGMRa, ALLGFHGMRa, PMMMGFHGMRa, SPYRFFGTRa, FVGVRa	APMGFQGMRa, ASMGFHGMRa, ALPMGFQGMRa, APMGFQGVRa, GSMGFVGMRa, SARGVSGVRa, IPRWEMRGTFIGVRa	APMGFQGMRa, AMMGFQGMRa, AMMMGFQGMRa, ARMGFQGMRa, AIMGFHGMRa
Bur-α	NV_13010	-	GAXO01016945.1
Bur-β	NV_03820	XM_011308217.1	GAXO01035212.1
EH	NV_08338	XM_011302509.1	-
ILP1	NV_03688	XM_011302058.1	GAXO01006850.1
ILP2	NV_30146	XM_011299587.1	GAXO01017855.1
ITP	XM_016917221.1	XM_011311875.1	-
ITPL	NV_07921	XM_011311883.1	GAXO01008554.1
NP	NV_03041	XM_011308237.1	-
PTTH	NV_30191	XM_011311235.1	-

Note: For a longer peptide sequence, the accession No. is given. AKH: adipokinetic hormone; ACP: AKH/corazonin-relate peptide; AST: allatostatin; AT: allatotropin; AVLP: arginine-vasopressin-like peptide; Bur: bursicon; CCAP: crustacean cardioactive peptide; CCHa: CCHamide; CNMa: CNMamide; CRZ: corazonin; DH: diuretic hormone; ELN: elevenin; EH: eclosion hormone; ETH: ecdysis triggering hormone; FMRFa: FMRFamide-like peptide; ILP: insulin-like peptide; ITG: ITG-ilke; ITP: ion transport peptide; ITPL: ITP-like peptide; MS: myosuppressin; NTL: natalisin; NP: neuroparsin; NPF: neuropeptide F; NPLP1: neuropeptide-like precursor 1; OK: orcokinin; PDF: pigment dispersing factor; PVK: periviscerokinin; pyrokinin: PK; PTTH: prothoracicotropic hormone; RYa: RYamide: SIFa: SIFamide; sNPF: short neuropeptide F; SK: sulfakinin; TK: tachykinin

**Fig 1:**
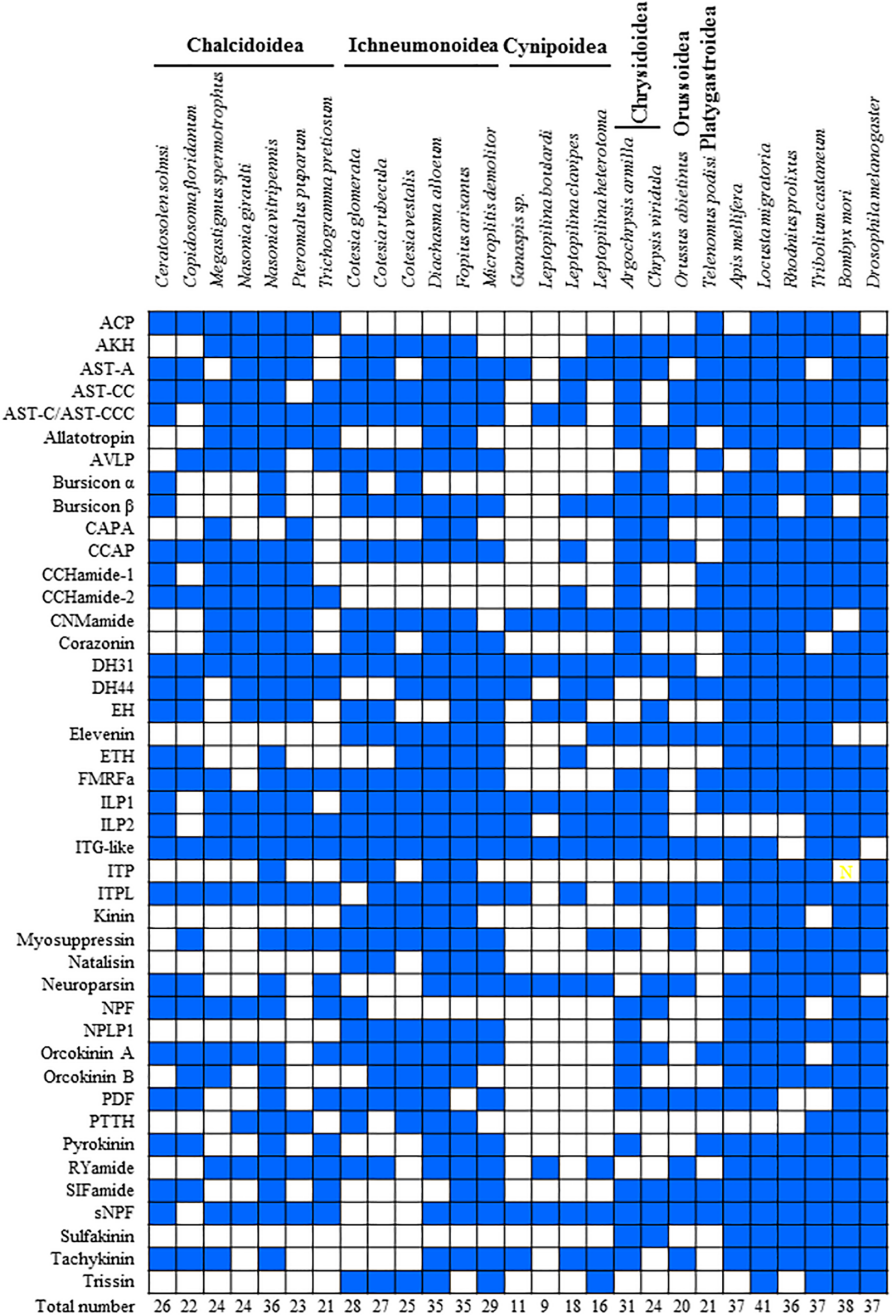
Overview of the presence of neuropeptide precursors of Hymenoptera parasitoid wasps and other insects. Blue, identified neuropeptide precursors; White, not found. AKH: adipokinetic hormone; ACP: AKH/corazonin-relate peptide; AST: allatostatin; AVLP: arginine-vasopressin-like peptide; CAPA: cardioacceleratory peptide 2b; CCAP: crustacean cardioactive peptide; DH: diuretic hormone; EH: eclosion hormone; ETH: ecdysis triggering hormone; FMRFa: FMRFamide-like peptide; ILP: insulin-like peptide; ITP: ion transport peptide; ITPL: ITP-like peptide; NPF: neuropeptide F; NPLP1: neuropeptide-like precursor 1; PDF: pigment dispersing factor; PTTH: prothoracicotropic hormone; sNPF: short neuropeptide F. The data of other insects are mainly referred from *D*. *melanogaster* [5], *An*. *gambiae* [6], *A*. *mellifera* [7], *B*. *mori* [8], *T*. *castaneum* [9], *Ac*. *pisum* [10], *R*. *prolixus* [11], *Z*. *nevadensis* [12], *L*. *migratoria* [12], *N*. *lugens* [13], *C*. *suppressalis* [15].

**Fig 2:**
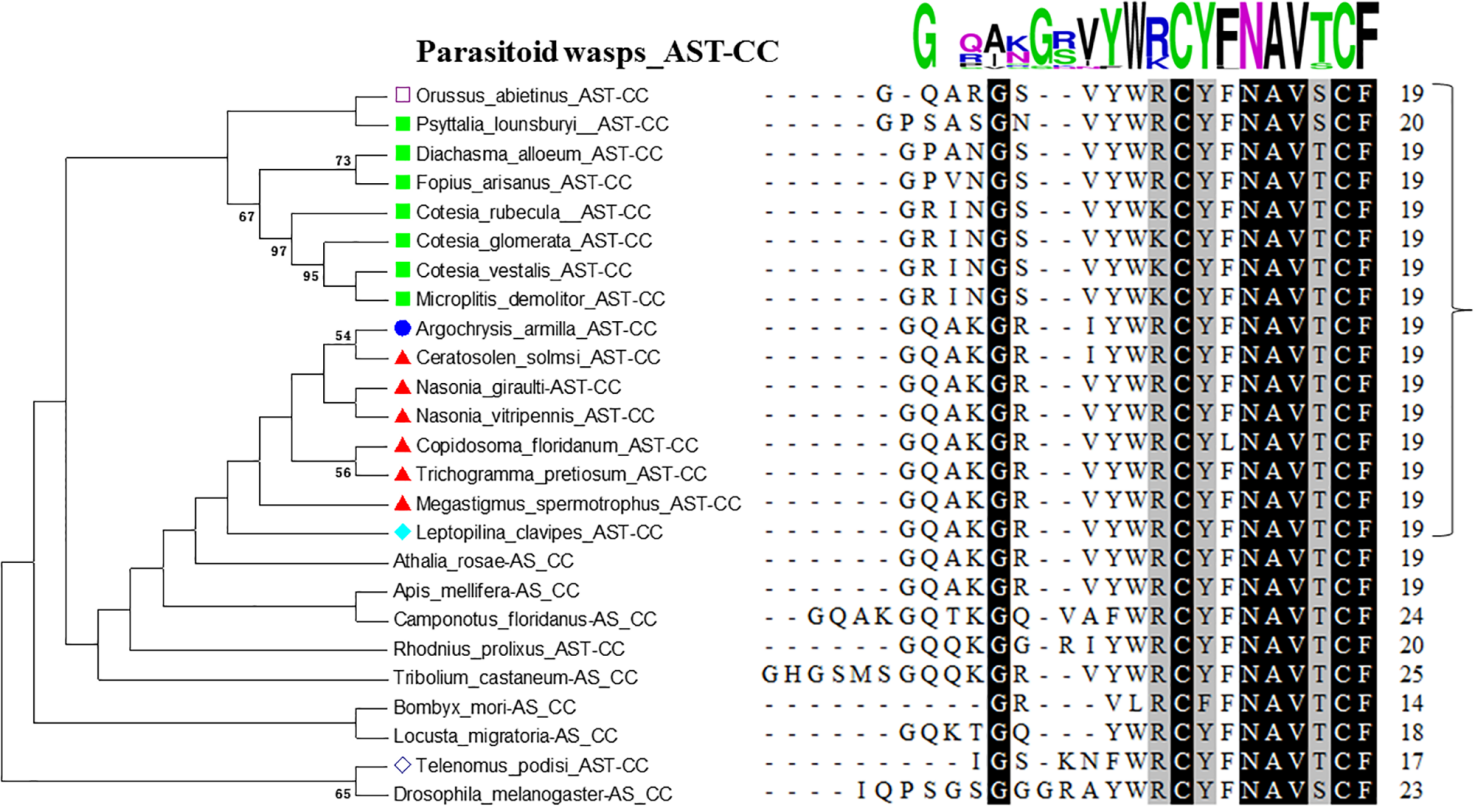
Phylogenetic tree of AST-CC precursors and alignment analysis of AST-CC sequences in parasitoid wasps and other insect species. Chrysidoidea sequences in phylogeny trees are indicated with blue circles; Ichneumonoidea sequences are indicated with light green squares; Chalcidoidea sequences are indicated with red triangles; Cynipoidea sequences are indicated with light blue rhombuses; Orussoidea with empty squares. Numbers above branches indicate phylogenies from amino acid sequences and only values above 50% are shown. Identities in alignments are highlighted in dark (100%) and in grey (80%~100%).

**Fig 3:**
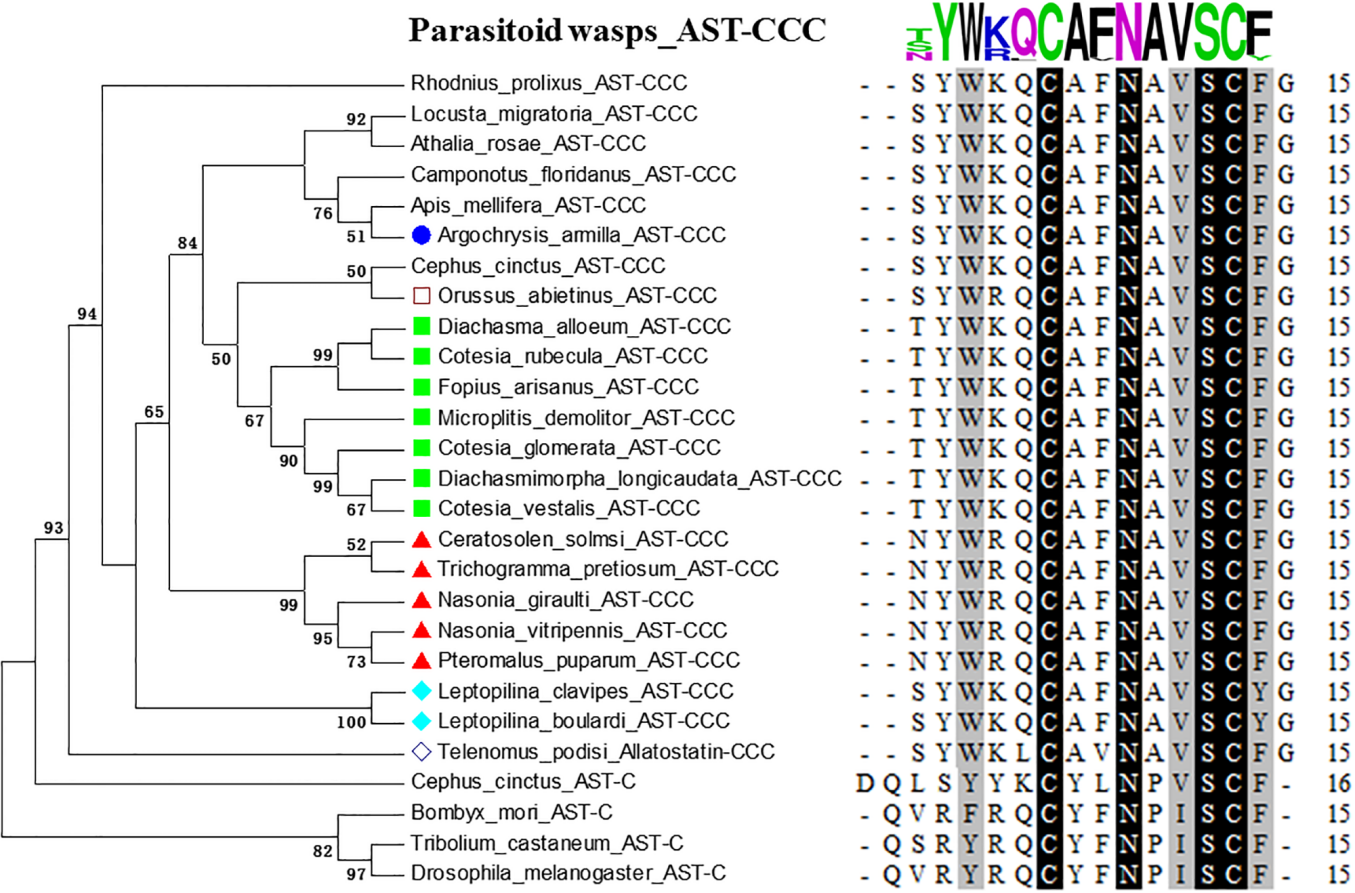
Phylogenetic tree of AST-C/AST-CCC precursors and alignment analysis of AST-C/AST-CCC sequences in parasitoid wasps and other insect species. Chrysidoidea sequences in phylogeny trees are indicated with blue circles; Ichneumonoidea sequences are indicated with light green squares; Chalcidoidea sequences are indicated with red triangles; Cynipoidea sequences are indicated with light blue rhombuses; Orussoidea with empty squares; Platygastroidea with empty rhombuses. Numbers above branches indicate phylogenies from amino acid sequences and only values above 50% are shown. Identities in alignments are highlighted in dark (100%) and in grey (80%~100%).

**Fig 4:**
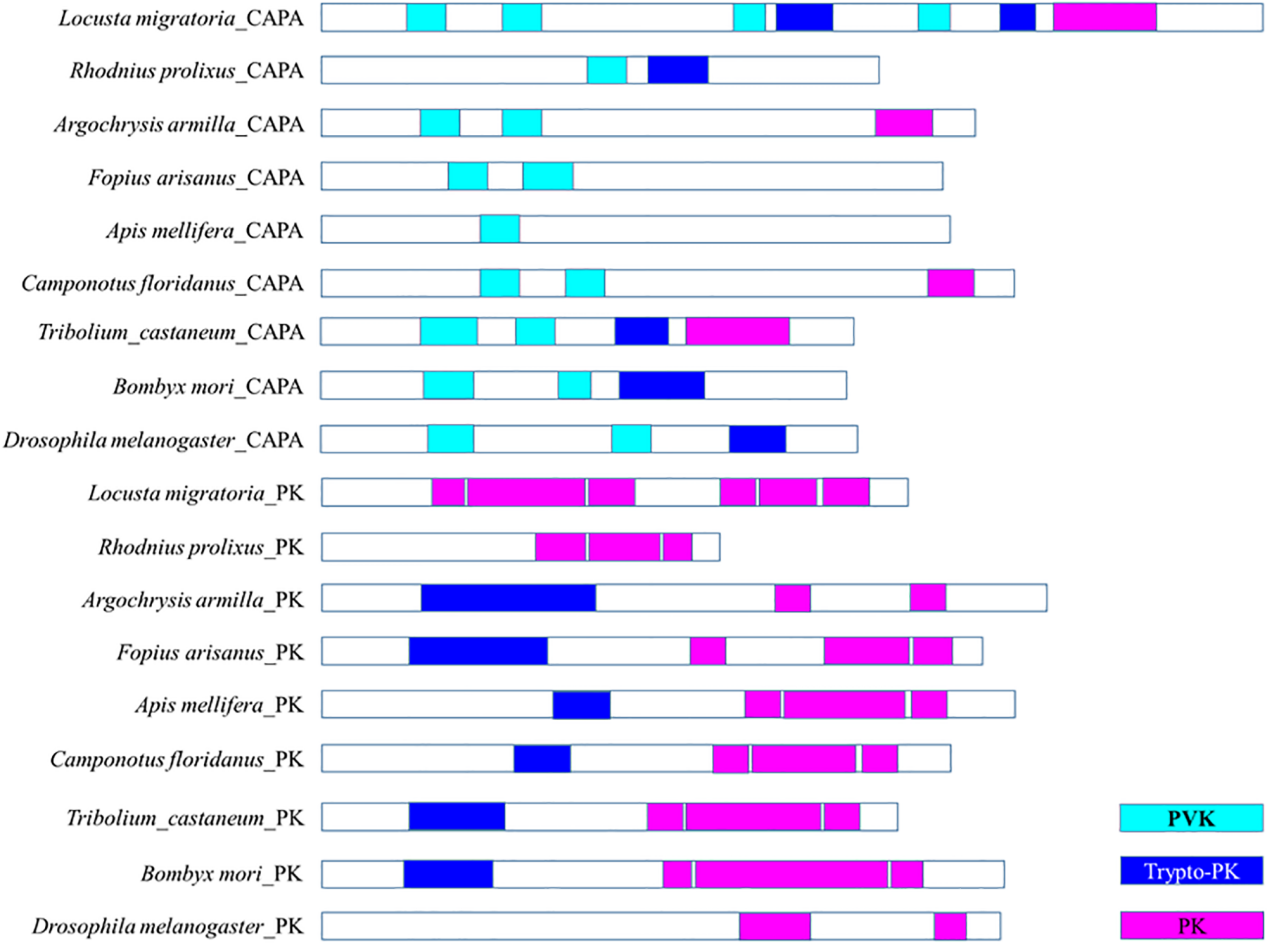
Schematic diagrams for CAPA/PK genes in parasitoid wasps and other insect species. Putative bioactive mature peptides are shown as color coded boxes for each peptide family (PVKs, PKs and trypto-PKs).

**Fig 5:**
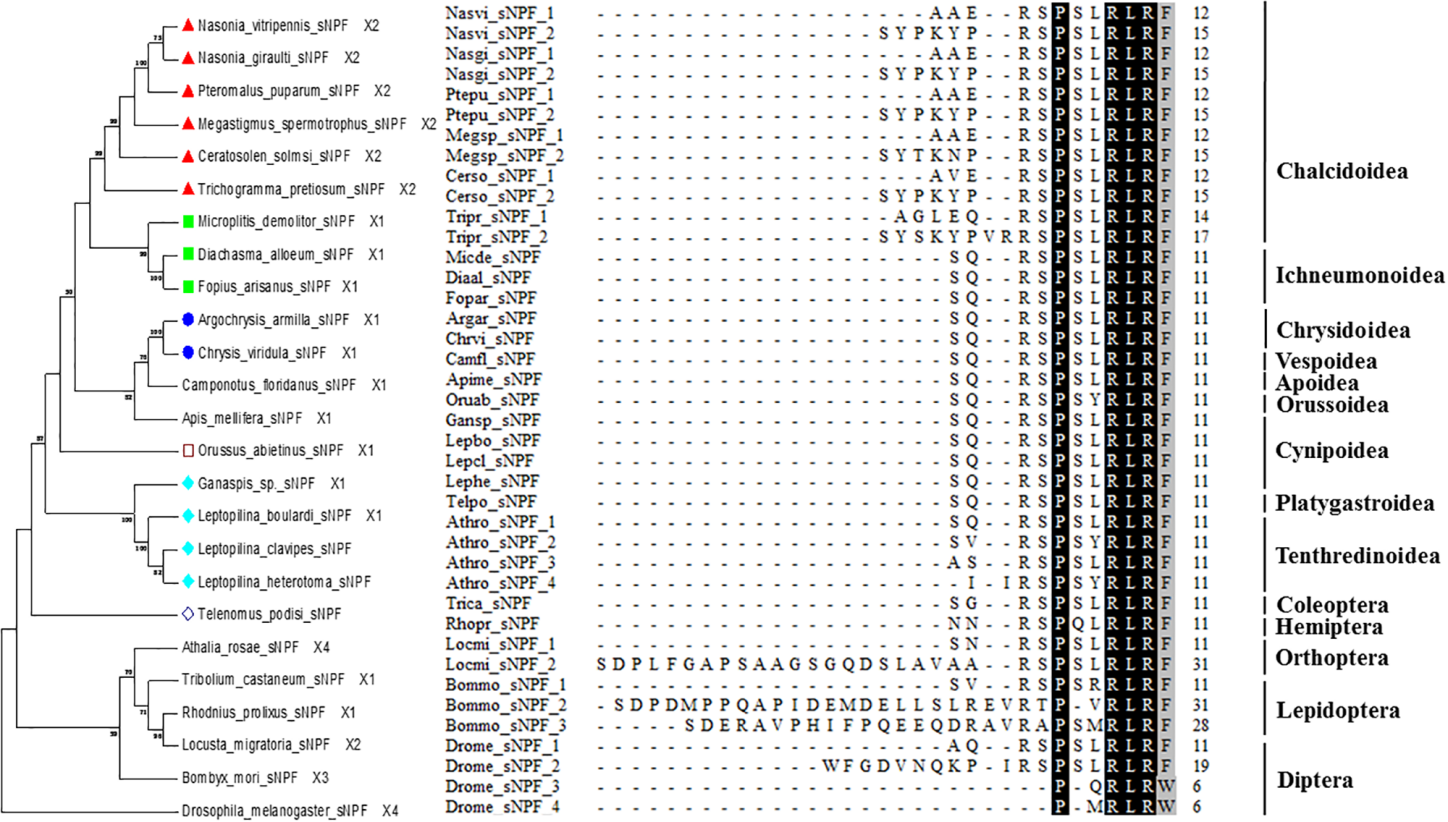
Phylogenetic tree of sNPF precursors and alignment analysis of sNPF sequences in parasitoid wasps and other insect species. Chrysidoidea sequences in phylogeny trees are indicated with blue circles; Ichneumonoidea sequences are indicated with light green squares; Chalcidoidea sequences are indicated with red triangles; Cynipoidea sequences are indicated with light blue rhombuses; Orussoidea with empty squares; Platygastroidea with empty rhombuses. Numbers above branches indicate phylogenies from amino acid sequences and only values above 50% are shown. The numbers of the paracopies carrying the motif are shown by the repeat numbers. Identities in alignments are highlighted in dark (100%) and in grey (80%~100%).

For comparison of the neuropeptides of parasitoid wasps and other insect species (*e*.*g*., *A*. *mellifera*, *L*. *migratoria*, *R*. *prolixus*, *T*. *castaneum*, *B*. *mori* and *D*. *melanogaster*; Fig 1), phylogeny analysis of the identified precursor genes and sequence alignment of the predicted mature peptides of insect neuropeptides were conducted (S2 Fig; Figs 2–5). Although it should be avoided for conducting phylogenetic trees of insects neuropeptide precursors for high diversity of precursor sequences (except mature peptides) in different insects in most cases (e.g., [33–34]), phylogenetic trees with neuropeptide precursors performed in this study showed that most of neuropeptide precursors from different parasitoid wasps were grouped together, probably because higher conservations occur among neuropeptide precursors from the very close taxa. The numbers of predicted neuropeptide genes from parasitoid wasp species were lower than those in other insect species (Fig 1). Some neuropeptides that are conserved in other insects were still highly conserved among parasitoid wasps, such as adipokinetic hormone (AKH), arginine–vasopressin-like peptide (AVLP), crustacean cardioactive peptide (CCAP), CCHamide, myosuppressin, and SIFamide (Panels ii, vi, x, xi, xxii and xxxiii in S2 Fig). All peptide sequences of AVLP and CCAP from different insect species were 100% identical (Panels vi and x in S2 Fig). Moreover, some interesting patterns regarding evolutionary orphysiological perspectives were found in this study.

### Phylogenetic evolutionary patterns of neuropeptides from hymenopterans

C-type Allatostatin (AST) is the arthropod homolog of vertebrate somatostatin. Three paralogue genes encoding C-type peptides (AST-C, AST-CC and AST-CCC) were found in arthropods [35]. In the present study, two paralogue genes encoding C-type allatostatins, AST-CC and AST-CCC, were identified in parasitoid wasps, as found in *Apis mellifera* (Figs 1, 2 and 3). AST-CC was present in Hymenoptera species and other insect groups (Fig 2). AST-CCC was found in both Hymenoptera and Hemimetabola insects (Fig 3). In contrast, AST-C has not been found in any species of parasitoid wasps. Arthropod C-type ASTs are known to be broadly conserved neuropeptides, with disulfide bridges between the cysteine residues at positions -2 and -9 from the C-terminus [35]. Insect AST-CC peptides share the NAVT/SCF consensus sequence; AST-C peptides from *T*. *castaneum*, *B*. *mori* and *D*. *melanogaster* share the PISCFamide consensus sequence, whereas AST-CCC peptides in *L*. *migratoria*, *R*. *prolixus* and most Hymenoptera species share the NAVSCFamide consensus sequence (Figs 2 and 3). Interestingly, AST-CCC peptides in Chrysidoidea species (*Argochrysis armilla*), *A*. *mellifera*, *Camponotus floridanus* and *Athalia rosae* were identical to those of other Hemimetabola insects (SYWKQCAFNAVSCFamide; Fig 3), whereas one or two amino acid substitutions occured within other parasitic wasp species (*e*.*g*., from Ichneumonoidea and Chalcidoidea). These finding suggest that a divergence of AST-CCC has occurred within Hymenoptera.

An interesting phenomenon was found for the PRXamide peptide group among different insect groups (Fig 4). Two gene families, cardioacceleratory peptide 2b (CAPA) and pyrokinin (PK), are known to encode three kinds of insect PRXamide peptides: periviscerokinins (PVKs), pyrokinins (PKs) and trypto-PKs [12, 36–38]. The encoding patterns of CAPA/PK genes were found to differ among Hymenoptera species and other insect species (Fig 4). CAPA precursors from Ichneumonoidea, Chalcidoidea and *A*. *mellifera* encode only one or two PVKs, whereas those in *Argochrysis armilla* and ants (*e*.*g*. *Camponotus floridanus*) encode two PVKs and one PK peptide. CAPA precursors in *R*. *prolixus*, *B*. *mori* and *D*. *melanogaster* encode PVKs and trypto-PKs; in contrast, those in *L*. *migratoria* and *T*. *castaneum* encode PVKs, trypto-PKs, and one PK peptide. PK precursors from Hymenoptera species, *B*. *mori* and *T*. *castaneum* encode trypto-PK and PK peptides, whereas *L*. *migratoria*, *R*. *prolixus*, *D*. *melanogaster* encode only PKs (Fig 4).

### Distinct patterns of neuropeptides between different groups of parasitoid wasps

Interestingly, sulfakinin (SK) was only found in cleptoparasitic wasps (Chrysidoidea: *Argochrysis armilla* and *Chrysis viridula*; Fig 1; Panel xxxiv in S2 Fig), and was not found in any other wasp groups based on BLAST results in NCBI. After the receptor genes for SK were checked based on BLAST analysis in NCBI, no gene encoding SK receptor was found from any parasitoid wasp species except from Chrysidoidea wasps. SK was first isolated from *Leucophaea madera* and was shown to stimulate hind gut contractions [39–40]. SKs are multifunctional neuropeptides found in many insects (*e*.*g*., *A*. *mellifera*, *Camponotus floridanus*, *L*. *migratoria*, *R*. *prolixus*, *T*. *castaneum*, *B*. *mori* and *D*. *melanogaster*; Fig 1) and are involved in food uptake [41]. It seems that the obvious lack of SK in endoparasitoid taxa could be related to the distinct food patterns in the parasitoid life. However, further studies are warranted to determine whether the absent of SK in endoparasitoid taxa is related to the distinct parasitoid life form or limited transcriptome data.

In particular, a few neuropeptides (*e*.*g*., elevenin, kinin, natalisin, neuropeptide-like precursor 1 (NPLP1), and trissin) were not found in any species of Chalcidoidea, but were present in other parasitoid wasp species and other insect species (Fig 1; Panels xvi, xxi, xxiii and xxvii S2 Fig). Among them, three neuropeptides, kinin, NPLP1, and trissin, were reported to be involved in insect feeding progress [3,18,42]. Insect kinins are small neuropeptides that function as myotropic, neuromodulatory, and diuretic hormones in the Malphigian tubules of insects [3]. NPLP1 was identified in the salivary glands of *R*. *prolixus* [18], suggesting that it plays a role in the hormonal control of salivary secretion. Trissin is dominantly expressed in the frontal ganglion of *B*. *mori* [42], indicating its possible role in the regulation of foregut–midgut contractions and food intake. Whether the lack of these neuropeptides in parasitoid wasps is related to their distinct feeding patterns or limited transcriptome data needs to be investigated in the future.

Elevenin was first identified as a neuropeptide from the abdominal ganglion of the gastropod mollusk *Aplysia californica* [43]. Similar neuropeptide precursors have been identified from many insect species [12,13]. At present, only one report is available regarding the physiological role of elevenin [44]. In the planthopper *Nilaparvata lugens*, elevenin is known to regulate body color via a G protein-coupled receptor NlA42, which is expressed in the abdominal integument; this might indicate the direct action of elevenin on the melanization of the cuticles of *N*. *lugens* [44]. In the present study, elevenin was not found in any Chalcidoidea species, as well as in *D*. *melanogaster* and *B*. *mori* (Fig 1; Panel xvi in S2 Fig). Phylogenetic analysis of insect elevenin precursor genes showed a significant divergence between Hymenoptera and other insects (Panel xvi in S2 Fig). Sequence alignment of insect elevenin peptides showed high variations among different insect species, which only share a C-terminus motif CRGXXX and two cysteine residues (Panel xvi in S2 Fig). However, the elevenin gene sequences were highly conserved within the same subfamily of parasitoid wasps, *e*.*g*., Microgastrinae, Opiinae and Chrysidini, that they can be used a molecular marker for species identification between different subfamilies of small parasitoid wasps (*e*.*g*., of Braconidae family; Panel xvi in S2 Fig). Like mentioned before, information reagrding insect elevenin is limited, and hence the determination of whether elevenin was not found in Chalcidoidea wasps because of their distinct evolutionary/physiological progress, or the remarkable diversity in these wasps, or limited transcriptome data is not possible.

Natalisin was first identified as a functional neuropeptide associated with sexual activity and fecundity in insects [45]. In the present study, natalisin was found in five Ichneumonoidea species and other Hymenoptera species (*e*.*g*., *Athalia rosae* and *Camponotus floridanus*), but not in any other parasitoid species except Ichneumonoidea (Panel xxi in S2 Fig). High variants in copy numbers and peptide sequences of natalisin occur between Hymenoptera species and other insects, as well as among different species from Ichneumonoidea, suggesting that natalisin was not found in some parasitoid wasps because of the remarkable diversity of natalisin in parasitoid wasps and limited transcriptome data.

Several neuropeptides showed vast sequence differences between between Ichneumonoidea and Chalcidoidea, the two major superfamilies of parasitoid wasps. Short neuropeptide F (sNPF) was first identified in *Aedes aegypti* [46]. The main functions of sNPF is likely the regulation of feeding behavior [47]. sNPFs are widespread among parasitoid wasps. sNPF precursors were found in 17 species of six superfamilies (Figs 1 and 5). This neuropeptide is conserved in parasitoid wasps and possesses a C-terminal motif–RSPSL/YRLRFamide (Fig 5). Two distinct peptides were predicted from sNPF precursors in all six Chalcidoidea species, whereas only one sNPF peptide was found from each of the other 11 parasitoid species. All the predicted precursors of parasitoid wasp species except for the six Chalcidoidea species possess the same or similar C-terminal motifs as *A*. *mellifera*_sNPF (-SQRSPSLRLRFamide; Fig 5). High variations in the N-terminal sequence of sNPF peptides were found between Chalcidoidea species and other Hymenoptera insects.

Eclosion hormone (EH) and ecdysis triggering hormone (ETH) are two of the major components of the peptidergic circuit controlling ecdysis in insects [48]. ETH peptide is highly conserved among Hymenoptera species (Panel ix in S2 Fig), whereas EH peptides remarkably differed among Hymenoptera species, especially between Ichneumonoidea and Chalcidoidea (Panel xv in S2 Fig). EH is a long peptide hormone with 6 cysteine residues forming three disulphide bridges in most insects. However, only 4 cysteine residues were found in the EHs of Chalcidoidea species. A low level of identity was found for putative EH sequences between *Nasonia vitripennis* and *Fopius arisanus* (Panel xv in S2 Fig), with an identity score of 45%, which was calculated using GeneDoc.

The phylogenetic and alignment analyses of the above neuropeptides (elevenin, kinin, natalisin, NPLP1, trissin, sNPF, and EH), suggested that some of these neuropeptides not found in Chalcidoidea or having considerably diverged between those in Chalcidoidea and other Hymenoptera species, might be related to different evolutionary or physiological patterns in Chalcidoidea species. However, further studies are warranted to explore the relationships of sequence patterns and functional roles of these neuropeptides in parasitoid wasps.

## Conclusions

In the present study, publicly accessible databases and a well-established workflow were used for the prediction of neuropeptide sequences. In all, 517 precursors from 24 parasitoid wasp species were identified. Among them, five neuropeptides, *i*.*e*., CNMa, FMRFa, ITG-like, ITPL and orcokinin B, were identified for the first time in parasitoid wasps, to our knowledge.

Comparisons of neuropeptides among parasitoid wasps and other insect species revealed some interesting patterns regarding the evolutionary or physiological perspectives and might be useful for investigating the phylogenetic and divergence relationships among the Hymenoptera and other insect groups. Phylogenetic analysis of C-type ASTs suggested the divergence of AST-CCC within Hymenoptera. Further, the encoding patterns of CAPA/PK family genes were different between Hymenoptera species and other insect species.

Some neuropeptides that were not found or were considerably divergent in some superfamilies of parasitoid wasps might be related to distinct feeding habits or other physiological processes in some parasitoid groups. Sulfakinin was not found in any parasitoid wasp species except cleptoparasitic wasps. A few neuropeptides (*e*.*g*., Elevenin, kinin, NTL, NPLP1, and Trissin) were not found in any species of Chalcidoidea but were present in other parasitoid wasp species and other insect species. Several neuropeptides (*e*.*g*., sNPF and EH) sequences show considerable difference between Chalcidoidea and other Hymenoptera insects. However, further studies are warranted for determining whether these patterns are due to the distinct parasitoid life or limited transcriptome data.

Analysis of neuropeptidomes in parasitoid wasps can be useful for better understanding the phylogenetic evolution of Hymenoptera and for conducting in-depth analysis of the physiological roles of neuropeptide signaling systems in parasitoid wasps.

## Supporting information

S1 Table List of putative neuropeptide precursors of parasitoid wasps via *in silico* mining. (XLSX)

S1 Fig Predicted structures of neuropeptide precursors of parasitoid wasps. Predicted signal peptides (highlighted in yellow), cleavage signals (red), putative bioactive mature peptides (light blue), amidation signals (pink), N-terminal N-terminal Glutamate (Q) to Pyroglutamate (pQ) conversion (green) and cysteine residues (deep yellow) are indicated.
(DOCX)

S2 Fig Phylogenetic tree of neuropeptide precursors and alignment analysis of putative mature neuropeptide sequences in parasitoid wasps and other insect species. Chrysidoidea sequences in phylogeny trees are indicated with blue circles; Ichneumonoidea sequences are indicated with light green squares; Chalcidoidea sequences are indicated with red triangles; Cynipoidea sequences are indicated with light blue rhombuses; Orussoidea with empty squares; Platygastroidea with empty rhombuses. Numbers above branches indicate phylogenies from amino acid sequences and only values above 50% are shown. The numbers of the paracopies carrying the motif are shown by the repeat numbers, and the numbers in parentheses means the numbers of the paracopies predicted from a partial precursor. Identities in alignments are highlighted in dark (100%) and in grey (80%~100%).
(PPTX)
