# Supplementary material for: In silico prediction of neuropeptides in Hymenoptera parasitoid wasps
Source: PLoS One. 2018 Feb 28;13(2):e0193561. doi: 10.1371/journal.pone.0193561 (PMC5831470; doi:10.1371/journal.pone.0193561)
Supplement: S1 Fig — Predicted signal peptides (highlighted in yellow), cleavage signals (red), putative bioactive mature peptides (light blue), amidation signals (pink), N-terminal N-terminal Glutamate (Q) to Pyroglutamate (pQ) conversion (green) and cysteine residues (deep yellow) are indicated. (DOCX) [file pone.0193561.s002.docx]

**Supplementary data S2.** **Predicted structures of neuropeptide precursors of parasitoid wasps.** Predicted signal peptides (highlighted in yellow), cleavage signals (red), putative bioactive mature peptides (light blue), amidation signals (pink), N-terminal N-terminal Glutamate (Q) to Pyroglutamate (pQ) conversion (green) and cysteine residues (deep yellow) are indicated.

>Ceratosolen solmsi_AKH/corazonin-relate peptide

MSKFLRHFFTLLLIIFCIAHLTVAQVTFSKGWGPGKRAALYDSECRINPKSLALMFHTIMAEVKYLMACDHQATINYLQTVEH

>Copidosoma floridanum_AKH/corazonin-relate peptide

MTKTIAYRFTFLVCVLCYLQLAVAQVTFSKGWGPGKRAALYDDDCRVNSKTLTLMFHTLMAELKRLMACDHQATVNYLQSVEH

>Megastigmus spermotrophus_AKH/corazonin-relate peptide

MSRSLRYFFVLLVTIFWITQLAMAQVTFSKGWGPGKRAALYDSDCRVNAKSLAMIHSLMVEIKHLLACDHQAAVNYLQTVDH

>Nasonia giraulti_AKH/corazonin-relate peptide

MGRRLSIGLAAIVILFSCMLHFALAQVTFSKGWGPGKRSALYETDCSRVNYKSLALLFHTLMAEVKHLMACDHQATVNYLQSVERQ

>Nasonia vitripennis_AKH/corazonin-relate peptide

MGRRLSIGLAAAAILFSCMLHFALAQVTFSKGWGPGKRSALYETDCSRVNYKSLALLFHTLLAEVKHLMACDHQATVNYLQSVERQ

>Pteromalus puparum_AKH/corazonin-relate peptide

MGRRLSIGLAAVAILVSCMLHLTLAQVTFSKGWGPGKRSALYETDCSRVNYKSLALLFHTLMAEVKHLMACDHQATANYLQSVERQ

>Telenomus podisi_AKH/corazonin-relate peptide

MMRPNKLARVFISVYLSFCLLHLVVSQVTYSRGWGPGKRAINDECSVNILLLRSLVNELKAMIYRDIQAQPNIQSI

>Trichogramma pretiosum_AKH/corazonin-relate peptide

MSKHSRLVICFFFIVCLLQLSMSQVTLSRGWGPGAGKRAVSYDSDCQVNAKSLQLIFHLLMPEVKRLLGCDHQTMINYLQGLDRP

>Argochrysis armilla_Adipokinetic hormone

MDLCCYAEHSFKLPCRSLLCAAAWFCKVSSGVLVFLLLLISMLHRGTEAQLNFSTGWGKRSHNLKMDTTTSNCFRSGRPSFQQLLDLYNLIQIEAERILDCRKLNE

>Chrysis viridula_Adipokinetic hormone

MFWKVSSGVLVFLFFSISMLHRGTEAQLNFSTGWGKRSHNLKMDTTTSNCFRSGKPSLQHLLDIYNLIQIEAERVLDCRRLNE

>Cotesia glomerata_Adipokinetic hormone

MFSFKCSTILMLEVLIICILCRAISRVESQLNFSTGWGKRNLQHQCRTWNNKIQSIPYCLFNLSGSNYRRSSRIFNKSIIRVLHSSLVV

>Cotesia rubecula_Adipokinetic hormone

MFSFKCSTILMFEVIIICILCRAISRVESQLNFSTGWGKRNLQHQCRTWNNKIHSTPYRLFNLSGSNYRRSSRISNKSIIRVLHSSLVV

>Cotesia vestalis_Adipokinetic hormone

MFSFKCSTILMFEVIIICILCRAISSVESQLNFSTGWGKRNIPLQCRAWNNKLQSTPYCLFNLSGSNYRRSSRLFNKSIIR

>Diachasma alloeum_Adipokinetic hormone

MHCKILPGVLAILLIGCLTFLFIGTTEAQLNFSTGWGKRNKNFPGENQSDTGCSVTQGKPPLEILLNLYNLIQIEAKRIINCEKMNQ

>Diachasmimorpha longicaudata_Adipokinetic hormone

MHCKIPSGILAILLIGCFTLLFIGTTEAQLTFSTGWGKRNKNFLAENESNTRCSATPGKPPLEILLNLYNLIQIEAKRMINCEKMN

>Fopius arisanus_Adipokinetic hormone

MHNKIPPGILAILLIGCITLLFIGTTEAQLTFSTGWGKRNKNFAVENGSGTRCSEAQGKPPLEILVNLYNLIQNEAKRVINCEKMNQ

>Leptopilina heterotoma_Adipokinetic hormone

MLCYRCLFSRCPYSLTRFIICFLLLMNVTAQLNFSTGWGKRNHELRINKNSLHSSELRLSMKKLINVYRLLNTD

>Megastigmus spermotrophus_Adipokinetic hormone

MNCIKLRLSFLAIGVILCCILITITEAQLTFSTGWGKRSGPIVSAASGEPDPDVFFRRYYRRLRKMQGDGNSSQ

>Nasonia giraulti_Adipokinetic hormone

MNCKSLLALGLCLCVVLLQTRAAEGQLNFSTGWGKRSSHLLQPARASSSSSSSAASSGIDRPRQQADLDNFLQRFYRRLRKIEAQRLANSQV

>Nasonia vitripennis_Adipokinetic hormone

MNCRSLLALGLCLCVVLVQTRAAEGQLNFSTGWGKRSSHLLQPARASSSSSSSSSSSSTSAAAAAAASSGIGRPRQQADLDYFLQRYYRRLRKIEAQRLANSQV

>Orussus abietinus_Adipokinetic hormone

MCRKIPSGLLAVVLLVCLILVTNVEAQLNFSTGWGKRSQILSTRGGNSPCSSQGRPTLDQLLGLYNLIQAEAQKIVDCQKLTK

>Pteromalus puparum_Adipokinetic hormone_partial

...GLCLCIVLLQSRAAEGQLNFSTGWGKRSSHLLEPVQGSSSSASSSSASSASSGIGRPRQQADLDFFLQRYYRHLRKIEAQRLANSQ

>Telenomus podisi_Adipokinetic hormone

MFIKPQLSLLTIGIFCIFYLFVPTEAQLNFSTGWGKRNIDLDSKPPLFSYLTQKQRVQLQQLQQFYNFLKLMDDDGRISSSKGNDPRNNINVLPAGI

>Argochrysis armilla_Allatostatin-A

MPLKIVAFCAFGAFGAFVAAEEISSSSLHSQRGSINGLTFDEPSDKRAYTYVSEYKRLPVYNFGIGKRWIDNSDDKRSRTYSFGLGKRGRNYDFGLGKRTEYAEYRPVGLTSLDYLLPVENYEGYQLRDGDNGDDMYEKKRAGYVYRFGLGKRAWNAGSESIGTGKRPNEDVLHRYNFGIGKRDAEDDESNQ

>Ceratosolen solmsi_Allatostatin-A

MRIRIIVFCVLSILGGWSVAMDETASSLAGGSSSSALPNSASSSLHGPRFSSLVDPRDQVLGGKRAYTYRSEFKRLPLYQFGLGKRWAQGIEGKRTQPFSFGLGKRTRQYSFGLGKRSNYEDSDDTSRYGFGYIPNDQYDAYAQRNAFENYLEAKRTGGFNFGLGKREAEINDDEKLPAKNLRDKYLFGLGKRLYEEDQDEMDEEEA

>Chrysis viridula_Allatostatin-A_partial

...ENKRAGYVYRFGLGKRAWNAVSESIGAGKRPNEDVLHRYNFGIGKRDVEDDEQNQ

>Copidosoma floridanum_Allatostatin_partial

...MPAKPYTFGLGKRSSYDEATRFSPKYQNLMSNVYTQKLLENYLESKRASAGGFNFGLGKRDVDYDMPDRLTVKHSRDNRYGFGLGKRDGMNPEEYYDEDDASVNSAQFV

>Cotesia glomerata_Allatostatin-A

MSKRKELSVSILLIIYLFVNCVTGFIDDSTSVVERSSREINNRPLCTAKKDYDLITEYKRLPENRYVAEYKRFPEYRYSFGIGKRWYDDSKRNTPYSFGIGKRYQMDKMDQMEKNKLHQFGLFNDYLSVGSDDNYPVIQDQQNKRGAVPKSRNYDFGVGKRNIHIFSSIKQPQHINPDDKHDSSDNYNNDIWEQD

>Cotesia rubecula_Allatostatin-A

MSKRKELSFSILLIIYLFVNCVTGFIDERSSLEINNRPLCTAKKDYDLITEYKRLPENRYVAEYKRFPEYRYSFGIGKRWYDDSKRNTPYSFGIGKRYQMDKMDKIDQMEKNKLHQFGLFNDYLSVGSDDNYPVIQDQQQNKRGAIPKSRNYDFGVGKRNIHIFSSIKPQHINPDNHESSENYNNDIWEQD

>Diachasma alloeum_Allatostatin-A

MIQRKLFNITLMFALTLSINIATSAADEPTATPLQEAHQRGCVSPKKDYDFITEYKRLPQYRYLAAYKRFPDYLYSFGIGKRFDDNIKRNSPYSFGVGKRNTFPRLSFLRVPLAYALPLDTKSYDSNLIVDDSAGTKRNAPSHAYGFGIGKRTPLLLNDDFDADNRGLNDDRKFTDDGPLWDQF

>Fopius arisanus_Allatostatin-A

MIQRQFFHITLILALALMMNIGISAADEPRVTVPVQGIHQRGCEAPKKDYDLITEYKRLPQYRYLAAYKRFPDYLYSFGIGKRFDDSIKRNSPYSFGVGKRNAFPRLYPVRLPLEYVPLDGKNYDSNLIIDDSGEIKRNSPSHAYGFGIGKRNLQLFNGDFDSDNRELSDDRKIADDGQLWDQF

>Ganaspis sp._Allatostatin-A

MMRIQVIMFCLLSMLSYFSSALEANTYPDKDNAVNFDYPDNGRTSQYLPMKRYTYVSEYKRLPLYNFGLGKRSRPYEFGVGKRSKNYGFGVGKRYNFDDQSFDYLSDAAYDTNFEENQLQHKRTPQRFSFGVGKRSSETISSVPEYLINNIRKEKNNTHDINLYEVYDLMRRLNKEEALSQSP

>Leptopilina clavipes_Allatostatin-A_partial

...MKRYTYVSEYKRLPLYNFGIGKRSRPYEFGVGRRSKNYGFGVGKRFNFDDQSLDYLSDASYDPSINDFEDNQSQYKRSPQRFSFGVGKRSSESLINNIKKEKNNTNDLNLYEVYDLMRRLNKGDALTQNP

>Leptopilina heterotoma_Allatostatin-A

MQLFHIIQSVCNIPHRKWGLSSRQILYRKASRRSRCHSRADFKRTRHFTSATGIFINFRRIGIIYSGIMKMTVFIFSVLSTLSIFCSALEASTYTDRDNNANLDYAANSRSSQYLPMKRYTYVSEYKRLPLYNFGIGKRSRPYEFGVGRRSKTYGFGVGKRFNYDDQSMDYLSDASYDPSLNDFEDGQPQYKRSPQRFSFGVGKRSSESSYPESLVNNIKKEKNNTHDLNLYEVYDLMRRLNKGDAISQNP

>Microplitis demolitor_Allatostatin-A

MNKRKLFIVTILVICLSINYVTGLMEDSTSVIDRSGREINSNQRPLCAAKKDYDFITEYKRLPEHRYVTEYKRFPEYRYSFGIGKRWYDDSKRNTPYSFGIGKRYQMENKLHPFRFFSDYLSIDNGNYPVNVNDVIPDQEENKRAVPARSYDFGVGKRNNIFSSIQQQQQQKKHIDDNDKNQDNIKSQIWEQN

>Nasonia giraulti_Allatostatin-A

MSSSNLSGTAMSLTIFCVLSILGGTSVAMDEQSSASSSSGVSSSSLSSSHGPHFSPLVDPRDQMVGGKRAYTYRSEYKRLPIYQFGLGKRWVDDKRSQPFSFGLGKRTRPYSFGLGKRSSYSEDDGSRYDLGLSYLIQPSDLYEQLAQRDALENYLQQQQAIKRTGGFNFGLGKRDAEMNEGMMREDGLHEKVPVKHSRDKYLFGLGKRFYEPATMQDDEDEEMLEDA

>Nasonia vitripennis_Allatostatin-A

MSSSNLSGTAMSLTIFCVLSILGGTSVAMDEQPSASSSSGVSSSSSSSSHGPHFSPLVDPREQIVGGKRAYTYRSEYKRLPIYQFGLGKRWVDDKRSQPFSFGLGKRTRPYSFGLGKRSSYSEDDDSRYGLDLSYLIQPSDLYEQLAQRDALENYLQQQQAIKRTGGFNFGLGKRDAEMNEGMMREDGLHEKVPVKHSRDKYLFGLGKRFYEPATMQDDEDEEMLEDA

>Pteromalus puparum_Allatostatin-A

MSSSNLCGTAMSLTIFCVLSILGGTSVAMDEQASGSSSSGVSSSSSSSSHGPHFSPLVDPRDQIVGGKRAYTYRSEYKRLPIYQFGLGKRWVDDKRSQPFSFGLGKRTRPYSFGLGKRSSYSEDDGSRYGLGLSYLIQPSDLYEQLAQRDALENYLQQQQQAIKRTGGFNFGLGKRDAEMNEGMMRNDGLNEKVPVKHSRDKYLFGLGKRFYEPATMQEDEDEEQQMIEDA

>Telenomus podisi_Allatostatin-A

MILSTAVARIILLYLVSGFGKFVVSMEESAESLEVNNNEIDDIDSYDFNEPQNKRTYTYVSEYKRLPVYNFGIGKRQTYGDKRIKPYSFGIGKRSQGYTFGVGKRRYPMLNFDYPELSSYEARFHPDLINLLEAKRNNLKYSFGVGKRTLELMPAFTDNLATNPKRGSKYTFGVGKKSLEDPMQS

>Argochrysis armilla_Allatostatin-CC_partial

...TAGNSKAVVPASEQTMDLQRRGQAKGRIYWRCYFNAVTCFKRK

>Ceratosolen solmsi_Allatostatin-CC

MRAAGPILALLVALTVLGVPAGTGARAIEEALDEYPDYEYQRTTSKRAALLLDRLLVALQKAVENNELEKGGSKPFVRSLPEISEMPDSPRMHLSDEKIMDLQRRGQAKGRIYWRCYFNAVTCFKRK

>Copidosoma floridanum_Allatostatin-CC

MTIISSSGGSFFALALLCALAGVLGRVLDKRSAEATGTPELYPDYPDYQKAMPKRAALLLDRLLVALQKAVDNEEIGKNGYKTYARTLPNYSDIPESEHGMQLTDERTMDLQRRGQAKGRVYWRCYLNAVTCFKRK

>Cotesia glomerata_Allatostatin-CC

MGMIKFCSIALLMTTVMTTGTARYIPASNYILLEKLINQYDPSGVNYDYGEDDQDNQVALKKDALLLEKLMLTLQKNAEDDSNAIELQDRKELVLNDPRLQKFMIRRNRSENRKTGMDLQRRGRINGSVYWKCYFNAVTCF

>Cotesia rubecula_Allatostatin-CC

MGMIKFCSIALLMTTVMTTGTARYIPASNYILLEKLINQYDPSGVNYDYGEDDQDNQMDLQRRGRINGSVYWKCYFNAVTCF

>Cotesia vestalis_Allatostatin-CC

MGMIKFCSIALLMTTVMTTGTARYIPASNYILLEKLINQYDPSGVNYDYGEDDQDNQVALKKDALLLEKLMLTLQKNVEDDSNAIDLQDRKELVLNDPRLQKFMIRRNRSENRKTGMDLQRRGRINGSVYWKCYFNAVTCF

>Diachasma alloeum_Allatostatin-CC

MGMSLVTVIFMVVLIVTTVARVLPKRSLSDNLSESYDYEDYSRSQERYPVEEKRAALLLDRLMVALQKAVDGQGMGDSRGGKEVLRAKPRVGALIGPQSGGSKSGMDLQRRGPANGSVYWRCYFNAVTCFKRK

>Fopius arisanus_Allatostatin-CC

MTMSLVTVIFMVTLIVTTTVARVLPKRSLSDNLSESYDYEDYPGKQEQYPVATKRAALLLDRLMVALQKAVDEQGIGDSRGGRDILRSKSRLGGTIGSQSGPSKSGMDLQRRGPVNGSVYWRCYFNAVTCFKKK

>Leptopilina clavipes_Allatostatin-CC

MQKRAALLLDRLMIALQKAIENEDSVGGGSSGKYYVKSENQLRESTRQNIPLTDEKTMDLQRRGQAKGRVYWRCYFNAVTCFRR

>Megastigmus spermotrophus_Allatostatin-CC

MTGTGSFVALATALTLLCNVDGGSIGLHTLAKRNTGTSEDNTAEYSDYEYQRVMPKRAALLLDKLLVALQKAVDNDEIGRESSKSYVNSLPDFSDMSDSQRMQFSDEKTMDLQRRGQAKGRVYWRCYFNAVTCFKRK

>Microplitis demolitor_Allatostatin-CC

MEMIKFLSKVLLMTSFMTTVTVARYIPANNYILLEKLINRYDPSVINYDYIEDDPDNQVAARKDALLLEKLMLTLQKNTEEDSQMMDFQGRKNSLLEEPQFQKLMIRRNRSENRKTGMDLQRRGRINGSVYWKCYFNAVTCF

>Nasonia giraulti_Allatostatin-CC

MTSFGCCIFLALATALTILGIVSGSPRGHALAKRSADANAIEYPDHEYQRVMPKRAALLLDRLLVALQKAVENDEVGKENSNSYVRSLPDFSDMPDSQRMQLSNEKTMDLQRRGQAKGRVYWRCYFNAVTCFKRK

>Nasonia vitripennis_Allatostatin-CC

MTSSGCRTFLALATALTILGTVSGSPRGHALAKRSADANVIEYPDYEYQRVMPKRAALLLDRLLVALQKAVENDDVGKENSNSYVRSLPNFSDMPDSQRMQLSNEKTMDLQRRGQAKGRVYWRCYFNAVTCFKRK

>Orussus abietinus_Allatostatin-CC_partial

...LSGGGGGERSISRAPSHGPVSGSRIVPAADGQTMDLQRRGQARGSVYWRCYFNAVSCFKRK

>Psyttalia lounsburyi_Allatostatin-CC

MSLVTVIFMVILIITTTVARALPKRSLSDNLSESYDYEDYSGKEERYPVASKRAALLLDRLMVALQKAVDEQGTGDSRGGKEMPRTKPRVEALVGLHGVASPGMDLQRRGPSASGNVYWRCYFNAVSCFKKK

>Trichogramma pretiosum_Allatostatin-CC

MVLSGPHPGFAAVLIIICMIHGLLGMALNDDSMDPLDVQLETYSNEEPRVLPKRAALLLDKLLVALQKAVDKDEIGKEGSKSYARMQSDFPDFPESQKFLLADDKTMDLQRRGQAKGRVYWRCYFNAVTCFKKK

>Telenomus podisi_Allatostatin-CC

MPVMPKRAALFLDRLMVALHEAMENNISGEGGGGGSYSSRESTIPLEAKSSDYIVDAKSKGNPKRIGSKNFWRCYFNAVTCF

>Argochrysis armilla_Allatostatin-CCC

MLSLRGAVSLAVCLVLLFDWSAALPAPEKERYLNEVDLVDDDGSIETALINYLFTKQIVQRLRNQLDIGDLQRKRSYWKQCAFNAVSCFGK

>Ceratosolen solmsi_Allatostatin-CCC

MMSLKIVACAIVALVVFVDWSTPLPAGDKEALMNGLNLQMVGDEEGAAIERDLLNYLVGRRFVKRLRSQVDVDDLQRKRNYWRQCAFNAVSCFGK

>Cotesia glomerata_Allatostatin-CCC

MMSRIIFLAAIVVLILVGWSAALPANKERYLNELDLVDDDGTMDTALINYLFTKQIVKRLRNQMDIGDLQRKRTYWKQCAFNAVSCFG

>Cotesia rubecula_Allatostatin-CCC

MQVSREMLYIAVALACVIGLSSALPADKDRFLKELDLVDDDGSMDTALINYLFTKQMVKRLHNQIDIGDLQRKRTYWKQCAFNAVSCFGK

>Cotesia vestalis_Allatostatin-CCC

MMSRITFLAVIAVIILVGWSAALPANKERYLNELDLVDDDGTMDTALINYLFTKQIVKRLRNQMDIGDLQRKRTYWKQCAFNAVSCFG

>Diachasma alloeum_Allatostatin-CCC

MQLSREMLCIAVALVCVICLSSALPADKERFLNELDLVDDDGSMETALINYLFTKQMVKRLHNQIDIGDLQRKRTYWKQCAFNAVSCFGK

>Diachasmimorpha longicaudata_Allatostatin-CCC

MMSRITFLAAIAVLILVGWSAALPANKERYLNELDLVDDDGTMDTALINYLFTKQIVKRLRNQMDIGDLQRKRTYWKQCAFNAVSCFG

>Fopius arisanus_Allatostatin-CCC

MQLSREMLYVAVALVCFIGLSSGLPTDKERFLNELDLVDDDGSMDTALINYLFTKQMVKRLHNQIDIGDLQRKRTYWKQCAFNAVSCFGK

>Leptopilina boulardi_Allatostatin-CCC

MVSVRVMACFGIVLITLVHWSTCYPTVDKESLQNEIDTYDDNGPENAALLYKLLRRLGTQLAVGELQRKRSYWKQCAFNAVSCYGK

>Leptopilina clavipes_Allatostatin-CCC

MVSVRVMACFGIVLITLVHWSTSYPTVDKESLQNELEAYDDNGPENAALLYKLLRRLGTQLAVGELQRKRSYWKQCAFNAVSCYGK

>Megastigmus spermotrophus_Allatostatin-CCC_partial

...VKRLRNSQASDSDDYQRKRNYWKQCAFNAVSCFGK

>Microplitis demolitor_Allatostatin-CCC

MMMSRIIFFVAIALVVFVGWTSALPADKERFLNELDLVDDDGSMDTALINYLFTKQIVKRLRSQMDIGDLQRKRTYWKQCAFNAVSCFGK

>Nasonia giraulti_Allatostatin-CCC

MMSLRIAVCTAVALVVLVDWSTALPAGDKEALMNGLNMMDDEEGAAVEKDLLNYLVGRRFVKRLRNQADVDDLQRKRNYWRQCAFNAVSCFGK

>Nasonia vitripennis_Allatostatin-CCC

MMSLRIAVCTAVALVVLVDWSTALPAGDKEALMNGLNMMGDEEGAAVERDLLNYLVGRRFVKRLRNQADVDDLQRKRNYWRQCAFNAVSCFGK

>Orussus abietinus_Allatostatin-CCC

MVSLRSTIRLTMVLVALLGLSSGLPTPDKQRFINDLDLVDDDGSIETALINYLFTKQLVQRLHNRMDIGDLQRKRSYWRQCAFNAVSCFGK

>Pteromalus puparum_Allatostatin-CCC

MMSLRIAVCTAVALVVLVDWSTALPAGDKEALMNGLNMMGDEEGAAVERDLLNYLVGRRFVKRLRSQADVDDLQRKRNYWRQCAFNAVSCFGK

>Telenomus podisi_Allatostatin-CCC

MIDWIMAAPMIEEDVKLSDIRNQLTEAEEREALDNLVSYIIARKVLGLHSPDTMYEMQKKRSYWKLCAVNAVSCFGKK

>Trichogramma pretiosum_Allatostatin-CCC

MLLKLVVCTIIALVVFIDWTAALPAGDKEALMNGLNLQMVGGEDDGSSAAVERDLLNFLVGRRFVKRLRSQADIDDLQRKRNYWRQCAFNAVSCFGK

>Argochrysis armilla_Allatotropin

MRRGLALAASLLMILASVEGSRTSRTAAHLDKHRTKQREIRGYKPEYISTAIGFGKREGSNNYGLPKLDERDKILLSLLATIPEGIPLESLLRQRNAKFGPSPMADDDGRRAATLLRLSPERRIVL

>Chrysis viridula_Allatotropin

MLRGLALAASLLMILASVECSRTSRTSAHLDRHRTKQREIRGYKPEYISTAIGFGKREGSNIYGLPKLNEREKILLSLLETIPDGIPLESLLRQRNTKIGSSPMADDDGRRTATLLRLSPDRRIVL

>Diachasma alloeum_Allatotropin

MKGIALLMILVIYLRTINGQNSEEKYNRGNAQLSRAHARAREVRGIRPGNLQTARDFGKRDQRFEEMKDVQLDRQTKRYPSQWFLEQLRRHPGITREYERILLKAGDRRNLTTKILLESIKIL

>Fopius arisanus_Allatotropin

MILVISLKPSSGQSSDEKYTRGNYQMTKTQTRAREVRGIRPGSLQTARDFGKRNDRLLELKDVFDRQLKRYPIHWLLELQRYPRVTRQYERALVNTEDQRNSITRLLLQSL

>Megastigmus spermotrophus_Allatotropin

MRVTIFVSLALVGVLLVEAASSGNCVHCVRWRSKPREIRGFQPKYISTAIGFGKREIAKQDKYEKIMLLLLQNSPQSVPASLLVHEMRKNPELAKRIVRKILDAGQEDKNPGKLSTAELFKSERTISFF

>Nasonia giraulti_Allatotropin

MRVSVFVSVALVAVLLAEGTSSGSCEHFAKPRTKPREIRGFQPEYISTAYGFGKREIAKQDKYEKIMLLLLQNSPQSIPASWFLHEMRQNPELAKRIVRKIVDGGEEDNPGKLSTAELFKSERKISFF

>Nasonia vitripennis_Allatotropin

MRVSVFVSVALVAVLLAEGTSSGSCEHFAKPRTKPREIRGFQPEYISTAYGFGKREIAKQDKYEKIMLLLLQNSPQSIPASWFLHEMRQNPELAKRIVRKIVDGGEEDNPGKLSTAELFKSERKISFF

>Orussus abietinus_Allatotropin_partial

...LGLLVVEGAKSRNSDYSKHRTKPREIRGFKPNILSTAYGFGKRESSYELSKNDKLERILLSLLQQLP

>Pteromalus puparum_Allatotropin

MRVSVFVSVALVAVLLAEGTSSGSCVHFEKPRTKPREIRGFQPEYISTAYGFGKREIAKQDKYEKIMLLLLQNSPQSIPASWFLHEMRQNPELAKRIVRKIVDGGEEDNPGKLSTAELFKSERKISFF

>Trichogramma pretiosum_Allatotropin

MRFNLLIYYAFVGIVLTATIHFGNCGKPRTKPREIRGYQPDQLMTAISFGKRDYSNDNKFEKIFMLLLQNDSSYDGFTVRWLLREMKRNPEMAKWVLKKLSGNNSDKQSMNDSAADEFDSKKPMSYF

>Chrysis viridula_Arginine–vasopressin-like peptide_partial (the neurophysin domain with the characteristic 14 cysteine residues is shown in grey)

...AMHKNECPSCGPNRLGQCFGPHICCGPSIGCFIGTPETYKCRKESLFSRPCVAGYAMCRGNSARCAANGICCSQETCHMDSTCRISDVDTRGRKEEGVDVA

>Copidosoma floridanum_Arginine–vasopressin-like peptide

MLKVSLVLLALVSVAYSCLITNCPRGGKRGDPTFLLENIARECPACGHEEQGRCFGPHICCGPSMGCHIGTLETLRCRKESLYSRPCVAGFAMCQGNSGRCAANGICCSQESCFTDSACKLVDESGNDRKIGAEFGAFLLENAGANERILR

>Cotesia glomerata_Arginine–vasopressin-like peptide

MMHKILLIFFFINSSFACLITNCPRGGKRGESPLLSMPSLIKECSSCGPGDQGQCFGPHICCGATIGCYIRSPETYKCRKESLHSHPCVSGFAMCRDNTARCAANGICCSQESCFLDSSCKIGDEYLSDRKIDQEFPRLIGENDQMIN

>Cotesia rubecula_Arginine–vasopressin-like peptide

MMHKILLVFFFINSSFACLITNCPRGGKRGESPLLSMPSLIKECSSCGPGDQGQCFGPHICCGATIGCYIRSSETYKCRKESLHSHPCVSGFAMCRDNTARCAANGICCSQESCFLDSSCKIGDEYLSDRKIHQEFPRLIGENDQMIN

>Cotesia vestalis_Arginine–vasopressin-like peptide

MMHKFLLVFFFITSSFACLITNCPRGGKRGESPLLSMPSLIKECSSCGPGDQGQCFGPHICCGASIGCYIRSPETYKCRKESLHSHPCVSGFAMCRDNTARCAANGICCSQESCFLDSSCKIGDEFLSDRKIDQEFSRLIGENDQMIN

>Diachasma alloeum_Arginine–vasopressin-like peptide

MSKIILIISALVCVSCACLITNCPRGGKRGENPLLSLHSLAAECPSCGPGHQGQCFGPNICCGTAIGCFIGTPETYKCRTESLYSRPCIAGFAMCRDNTGRCAANGICCSQENCHVDSSCRISDEFNDRKIGQEYSRLSPENEQ

>Fopius arisanus_Arginine–vasopressin-like peptide

MSKIILIMSALICMSSACLITNCPRGGKRGENPLLSLHSLAAECPSCGPGHQGQCFGPNICCGTTIGCFIGTPETYKCRTESLYSRPCIAGFAMCRDNTGRCAANGICCSQENCHADSSCRVGDEFSDRKIVQEYSRLSPENE

>Megastigmus spermotrophus_Arginine–vasopressin-like peptide

MSKVIIVLVTLVSLSCGCLITNCPRGGKRGGPTFLLENLARECPACGHEEQGRCFGPHICCSPSMGCLIGTPETLHCRKESLYSRPCVAGFAMCQGNSGRCAANGICCSQESCFIDSACKLVDETGNDRKIGAEFGAFLLENTGTNEHIL

>Microplitis demolitor_Arginine–vasopressin-like peptide

MNLPGTMIRKIFAVVFLLSTSFACLITNCPRGGKRGESPLLSMPSLIKECSSCGPNDQGQCFGPHICCGATIGCYIKGPETYKCRKESLYSHPCVSGFAMCRDNTARCAANGICCSQESCHVDPSCRVGDEYLSERKISQEFSRLIADNDQLIN

>Nasonia giraulti_Arginine–vasopressin-like peptide

MSKVIIVLTTLVSLSYGCLITNCPRGGKRGDPTFLLENIARECPACGREEQGRCFGPHICCSPSMGCLIGTPETLRCRKESLYSRPCVAGFAMCQGNSGRCAANGICCSQESCFIDSACKLVDETGNDRKIGAEFGAFLLENAGTNEHIL

>Nasonia vitripennis_Arginine–vasopressin-like peptide

MSKVIIVLTTLVALSYGCLITNCPRGGKRGDPTFLLENIARECPACGREEQGRCFGPHICCSPSMGCLIGTPETLRCRKESLYSRPCVAGFAMCQGNSGRCAANGICCSQESCFIDSACKLVDETGNDRKIGAEFGAFLLENAGTNEHIL

>Trichogramma pretiosum_Arginine–vasopressin-like peptide

MLKVIVFVFVMLFSLSHCCLITNCPRGGKRGDPTFFLENIARECPVCGHEEQGHCFGPHICCSPSMGCLIGTSETLSCRKESLYSRPCVAGFAMCQNNNGRCAANGICCSQESCFIDPACKLVDENNDRKIGAEFGAFLLENSGRNEHIL

>Telenomus podisi_Arginine–vasopressin-like peptide_partial

MIAWVIVLVSIFSLSSACLITNCPRGGKRADQSLMLMETSIRECTSCGRDGSGRCFGPYICCGSTIGCFIGTPETYKCRKESLYSKPCIAGFAMCRGNKGRCAANGICCSQEACYAD...

>Argochrysis armilla_Bursicon-alpha

MPFWKSWQNVYSSTGSSSERSCRYLQRFPRWQTFRRKSLHTRGGELSPLPFALLAIFTICPLDARSTVHGFFDFINLPSCARYVQVFSSSTSKCAVVTGLLICLLYDNVKAILGVDECRAVPVFHVLKRDGCLPRTIPSFACRGRCSSYLFPQVSGSKFWQMERSCMCCQESGEREASVSLFCPNPKPGERKFLKVRSTFPVHQSICRIPREY

>Ceratosolen solmsi_Bursicon-alpha

MTFIHIYNFVVQRLFILLVYSLTNINAKVGVDDCQITPVIHVLQYSGCVPKPIPSFACIGQCSSYLQISGSKIWQMERSCMCCQESGEREASISLFCPKAKAGERKFRKVITKAALDCMCRPCTDIEEYAIIPQEIADLADNGPFTSAAHFRHTL

>Chrysis viridula_Bursicon-alpha_partial

...HPARGVFRYSARVRCFVGVTYFESAVITGLLVCLLYNNVKAILGVDECRAVPVFHVLKRDGCLPRTIPSFACRGRCSSYLFPQVSGSKFWQMERSCMCCQESGEREASVSLFCPNPKPGERKFLKVRSNFPTY

>Cotesia glomerata_Bursicon-alpha

MFTYTARKFGLLLLVISQSTVEALVGVDECQVTPVIHLLRYPGCLPKPIPSFACTGRCSSYLQVSGSKIWQMERSCMCCQESGEREATVALYCPEAKPGERTFRKITTKAPLECMCRPCTGVEEHSIIPQEIAGFDEDNILTSSAHFLRSSDID

>Cotesia vestalis_Bursicon-alpha

MYHLPFYFLLVNFLFFASFVLHRFITARKFGLLLLLISQSTVEALVGVDECQVTPVIHLLRYPGCLPKPIPSFACTGRCSSYLQVSGSKIWQMERSCMCCQESGEREATVALYCPQAKSGERTFRKITTKAPLECMCRPCTGVEEHSIIPQEIAGFDEDNILTSSAHFLRSSDID

>Nasonia vitripennis_Bursicon-alpha

MAHYRCKEYFFITNYFNISLGWFILFINGLSAIVHIDECEVTPVIHVLQYTGCVPKPIPSFACKGRCSSYLQVSGSKIWQMERSCMCCQESGEREASVSLFCPKTKAGDRKFRKVITKAPLDCMCRPCTSIEERIVIPQEIAGLSNNGPLSNSAHFRNTFKLT

>Argochrysis armilla_Bursicon-beta_partial

...ETDNNSTSVSDEEFEGQLIRTCSADISVTKCEGYCDSRVQPSVMSSTGFYKECYCCRESYLKKRSIILNECYDADGIKLTGEKGYMEISLEEPAECQCLKCSDFSR

>Ceratosolen solmsi_Bursicon-beta_partial

...MLNSEIHIIKDEYSESGKLEKTCNGDILVSKCEGYCNSQVQPSIVTSTGFLKECFCCREIYLKEVIILLDHCYDANGVRIHQENEATLEVKIRTPGGYYYIDKQLRAKKLLLERQCGIDSNTRRQVRAQKRGVSFGNLHLSSSSDAYEIKHQLAQQI

>Chrysis viridula_Bursicon-beta_partial

...YEEFEGRLIRTCSADISVTKCEGYCDSRVQPSVMSSTGFYKECYCCRESYLKKRSIILNECYDADGIKLSGEKGYMEISLEEPA

>Cotesia glomerata_Bursicon-beta

MLIYLLLLLSIFKIEALLQNEEDCETLFSEVHLTKDEYDNDGGLIRTCSDDVSVTKCEGYCNSQVQPSVISSTGFSKECYCCRESYLKEKVVKLNHCYNPDGEKLETDKLASMEIKLREPADCKCYKCTDFLR

>Cotesia rubecula_Bursicon-beta

MLIYLLLFLSIFKIEALLQNEEDCETLLSEVHLTKDEYDNDGRLIRTCSDDVSVTKCEGYCNSQVQPSVISSTGFSKECYCCRESYLKEKVVKLNHCYNPDGEKLESDKLESMEIKLREPADCKCYKCTDFLR

>Cotesia vestalis_Bursicon-beta

MLIYLLFFLSIFKIEALLQNEEDCETLLSEVHLTKDEYDNDGGLVRTCSDDVSVTKCEGYCNSQVQPSVISSTGFVKECYCCRESYLKEKVVKLNHCYNPDGEKLETDKFATMEIKLREPADCKCYKCT

>Diachasma alloeum_Bursicon-beta

MVLGTLISQKVDDTCETLMSDIHVTKEEFDEGGILLRTCSDDLSVTKCEGFCNSQVQPSIMTATGFLKDCYCCRESYLKERNIILHHCYSPDGIKLVESETATMEIKLREPADCECFKCGDFSR

>Fopius arisanus_Bursicon-beta

MNNRMLRIFIYSIIGTEMISGTLISQKIDDTCETLTSDIHITKEEFDEGGILLRTCSDDLSVTKCEGFCNSQVQPSIMTATGFLKDCYCCRESYLKERNVILHDCYSPDGIKLVEPTTATMEIKLREPADCKCFKCGVDECKMTKMLHVLKADGCLPKRIPSFICHGRCSSYIQVSGSRMWQMERSCMCCQESGEREATVSLFCPKEKRKFRKITTKAPLECMCRPCTGIEEYSVIPQEIAGFAEEGPLTSSAHFRRSSVL

>Leptopilina clavipes_Bursicon-beta

MIKIVMYILTITLFCKNSANSQLESEENCETLQSEIHIAKDEKDETGGLIRTCSTDILVTKCEGSCNSQVQPSVKTSTGFLKECFCCRESYLKERLILLDHCYDVDGVRLEGEILGTMEIKLREPAECKCTKCGGFSR

>Leptopilina heterotoma_Bursicon-beta

MIKIIMYILTITSICKICVNSQIESEENCETLQSEIHIAKDEKDETGGLIRTCSTDILVTKCEGSCNSQVQPSVKTSTGFLKECFCCRESYLKERLILLDHCYDVDGVRLEGEILGTMEIKLREPAECKCTKCGGFSR

>Microplitis demolitor_Bursicon-beta

MMIILFFILLISGIDGIVNSDEDCETLMSEVHITKDEYDNSGGLIRTCSDDVSVTKCEGYCNSQIQPSVISSTGFVKECYCCRESYFKEKVVKLNHCYNSDGEKMETEKYATMEIKLREPHDCKCYKCTDFLR

>Nasonia vitripennis_Bursicon-beta

MRLIVSLLIAMFLKKIVLANALVNENCEKLDSEIRITKDEYSETGKLFRTCSDEIQVSKCEGYCDSQVQPSIVTSTGFTKECSCCREEFLEERTVYLNNCYDSNGKQFSLLDNSNMAIKIREPTNCKCIKCGFL

>Orussus abietinus_Bursicon-beta_partial

...VENVIKRFLITDEYDETGILRRTCSGDLAVTKCEGFCNSQVQPSVVTATGFLKECYCCRESYLKERLVLLTHCYNADGIRLESEEFGTMEIKLREPAECRCFKCGDFSR

>Psyttalia lounsburyi_Bursicon-beta

MHRIMFRMLLLSSIGSRMVLGSLISQKVDDNCETLMSEIHITKEEFDEGGILLRTCSDDLSVTKCEGFCQSQVQPSIMTATGFLKDCYCCRESYLKERNVILHHCYNPDGIKLMKPEAANMEIKLREPADCKCFKCGDFSR

>Telenomus podisi_Bursicon-beta_partial

...MKDEYDENGQMIRTCSGDVLVTKCEGFCNSQVQPSVVAITGFLKECYCCREDYLKDRLVKLDHCYNSDGVKFVEENLRAMEIKLREPAECKCH

>Argochrysis armilla_Cardioacceleratory peptide 2b

MSQRLFTVLVVLIFAISLNRGHKVNDRRTAGLVPYPRIGRNNEMTNFPRLDRASGLLHYPRVGRSDPPAPSYNRYYDPELDGNIDFYSAHEIDLDALDKDYTGFYLGKPIFLRGLDKVSKESLLPLLEHLHDVREFRPVQKSDDPRVIYSGLVGSRSSQGQLTLGGYTPRLGRENDGDVTSNVL

>Chrysis viridula_Cardioacceleratory peptide 2b

MSHRLFPLLVVLIFAISLNKGHKVTERRTAGLVPYPRIGRNNEMTNFPRLDRASGLLHYPRVGRSDPPALSYNRYHDPELDGNIDFYAAHDVDLDSLDKDYAGFYLGKPIFVRGVDRISKENLLPTFEHLHDVRDFRPVQKSDDPRFIYAGLAGSRSPQGQVTIGGYTPRLGRESDRDVTNVL

>Diachasma alloeum_Cardioacceleratory peptide 2b V1

MKDHPRFTVLIFLVFATSFNRGERINDLIAKFNERRAAGILAQPRIGRSAGMRIFGRPDGFAGLMQYPRVGRAGPPALADDMKYPEFDGDVKNHREGLDSPVDVDDQKNFDDDFLRRLGNGIWLDNPRYGPRKNQQRSQCENEMDSAGIFIVTPEDYQKMERQLLHYYPRSSEDHSR

>Diachasma alloeum_Cardioacceleratory peptide 2b V2

MKDHPRFTVLIFLVFATSFNLEAKTVKSKLRLYRLNPALIRQLSGGERINDLIAKFNERRAAGILAQPRIGRSAGMRIFGRPDGFAGLMQYPRVGRAGPPALADDMKYPEFDGDVKNHREGLDSPVDVDDQKNFDDDFLRRLGNGIWLDNPRYGPRKNQQRSQCENEMDSAGIFIVTPEDYQKMERQLLHYYPRSSEDHSR

>Fopius arisanus_Cardioacceleratory peptide 2b

MKDHPRFTVLIFLVFATSFNRGERISDLIAKFNERRAAGILAQPRIGRNAGMGSFGRADGAAGLVQYPRVGRSGPPTADDIKYPEFDGEVKNHRQLLDSVGDINDQKNFDEELRRRLGNGIWVESPKYGERRNLQRTQCENQMNSAGIFIVTPKDYQKIERQLLQYYPRLSDHSR

>Megastigmus spermotrophus_Cardioacceleratory peptide 2b

MRTTMEKTFYVLCLLSVFICVHLDGVVAKVLKKEAKEFHGDCPSGVPPSVCHRRLSLIPFPRVGRRIDPSFPRSQRTIGLVSQPRIGRSDPLMQMSEPTFARTHEETGWLESDPDYGHSTAISLYNQHRDFKNQIMSGEKEENLRNLENYLSAQRRESNSPWAFFLITPNFYTGDQPPQMDNLMPSQYGQDINDLNGE

>Pteromalus puparum_Cardioacceleratory peptide 2b

MCKSVSSLCLLLALLYIRFEGTAAKESVSFITDTRIPFDCPKGTPPSVCFKRWTMFPFPRVGRDSQARQQRSFGLVKYPRVGRSDPQLLLDKDDETWPGAHLDFTNDAYSRRVSEGRNRRDGPWAFFLITPNFREQPTAVDGMDSQFGADEVAME

>Argochrysis armilla_Crustacean cardioactive peptide

MKISVSVFALACCPLIFVCLPDFAAPQEIEEKIIPRNVDALMTDELAKMKRPFCNAFTGCGKKRSFYGKVGESQYYGSKEELRPLYKALVRAASQKAGGINANTDNTEGYRLVAGL

>Ceratosolen solmsi_Crustacean cardioactive peptide

MKVLQSTLLLLIIINFIKISHSDDGLDLIAMKRPFCNAFTGCGRKRDPAYAGPGYEGLQLPVPVYRALLRVAADAEARMRLRHAQAEPRVY

>Chrysis viridula_Crustacean cardioactive peptide

MKLSIFVCCALIFACVPDFAKPQEIEEKIIPRDVEALMTDDLTKVKRPFCNAFTGCGKKRSFYGKLPDLQYYDNKEDLRPLYKALVKAANQNAGLNENTNGYRIPVNVQDYDSYFDQKPA

>Copidosoma floridanum_Crustacean cardioactive peptide

MKILQLILSLMIIVGDLYDVSRADESAAPVLMKRPFCNAFTGCGKKRDSSYSAPAGGYNGFNDINNDYLQRTIPAYRVLLRIAAAARNNKNNAPLPTDIRKTKPLHNNSITTYAATSGTTFLLIDFE

>Cotesia glomerata_Crustacean cardioactive peptide

MKIIKYFNWILVGLFFINIVDAVISDENNKEYESLTINERNSIKLPGLKRPFCNAFTGCGKKRNYIDSSYDTTSAKNDVNDSTINIPLKLYQILMNNLNLKIQQVAKHKNYRDSINNNDEDYIYSTIHKQPIRKRYNL

>Cotesia rubecula_Crustacean cardioactive peptide

MKIIKYFNWILVGLFFIINIVDAVISDENNKEYESLTINERDSMKLPGLKRPFCNAFTGCGKKRNYIDSSYDSTKNDVNDSTINIPLKLYKILMNNLNLIIQQVDKHKNYRDSINNNDEDYFYSLIHKQPIRKRYN

>Cotesia vestalis_Crustacean cardioactive peptide

MKIIKYFNWILVGLFFINIVNAVISDENNKEYESLTINERDSIKLPGLKRPFCNAFTGCGKKRNSIDSSYDITNNDVNDSTINIPVKLYEILMNNLNLKIQKIAKHQNYRDSINNNDDDYFYSIHKQPMRKRYNL

>Diachasma alloeum_Crustacean cardioactive peptide

MKVGYCVKWTLAGLLVVSLASAIETDDKDEQQIFNSGVEELAKSKRPFCNAFTGCGKKRNYYDNSLDRPEDSGKITIPIPIYKALVRAASQELRNAIERGESPDESYQDYLPLLTLKRPIRSRFQS

>Fopius arisanus_Crustacean cardioactive peptide

MKIECCIKWTLAGLLLAALSSAIETDDKDDQQIFNSALEELAKSKRPFCNAFTGCGKKRNYYGNSMERSGDLPKITIPIPIYKALIRAASQELRNALERGESIDESYQDYLPLLTSKRPIRGRLQS

>Leptopilina clavipes_Crustacean cardioactive peptide_partial

...KQKRPFCNAFTGCGRKRITIPSYNIHIINNNGVLFNTILLLHILLSRM

>Megastigmus spermotrophus_Crustacean cardioactive peptide

MKVLQFILPLLIIADFMDIVRADDGLDMVAMKRPFCNAFTGCGRKRDPAYAAAAAAAGSYGSVQLPSSVYRELLRILAARNGAAEVRMRKRLARGSFESQEQLPRAAALF

>Microplitis demolitor_Crustacean cardioactive peptide_partial

...MIDGDSIKLLGLKRPFCNAFTGCGKKRNYNEPKEVYGGEDDINNNNDNNDSINDSIVKIPLKLYKVLINSFNRKIHQIVQHKQALNNNLITTKQNNNVNDEDYYFSSLQKQPIQKHYNL

>Nasonia giraulti_Crustacean cardioactive peptide

MKVLKLVLSLLIIIESVEIARADDGLEIVAMKRPFCNAFTGCGRKRDPSYASGLQLPIPLYRALLRLSSLRNGAAAEVRMSQRPLGLIGPENLVQLGPQAPRLMAPVLVPSLY

>Nasonia vitripennis_Crustacean cardioactive peptide

MPDGSIKEQVLKLVLSLLIIIESVEIARADDGLEIVAMKRPFCNAFTGCGRKRDPSYASGLQLPIPLYRALLRLSSLRNGAAAEVRMRQRPLGLIGPENLVQLGPQAPRLMAAPVVVPSLY

>Orussus abietinus_Crustacean cardioactive peptide

MKVNGYVYCALVFVSLQVMSNSASLRSRVDPQFLENIEPDQSNKMKRPFCNAFTGCGRKRSSVEYNLGMQGDNSQSIRLSVPLFKALLRATSPEIRENLDCQSDDCNVQEVSQDISSLARKMINRDRYDS

>Pteromalus puparum_Crustacean cardioactive peptide_partial

...MKRPFCNAFTGCGRKRDPSYASGLQLPIPLYRALLRLSSLRNGAAAEVRMRQRPLGLIGPENLLQLGPQSPRLAP

>Argochrysis armilla_CCHamide-1

MASTSKTTCITSVVAWFSTFLLIFYFVGYASGSCAQYGHSCWGGHGRKSFDNSGNNEPRFGLSNAAGREQWILSRLGMNQVTPTVTEKHHVRWNGIYEPKTFRTRRFNPVQGPTSLINNDGLSNDKNNDETDTIAEEILLIPQKASRNEIRQPSKLQLLKILSNDEKNLK

>Ceratosolen solmsi_CCHamide-1

MRSNAELIILSKIYCFIAVLVLISSFAGCAAGSCAQYGHSCWGGHGRKNDPESDPGNRRIGPSGLASRWLFFSRMVNRQAALSPRSRPRMMPEMSLLTEPRLR

>Diadromus collaris_CCHamide-1

MGAYLLTCFSGSCLSYGHSCWGAHGRKSNNIVHRLTNAHRDSGVVAEMETEENAPAPRNNNRWILSRLIRGRLTVPASVSTAGKRSGNRWYEARKIIALPENWDIQYDSTRTQLLRDRNAEDAESIENGKNNDDSVDIPQKLLEPHAETRELLLLPSGDETDNRSSRTHGDPVVDALASLLSD

>Megastigmus spermotrophus_CCHamide-1

MRGNPELVVLRNIYCLAAVSILVSSFAGCAAGSCLSYGHSCWGAHGRKSGAVRPIMENLVSDSERRIAPASLSYGERWLLSRLVSRQLIQTPWAAKLRARMMVEQPLALIPQSLDEESDVAAAAAAEASGIKHSATMDKLSEYADVRDLAKGEPRMREGESDIYMLPPRKDFTDDNDQSIKVGLFKILKNIDEKLK

>Nasonia giraulti_CCHamide-1

MKMRGNPELVAPRNVYCLAGVLILISSFAGCAAGSCLSYGHSCWGAHGRKSGVVRPVDSLMPSDLSLKHPAAGQRVVSPSFGERWLLSRLVNRQLMQVPLRGRMLTQPLGNELPEVTSLAKHSLPLDKLSGSVDVRDLAKEEQRISKGEESNILLYPQSKDFPNDDEHAIKMGFYKLLKNFDEKLK

>Nasonia vitripennis_CCHamide-1

MRGNPELVAPRNVYCWAGILILISSFAGCAAGSCLSYGHSCWGAHGRKSGVVRPVDSLMPSDLSLKHPAAGQRVVSPSFGERWLLSRLVNRQLMQVPLRGRMLTQPLGDELPEVTSLAKHRCVFAFLYTYYPAIS

>Pteromalus puparum_CCHamide-1

MKMRGNPELVALRNVYCLVGVSILISSFAGCAAGSCLSYGHSCWGAHGRKSGVIRPVDSLMPNDLSLKHPAAGQRVVSPSFGERWLLSRLVNRQLMQVPLRGRMLTQSLGDELPEVASLAKHSSSLDKLSGTVDVRDMSKEEQRISDGEESNILLYPQSRDFPNNDERAIKMGFYKLLKNFDEKLK

>Telenomus podisi_CCHamide-1

MLAWLSVTKCNALSKITRIVMIILVCHLAGCAAGSCISYGHSCWGAHGRKNGDSQNLAKPMLEARAYQRPPNIYEMIFSRLENRRGNEARLSPIQVHNFEHKEKNWKRNDFIRTRDKDGRDLKLLLTQL

>Argochrysis armilla_CCHamide-2

MIIQRIKNLWKSIMWFLVEKGGCSAFGHSCFGGHGRKFDPRTSLENRLQETQAISLYSSKQDNESPRPNNELHLGGQDFKMEDRQTRSNGNPRNAQRLDPYTLSFLVDQWVQFILFSLCLYTLEYA

>Ceratosolen solmsi_CCHamide-2

MTGSSSVSTTMFIALLLITCYALEIAAKRGCSSFGHSCYGGHGRKSSYPEALEFDVVRNDNDLEMNYDPDRSNAWNLALLPSQDYERHAQNTLNSMYEQALSWSIFIRKLERLFD

>Copidosoma floridanum_CCHamide-2

MKSARTSTTSSLLGLFVTILLVASFGVEISAKRGCSAFGHSCYGGHGRKSESENMMDADPKIKEDVQLDQNVAIQNGEREALTQILQLLNRQDERFVNSLRERLSPNSWSLMVRKLVALPRHQYPTYSDIRNPY

>Diadromus collaris_CCHamide-2

...DDGKENDGKEGDGKLEGEHRGGCAAYGHSCFGGHGRKFAPDEQDDRFKSVDADPGNSNDVVQYRADGLTRQQVEDFVESRNRALELSRRARDERKYMPILVKEWLTSYRLPYNADPERNEE

>Leptopilina clavipes_CCHamide-2

LAVILITAFLIISLSSETSAKRGCGAFGHSCYGGHGRKSDLNQNGIMSNERNIINRELPEYVHTNEGTFAIESLQKNQERAG

>Megastigmus spermotrophus_CCHamide-2

MKGSPSTTSAFILTVLLVASCSVEISAKRGCSAFGHSCYGGHGRKSYPETAENEVARNKEEAQLDYDLARPNAESVALVPSQGYNRQEQRTANSMHDRPSSNSWSLFIRKLLVPFRRPYMAYSEAQK

>Nasonia giraulti_CCHamide-2

MGSTSTSSALFLTFLLIASFSVEISAKRGCSAFGHSCYGGHGRKSYSESVDLDLARNKEETQQVDYDLSRSNVESVALVPSQGYNRQEQHSIHSIHERPNTNSWSLFIRKLLVPYRRPYMAYSESQK

>Nasonia vitripennis_CCHamide-2

...GCSAFGHSCYGGHGKRSYSESVDLDLARNKEGTQQNDYDLSRSNAESVALVPSQGYNRQEQRSIHSIHERPNTNSWSLFIRKLVNYFKFL

>Pteromalus puparum_CCHamide-2

MGSTSTSSALFLTFLLVASFSVEISAKRGCSAFGHSCYGGHGRKSYSESVDLDAARNKEETQQVDYDLSRSNAESVALVPSQGYNRQEQRSIHSIHERPNTNAWSLFIRKLLVPYRRPYMAYSEAQK

>Telenomus podisi_CCHamide-2

MKIIPGWSNVTSLALFHVFLIVLLTFGVQVSSKRGCSSYGHSCFGGHGRKSQDNGIDFFGLKYINPATKLSSKDAINLETTGPYQVQESYKPFSKNLQILLRKLVSPSKDKLFVRN

>Trichogramma pretiosum_CCHamide-2

MGSLPTRSAFVVTVFLFTLCCVEISAKRGCSAFGHSCYGGHGRKSNIELTDFNEKNKDEVQEEYDLAKPNLENVAYVPSQGFTRDGFRLDNRAIDRLPLRLNPNSWSLFIKKLMTTNRHPYMTYGEVQK

>Argochrysis armilla_CNMamide

MTRRRCPKGTSLCLKALAMCYLFAGSNIVFVLAEAEPLPPAMIYGDNLTEDAENLMLVNRLKQVAERKQDIIEQERELTEEQIAIQALLEAKAGNDGQPLPYPPKMTYQGMSGPPASRAKPASYMSLCYFKICNMGRKRQM

>Chrysis viridula_CNMamide

MTRSRCPKGTSLCLKALAMCYLFAASNIVFVLAEAEPLPPAMIYGDNLTEDAENLMLLNRLKQVTERKQEIIEQERELSEEQVAIQALLEAKAGSDGQPLPYPPKITNQGMSGPPASRTRPSSYMSLCYFKICNMGRKRQM

>Cotesia glomerata_CNMamide

MKQSRGKSYWRFLGILLFSIAALETVWAASVPEPGMVYSDPFPDDTENYIWLQKLKQIHDIKLEISEDERELSDAQLEIQAMLEANARNQRVPHYPIPPEFLSEDPEALPVPEPRVNPGPTHSGKRTSYMALCHFKICNMGRKRQLPEKK

>Cotesia rubecula_CNMamide

MIFRIMKPSRGKSCGRFLGILLFSIAALETVWAAPVPEPGMVYSDPFPDDTENYVWLQKLKQIHDIKLEISEDERELSDAQLEIQAMLEANARNQRVPHYPIPPEFLSEDPEALPVPEPRVNPGPTHSGKRTSYMALCHFKICNMGRKRQLSEKK

>Cotesia vestalis_CNMamide

MISQIMKHSRGKSCWWFLGILLFSIAALETVWAAPVPEPGMVYSDPFPDDTANYVWLQKLKQIHDIKLEISEDERELSDAQLEIQAMLEANARNQRVPHYPIPPEFLSEDPEALPVPEPRVNPGPTHSGKRTSYMALCHFKICNMGRKRQLPEKK

>Diachasma alloeum_CNMamide

MKQKIMKRKCTSYTSRFWILALVSLTTIHNVWTAPQPLSAMYYSDNSVEDSENLLLLQRLKQVAALKHEILEEERELTEAELEVQAIIEAKLRNQRILQPIESESSNEEAEMLPIPSAVVHHAPVGHTGKRTSYMGLCHFKICNMGRKRQL

>Fopius arisanus_CNMamide

MKQRIMRKKCTSLRLLILAVFSLTTIHNVWGAHQPLSAMYYADSLVEDSENLLLLQRLKQIAALEHKILEEERELTEAEFDLRAIFEEKMRNRRPIGSESFDEEPEKLPIPSAVVHAPSGHTGKRTSYMGLCHFKICNMGRKRQ

>Ganaspis sp._CNMamide

MKGRIHENRTGVCLLTLLVSCLIHAVIADPEPAQSGLVYADIEDPEEMVLLQRLRSIVRHKQETIEQEKELTEEQIAIQAILDAKARSQRGQQTIPEEFSVEDTETLPVPSAIFNHGPQHPGGKRTSYMTLCHFKICNMGRKRQL

>Leptopilina boulardi_CNMamide

MKGRIYESRTGLCLLALFVSFLIDYHPVIAKPEPASFDIEDTEELVLLQRLRTIVRHKQEVIDQEKELTEEQIAIQAILDDKARNQKDHQTMLEEFSMEDTETLPVPSAIFNPPQHNGKRTYMTLCHFKICNMGRKRQI

>Leptopilina clavipes_CNMamide

...MLEEEFSMEDTETLPVPSAIFNPGPQHNGGKRTSYMTLCHFKICNMGRKRQL

>Leptopilina heterotoma_CNMamide

MKGRIYESRTGLCLLALFVSFLIDNHAVIAKPEPASYDIEDPEELVLLQRLRTIVRHKQEVIDQEKELTEEQIAIQAILDAKARSERGHETMLEEEFSMEDTETLPVPSAIFNPGPQHTGGKRTYMTLCHFKICNMGRKRQL

>Megastigmus spermotrophus_CNMamide

MPGSAYKPRTGLLFLALSVVFCVFGSHLVDAAPQPIPPGMMYREDYGVDIPSQSLVERLKQVVALKEEIMEKEINELEEEQMQIQELIEAKARMQREQQQQQQHPQEFSMEDSEQLPVPAAVYHSGPPHSGKRTSYMALCHFKICNMGRKRQM

>Nasonia giraulti_CNMamide

MRGNSYKLSTGLCVLAVSMLMLGCHKVDAAPQPIPPGMMYREDYGAELPSQTLVDRLKQIVERKEQKIEKEIAELAEEQMEIQALIDAKARNQREHQQPPEFSEEDTEALPVPAAMYHSGPLHSGKRTSYMALCHFKICNMGRKRQM

>Nasonia vitripennis_CNMamide

MRGNNYKLSTGLCVLAVSMLMLGCYKVDAAPQPIPPGMMYREDYGAELPSQTLVDRLKQIVERKEQKIEKEIAELAEEQMEIQALIDAKARNQREHQQPPEFSEEDTEALPVPAAMYHSGPLHSGKRTSYMALCHFKICNMGRKRQM

>Orussus abietinus_CNMamide

MTGRIRDIGTGVCVLALLLSCVLAEPEPEPEPAGPIFGDDFDEDAESQLLLKRLKQIAERKQEAFEQERELTEEQIAIQEILEEKMRSQRKYAKDDAEVLPVPSAVIHAPIRGEKRTSYMTLCHFKICNMGRKRQM

>Pteromalus puparum_CNMamide

MRGKSYALSTGLCILAVSMLIFGCHKVDAAPQPIPPGMMYREDYGAELPSQTLVDRLKQIVERKEQKIEKEIAELAEEQMEIQALIDAKARNQREHPQPPEFSEEDTEALPVPAAMYHSGPLHSGKRTSYMALCHFKICNMGRKRQM

>Telenomus podisi_CNMamide

MTGRIFTLLTGFNILVICLFCVLTDCVSARPQPTREDPSFNKGDYLLLNLLKQISDSKHEMAEQERELGEEQMEIQAYLARKASMRREEEVQEEMSEESPEALPVPSAIIHNQPLHNGKRTNYMALCHFKICNMGRKRKL

>Argochrysis armilla_Corazonin

MAVCKISTFLLLSLVVTTAVCQTFQYSHGWTNGKRSRTSGLVAIAPEFKATSSYNQNTDPELSNIWLQCALSKLKMLLQESGDDQLYLLPCELIDSPKKTYSKI

>Cotesia glomerata_Corazonin

MLYLSWTPVILSVVIITATCQTFQYSRGWTNGKRDIAPIGFNIQDFKPDNFETNSIISNDNLHNNDGTVQCNLRKIKVILQGDRREQIYLCRLLEILSKINKEDKLPIHYRHPIFEANDINN

>Cotesia rubecula_Corazonin

MLYLSWTPVILSVVIITATCQTFQYSRGWTNGKRDIAPIGFNIQDFTPDNFETNSIISNDNLHNNDGTVQCNLRKIKVLLQGDRREQIYLCRLLEIFSKINKEDKLPIHYRHPIFEANDIDN

>Diachasma alloeum_Corazonin

MANAMTVLVILLVTMTVVTYGQMVQYSRGWRGGKRADLGAFGGVGGLPSEFKMNFPAQGAIRRTNAPDTIHCGLRKLRMLLRGNTNDQLYHLPCELLNILPREVEGNQHVQHENSHHLDLYDDNNNNNNNNNYDDNN

>Fopius arisanus_Corazonin

MTRALIILMILVGTISVITGQTFQYSRGWTTGKRADSGVLGVGGDLPSDLKMNFPTQGIIRRTNDPENIHCGLRKLKMLLRGSTNNQLYHLPCDLLNILHREVEAGSERVGHHKNSHHLDLYDNDNDNVNDNDNYLNNK

>Megastigmus spermotrophus_Corazonin

MVVTRILLFFVFSLTMTTVICQTFQYSHGWTNGKRSVSSMLEELVNSPSKNAAQLDNVLVNCELQKLRLLLQGNINSQVLQHVCDFYDSPKRNIPEIVVNEHFRRQSSSTSNNY'

>Microplitis demolitor_Corazonin

MRYLSYTPVILSLVLITATCQTFQYSRGWTNGKRDNSANGFTLPDFRIDNFDTNNLSNNNDGSNVQCNLRKIKMLIQGDHHEQIYLCKLLDLISKINREEKLSSNSRHPAFENSDIDN

>Nasonia giraulti_Corazonin

MIRGLTIALVTLALVSLATCQTFQYSRGWTNGKRAETSPLASILDYRAAMAGAGGRMNDLQMARCANLHKWKMFLLRSDDNEEVCRVPCEFLDVLRQCLIRQDKASPNDLSDFRRPSAPALESSSSLSY

>Nasonia vitripennis_Corazonin

MIRGLTIALVTVALVSLATCQTFQYSRGWTNGKRAETSPLASILDYRAAMAGAGGRMNDLQMARCANLLKWKMFLLSSGDNEEVCRVPCEFLDVLRQCLIRQDKASPNDLSDFRRPSAPALETSSSLSY

>Pteromalus puparum_Corazonin

MIRGLMIALLTVTLVSLATCQTFQYSRGWTNGKRAETSPLASILDFRAAMTGAAERMNDLQARCANLQKWKMFLLRSN

>Argochrysis armilla_Diuretic hormone 31

MQREIALSCLALTVLVVFAVSSSTVDAAPYTHQSYWDQLEQEDPETLGDMFTRLERALMLENNKRGLDLGLSRGFSGSQAAKHLMGLAAANYAGGPGRRRRSEQA

>Ceratosolen solmsi_Diuretic hormone 31

MQKKIALSWLLLATLAALTIPNRVEALPYTRSRTLWNQFQEDYPEAAQELLDRIENLAIFVQPDNAKRGLDLGLSRGFSGSQAAKHLMGLAAANYAGGPGRRRRSEQA

>Chrysis viridula_Diuretic hormone 31

MQREIALSCLALTVLVVFAVSSPTVDAAPYTHQSYWDQLEQEDPETLGDMFTRLERALMLENNKRGLDLGLSRGFSGSQAAKHLMGLAAANYAGGPGRRRRSEQA

>Copidosoma floridanum_Diuretic hormone 31

MQKKIALPRLLLAAVVLAAFAASYPTQALPYRLAYDERRAAWNPYQMDYPDATQELLERLESMGLIRAPEDQKRSLDLGLNRGYSGSQAAKHLMGLAAANYAGGPGRRRRSESAA

>Diadromus collaris_Diuretic hormone 31

MQRGIIAWMTLATVAFVTVVATVEAVPYPRPIYWDQLEEEDPEVIMEWLNRIGSFVRVHDLENTKRGLDLGLSRGFSGSQSAKHLMGLAAANYAGGPGRRRRSEQA

>Diachasma alloeum_Diuretic hormone 31

MQKGMAMTWTILAVLISVGILTAVEAAPYPLERALYLDQLQEDPEKMYEVLTKLNRYLQQQDNEKRGGFGLDFGLNRGFSGAQAAKHLMGMAAANYAGGPGRRRRSDQA

>Fopius arisanus_Diuretic hormone 31

MHKRMAMICTALAALAALGILTTAEAAPYPQERALYLDQLEEDPEKMYEVLTKLNRYLQQQDNEKRGGFGLDFGLNRGFSGAQAAKHLMGMAAANYAGGPGRRRRSDQA

>Ganaspis sp._Diuretic hormone 31

...MNPRVAKENFDWMENAGQELENVKRGLDFGLNRGLSGTQVGKHLIGLTAANFANGPGRRRRSE

>Leptopilina boulardi_Diuretic hormone 31

...MNPRMTKENFIWMENGQESENVKRGLDFGLNRGLSGTQVGKHLIGLTTANFANGPGRRRRSQ

>Leptopilina clavipes_Diuretic hormone 31

MLAMELKLKWMKIIFTFILLATRSFSLITATNKEFRDTAQPTWQTIRNILQYIPRIERENFEQIENMEVQDLEKAQPMWQRILEMNPRIAKENFIWMENGQETENVKRGLDFGLNRGLSGTQAGKHLIGLTTANFANGPGRRRRSQ

>Leptopilina heterotoma_Diuretic hormone 31

MPVMELTLNWMKIFLTFILLASRSFSLITATNNEFRDTAQPTWQTIRNILQYIPRTERENLERIENLEVQDLEKAQPIWQRILEMNPRIGKENYIWMENGQETENVKRGLDFGLNRGLSGTQAGKHLIGLTTANFANGPGRRRRSQ

>Megastigmus spermotrophus_Diuretic hormone 31

MQREIAFSWLLLAAFAVIAVSNRAEAIPYIRSHSLWNQFQEDYPEAAQELLDRIENLAVFVQPDNTKRGLDLGLNRGFSGSQAAKHLMGLAAANFAGGPGRRRRSEQAA

>Microplitis demolitor_Diuretic hormone 31

MHEFKQLKLSTLVFMLSPHFPERQLYLEQLEDDPQAFYELIAQYYKLKELENKRAYGLDFGLSRGFSGSQTAKHLMGMAAANYAGGPGRRRRSEQA

>Nasonia giraulti_Diuretic hormone 31

MQREIALSWLLLATFAALAISNRVEALPFIRSRSLWNQFQEDYPEAAQELLDRIENFAVYAQPDNAKRGLDLGLNRGFSGSQAAKHLMGLAAANYAGGPGRRRRSEQA

>Nasonia vitripennis_Diuretic hormone 31

MQREIALSWLLLATFAALAISNRVEALPFIRSRSLWNQFQEDYPEAAQELLDRIENFAVYAQPDNAKRGLDLGLNRGFSGSQAAKHLMGLAAANYAGGPGRRRRSEQA

>Orussus abietinus_Diuretic hormone 31

...IVFYIPSTKRGLDLGLSRGFSGSQAAKHLMGLAAANYAGGPGRRRRSEQA

>Pteromalus puparum_Diuretic hormone 31

MQREIALSWLLLATFAVLAISNRAEALPFIRSRSLWNQFQEDYPEAAQELLERIENFAVYAQPDNAKRGLDLGLNRGFSGSQAAKHLMGLAAANYAGGPGRRRRSEQA

>Trichogramma pretiosum_Diuretic hormone 31

MQHKIVLCCLVLLVATALTVQALPYTRARLMWNQFQEDFPEAAQELEDRIENMALFVQDNNAKRGLDLGLNRGFSGSQAAKHLMGLAAANYAGGPGRRRRSEQASRAASVAAQVVVPGNEQV

>Ceratosolen solmsi_Diuretic hormone 43

MSLVSLLATTTLITLANSTPMIYTTNQRAKIPVDYDHLLLMLLLNHQDFGQKTKPLEPRTLESLYGREKRIGSLSIVNSVDVLRERVLLELARRKAMEDQRQISENRRLLDSVGKRSGPGPRELGYRSPGDPRKTSPGSGCEDEGRERLDRTMAQEFLVGRV

>Copidosoma floridanum_Diuretic hormone 43

MSLAGVLATSLIVGLVVQGASCAPAEQRASIPIDRDHFLLVMLLNRLDFAPKAKGLDEARYGEVSGYGEEGGPATSRDKRIGSLSIVNSVDVLRQRVLLELARRQAMEDQRQISENRRLLDSMGKRSGHEYHRDGAADDGKLMEEQQVPRDDRSRQLERLLVQ

>Cotesia vestalis_Diuretic hormone 43

MFLMSLLTVTTLITLSKSTPLSYLTHQRRDAPTDGGGDDDDGVFVPYIIDERGFSANEFYWPQDENLKVNDSVSGIDRGKRIHSLSVTNSLDVLRDRVLLELARRKAQQEKIQIDANRHYLDNIGKRSNQYIKMDLPQIFEPKNIKIPRIFDHQLIGDQSEFNYYTAKNPLPLDST

>Diachasma alloeum_Diuretic hormone 43

MILSSLLIVAVLMNLTKSASSRISPTEEIYVEIPYIAATWDSHEYYEDSSQNEQTGIPRKEHSYSSNVRRKKISSLSITNPMDVLRQRFILELARRRQMQQQEQAKANREILNDIGKRSTGYFGLVCPSIKTKNRQVLREPINQMYSNNPDWSHRDRVDWYYTEHFKELNTGFYQLNEVSFDSIILSFTNCYD

>Fopius arisanus_Diuretic hormone 43

MKKRILTGLFIAAMIMNLTKSTSYRKRATYEILAEMLYIAKTWDSKEYHSDYAANKQTRYPNIDNTDSSNVRRKKISSLSITNPMDVLRQRFILELARRRQMQQQEQAKANREILNDIGKRSAEFDIISPSILMNSEEVECQYTHKVFSDNPDYSYIKSLAGYDAENSRN

>Ganaspis sp._Diuretic hormone 43

MMILIGVIATTTLISFAYCTPLSYTTFRRRDLPVLEHPELVLLFDPRDSVLENEMFGSGNDPRSTVTRSKRIGSLSINNSVDVLRQRVLLELARRKAMQDQYQIDENRRMMQTIGKRSSSDFVTRYPTGANKPSNSGIYVIEDRPNDRDPSKSPNRISDGTPVWLEGNDHEQDQAQRVQTNEVHLL

>Leptopilina clavipes_Diuretic hormone 43

MMILIGVIATTTLISFTRCTPLSYTSFRRRDLPVLDRPELVLLFDTRDSILENEMFGSGNDPRSTVTRSKRIGSLSINNSVDVLRQRVLLELARRKAMQDQYQIDENRRMMQTIGKRSSSDFVTRYPNSANNKPSNSGIYVIEERPNDRDPNKSNVRISDRTNVWLDGN

>Leptopilina heterotoma_Diuretic hormone 43

MMILIGVIATTTLISFTRCTPLSYTTFRRRDLPVLDRPELVLLFDTRDSILENEMFGSGNDPRSTVTRSKRIGSLSINNSVDVLRQRVLLELARRKAMQDQYQIDENRRMMQTIGKRSSSDFVTRYPNSANNKPSNSGIYVIEDRPNDRDPNKSNVRISDRTHVWLDGNDHDQDQEQRVQTNEVHLL

>Microplitis demolitor_Diuretic hormone 43

RRDVATDGDNGVYIPYLIDERGLSVNEFYWPQDENLKVNDSVSGIDRAKRIQSLSVTNSLDVLRQRVLLELARRKALQDQQQIDANRRYLDNIGKRSNQYLKMDYLPQMLETKNIARIFDHQFADQPEQNYYTAKNPLESTLDWYNNNNNNANDVTNQYNVNDQLRKIKKINKIRLL

>Nasonia giraulti_Diuretic hormone 43

MSLVSLLATTTLITLATSTPMILADDVAAAGPFGGAGLRAKIPVDHDHFLLIMLLNRRDFGVKSKPPLDETGGEYITSLDSDMSSSGGGGGFDTGGIAVSDLTRGKRIGSLSVVNSVDVLRERVLLELARRKAMENQQQLGENQYVFKSVGKRSGGLSYRDHRIARKKRLQYQLMNRQQQPERLHAQ

>Nasonia vitripennis_Diuretic hormone 43

MSLVSLLATTTLITLATSTPMILAEDVAAAGPFGGAGLRAKIPVDHDHFLLIMLLNRRDFGVKSKPPLDETGGEYITSLDSDMSSSGGGGGFDTGGIAVSDLTRGKRIGSLSVVNSVDVLRERVLLELARRKAMENQQQLGENQYVFKSVGKRSGGFSYRDHRIARKKRLQYQLMNRQQQPERLLAQ

>Orussus abietinus_Diuretic hormone 43

MMLIGFLATTAMISLARSSSISYSTFRRREVPMLDRPEVLLLFDRAPSLENEPFDSSNEPASSLVRPKRIGSLSIVNPLDVLRQRVLLELARRKMRQDQQQVDANRRFLETIGKRSVPSLEPKDFRDSQDLEEDPEVDSANDDDPRRSLPSRHPGEDRDGPVAPGRKTNRIQDWLDSDDDLRRSQDDRTRRMQLDELHLL

>Pteromalus puparum_Diuretic hormone 43

MSLVSLLATTTLITLATSTPMILADDVAAAGPVGGGSGQRAKIPVDHDHFLLIMLLNRRDFGLKTKPPLDETGGEYITSLDSDMSSGGGGGGFDTGGIAVSDVTRGKRIGSLSIVNSVDVLRERVLLELARRKAMEDQRQISENRRILDSVGKRGFSYRDHRIARKKRLQYQLNHQQQPERLLAQ

>Telenomus podisi_Diuretic hormone 43

MYKKRESYLDHSNLMSLLGQKDPRFENEMYDMDPGSSIRNKRIGSLSIINSVDVLRDRVLLELARRKALQNQLQIAENRRFLDTIGKRSIPDYEESYAKYIGRANESDASRQEKANPDETAMTERTRVWIVEDDPAVLHNNVEQVQRLQKSEQHLL

>Trichogramma pretiosum_Diuretic hormone 43

MGKQQRRCSMMSLLSLLATTTLVGLAQAGPLARTSSHNAAIQSLQIPVDRDRFLLILLVNQKDFVAEGKSMEDMLEYLNEHDTGTTSNEADSNVSSSSSNGRDKRMGSLSIVNSVDVLRQRVLLELARRKAMEDQRQISENRRLLDSVGKRSSYPGSSIMARKKDSSAADSSLEHDQLQRQFDELMMQ

>Ceratosolen solmsi_Eclosion hormone

MIFTKRLFLTSLMIYVVFITIIVAHPSIGTCFRNCSQCKKFLGIYFDVQLCDEYCVKFKGKMTPDVEDPDSLAPFITNLNEVQ

>Chrysis viridula_Eclosion hormone

MAFSSRVLFTFFLALFLLFFIVSADMSDSKYRTGACIRNCAMCKKMYGPFFEGDSCAQFCLKYKGKYIPDCEDEDSIRQFVTGIDE

>Copidosoma floridanum_Eclosion hormone

MISTKRLFLILLLTCVVFIAVSTAHPSVGTCFRNCSQCKKFLGIYFDVQLCDEFCVKFKGKMTPDVEDADSLAPFITNLNEVQ

>Cotesia glomerata_Eclosion hormone

MKISNELCLLFIVGIITMVIAMPADGNRIGTCIRNCAQCKKMFGPYFEGQLCADTCVKFKGKMIPDCEDADSIAPFIVPLENE

>Cotesia rubecula_Eclosion hormone

MKISNELCLLFIVGIITMVIAMPADGNRIGTCIRNCAQCKKMFGPYFEGQLCADTCVKFKGKMIPDCEDADSIAPFIVPLENE

>Fopius arisanus_Eclosion hormone

MAPLHRLVSLLIVAIVVVHLTAPAGATKDRVGTCIRNCAQCKRMFGEYFEGQRCAESCLKFKGKSIPDCEDINTIEQFIHRVDEDED

>Leptopilina boulardi_Eclosion hormone

MKISAKILLICIIAFTLLILTTTASLAHVGTCLRNCAQCTKMLGPYFEGSLCADTCVKLKGKIIPDCEDVNSIEPFITRPDED

>Leptopilina clavipes_Eclosion hormone

MKILAKVFLICIIAFTSLILTTSASLAHVGTCLRNCAQCTKMLGPYFEGSLCADTCVKLKGKIIPDCEDVNSIEPFITR

>Microplitis demolitor_Eclosion hormone

MKISSHFVLLFIVGIITMVITMPADGNRIGTCIRNCAQCKKMFGPYFEGQLCADTCVKFKGKMIPDCEDADSIAPFIVPLENE

>Nasonia giraulti_Eclosion hormone

MVLSKRLLVVLLMSYLVFLTVAAAHPSVGTCFRNCSQSKKFLGIYFDVQLCDEYCVKFKGKMTPDVEDPDSLAPFITNLNEVQ

>Nasonia vitripennis_Eclosion hormone

MVLSKRLLVVLLMSYLVFLTVAAAHPSVGTCFRNCSQSKKFLGIYFDVQLCDEYCVKFKGKMTPDVEDPDSLAPFITNLNEVQ

>Pteromalus puparum_Eclosion hormone

MVLSKRLLVVLLMSYLVFLTVAAAHPSVGTCFRNCSQSKKFLGIYFDVQLCDEYCVKFKGKMTPDVEDPDSLAPFITNLNEVQ

>Argochrysis armilla_Elevenin

MFLQSCVIILGIFAWYAEIASGRMVDCERYPFHSTCRGTMSKKNGVRTSVFREQDCDDFNDDLPCTVEKLK

>Chrysis viridula_Elevenin

MFLQPCVIFLSIFAWYAQTASGRMVDCERYPFHSTCRGTMSKKNAVRPGIFSEQDCDDFNDDLPCTVEKLKSFEDRRRMDNFHNEYDAYPRSKLWISLLDKDLDTDALYDAFTSNYERHHRKRKNEEQQKFNERRIRFRQQPGFSVRTDALDNDY

>Cotesia glomerata_Elevenin

MMNFRWALLTLTIFAWWSHADAGRLARPDCEKFVFHPHCRGTQAKKRVIADPTSINSSMKDGNTEKLCICKYLTKSGKKELLAVTKLLETMLANGVDVNMLYDTYSNMGLLSKDQNNNLRSTHLTNQLSRSSDNLDYNKPDIDLDY

>Cotesia rubecula_Elevenin

MMNLRWALLTLTIFAWWSHSDAGRLARPDCEKFVFHPHCRGTQAKKRVIADPTSINSNMKDGNTERLCICKYLTKSGKRELLAVTKLLETMLANGVDVNMLYDTYSNMGLLSKDQNDNLRSTHHTNQLSRSSDNLDYNKPDIDLDY

>Cotesia vestalis_Elevenin

MNLRWVLLTLTIFAWWSHADAGRLARPDCEKFVFHPHCRGTQAKKRVIADPTSINSSIKDGNTERLCICKYLTKSGKRELLAVTKLLETMLANGVDVNMLYDTYSNMGLLSNKDQNDNLRSTHHTNQLSRSSDNVDYNKPDIDLDY

>Diachasma alloeum_Elevenin

MSPHQFIIMLSIFAWCTYVDGGRQTRPLNCEKYVFHPHCRGTQARKRMMSQIKNENTEQPCICAGGKDNRANAMPDVKVLEAILGNGFDVNTIYDAYAATSNRHRDYNENSRDRGNQRRSSLDNSVDVSNTAVEVDY

>Fopius arisanus_Elevenin

MLKMSLRQFIIILSIFTWCAYIDAGRQVRPLDCEKYVFHPHCRGTQARKRMISQIKNENTEQPCTCGGGKDDHANAIPNAKLLEAILANGFDVNTIYNAYAATSDRHRDYHDNSRDRGNQRRRSMIDNSVDVPNTDLEVDY

>Leptopilina heterotoma_Elevenin

MALRECVFLFSVLITFAHARAVVVNCEMYPFHSQCRGVQTKRFVIEPGHKNQHENEFKNLNSPSFKEEYNGLKNSRLWTTLLDNGLSTDDLYDVYASSSASTRGNNKKNNRKNSIKDRLRIRRIPIRDFLSDLEFLDSDY

>Microplitis demolitor_Elevenin

MNLQWALITLTILGWWSHANARGLARPDCERFVFHPHCRGTQAKKRVITDSTAINSNIKDNNAERLCICKYLTKSGKRDLLALTKLLETMLANGVDVNMLYDAYSNMGLLSNKDGNDNLRSTHRTNQLSRNSDNVDYNKPDIDLDY

>Orussus abietinus_Elevenin

MVLHRCILVLSIFACYFTCVRGRIVDCEKYPFHMQCRGIQTRKRYMGIGNGLSGESSYSRDTQTSKIPISVLKEQRHSVSSLQQPEYRSNLWSILLDKGLDNDALYEAYTTAGRRRYNGHVRRNKEDIGNNNGNNNNEEEEDNSVSDHRLNHQRIPTLLDFSSELDRLDGDY

>Telenomus podisi_Elevenin

MSLRRCIIILSLFAWFAYVRGRHVDCERFPFYSYCRGVQTRKRFVSTEEDKLNQEEYHPKPKPHSIYNVEQMLTRLEKDINNDGLLDAFLSSLIEKRRNSSKRASTKDRSKLQRTQDFLSQLNSLDNDY

>Ceratosolen solmsi_Ecdysis triggering hormone

MLIAMILIFIGYSHAAAAAKEPPAFFLKIAKSIPRIGRSDNALVANSNGPHDDPWYEDSDQLNEIVKRKIAYTSESDTKNWQHFPLAIEGPPELWRTLAGYSSNDPLHRSLNEFDNGLWPRDKRSSNLQP

>Copidosoma floridanum_Ecdysis triggering hormone

MKKPSCRFIFEKHVVSAFLIVALLLILKNQEVTADNANNNNKQVPAFFLKIAKTIPRIGRRSQPNYVGENANIAEEVPWHDTNDQVSDVSKRKDGFSPDSDTWNWQHFPLAVEGPPELWRSLAGYSRDPLYGVSNENFNNELWLQD

>Cotesia vestalis_Ecdysis triggering hormone

MLKQKINKIFIVICVTGMVLSINWTTVEADEVPAFFLKIAKNIPRVGRSGRIDEYLLTKQSKNYPRANKVGEYSPSNEYLSYPGGNDPEIHKRMVNYPAPGNIDSISWEHFPLAIEGPPELWRTLASYANDKYGTASDDIDNELWQRNKRKNVEN

>Diachasma alloeum_Ecdysis triggering hormone

MSTQVILGTCLTIAVLSCGMITVQADDEVPAFFLKIAKNVPRIGRSDKYHEYFLKQAKNVPRMGKREDYDQIAESFY

>Fopius arisanus_Ecdysis triggering hormone

MTAHFMFGQMFLGTCMIIVVLSCGVITVEADDEVPAFFLKIAKNVPRIGRSDKYHEYFLKQAKNVPRMGKREDYDQMVESFYDREPFAQRQRRMVNHASTSGLDSSAWGHFPLAIEGPPELWRALASYANDRYGTSGVNEINNDVWSRDKRSTPASP

>Leptopilina clavipes_Ecdysis triggering hormone_partial

...AKNVPRIGRSSGADNFFFKASKNIPRIGKREESSILESGYTLSPYNKDNQYVKATKRRIGYPADTGSETWTWEHFPLAIEGPPELWRTLARYSDEKAFETSDDIDNEILHRDKRAEEENQFQ

>Microplitis demolitor_Ecdysis triggering hormone

MFVVICVTGMLLSINWTTVNADEVPAFFLKIAKNIPRVGRSGRIDDYLFNKQSNNYPRANKVAEYTPSNEYLPYTGGNDPEIHKRMVNYPAPGNFESVSWEHFPLAIEGPPELWRTLASYTNDKYGTSSDDIDNEVWQRNKRKNGDAA

>Nasonia vitripennis_Ecdysis triggering hormone

MKRLVNTFISRNYILSAIFIVAVLAIIENKIVAADEPPAFFLKIAKNIPRIGRSEPYDEYAIKNSNVKDDIPWHKGEISKRRVGFSPESNTYAWQHFPLAIEGPPELWRTLAGYSHDPLYKTTDDFNNELWSRDKRTNNPEA

>Argochrysis armilla_FMRFamide_partial

...MGASFIRFGRGGGGEPILRDSNVISSESNPQVSRQPRFKSPDIVIRFGRSGSQNAWTEQAVKRARSDLNFIRFGRDVQILPEEIDLSVICSELFSGNSGNSKHLSLYLFRLFRLCSILQGIDDDYRNNLDFVDGNSADIRHE

>Ceratosolen solmsi_FMRFamide

MSQVAVALGLVLGLQVWLSLCAGLVPPESSFSEERQVYAVPRSLAGDSRERRSSGLGSSFIRFGRDRDAALGSVSQPHTRSDTVIRFGRDGLNGRLNRLRQERMRRLARLALICAAKSQEDSPASSTE

>Chrysis viridula_FMRFamide_partial

...AKRAKSDLNFIRFGRNVQILPEEIDLSVICSELLSGDSSNSKHLSLYLSRLFRLCSILQNIDDDHRSNLDFNDDNSPDTRRD

>Copidosoma floridanum_FMRFamide

MARLLWILVVVALHECQGAPGARGGAATSDEGSPRAGLEFGPLPYALRFTGGQPQQQQGQLVLEPREGRSSGMGSSFIRYGRDIAVSQPHTRSDIIIRYGRGGAGKEELLLKRDELNNRLARLRQERMQRLARLALVCANKSQEDLEAPSSAEDRIIREMCRDAPLHPTVPDFV

>Cotesia glomerata_FMRFamide

MIAYGLLYSLVIIGNGILSSATILSPLKVDPSSLHLYKTNFRPVNELDYILKRSGVRTADETPDSKERRSQMGSSFIRFGRNHMANNGENIEYSLNTVNDLDTSSRIPRGRSDVIIRFGRSGSGKLDSMILRNNKLLKVIPIEIFCADILAGENSPELTRLCNSFMSNDDKNE

>Cotesia rubecula_FMRFamide

MIAYGLLYSLVIIGNGILSSATILSPLKVDPSSLHLYKTNFRPVNELDYILKRSGVRTADETPDSKERRSQMGSSFIRFGRNHMANNGENIEYSLNTVNDLDASSRIPRGRSDVIIRFGRSGSGKLDSMILRNNKLLKVIPIEIFCADILAGDNSPELTRLCNSFMSNDDKNE

>Cotesia vestalis_FMRFamide

MIAYGLLYSLVIIGNGILSSATILSPLKVDPSSLHLYKNNFRPVNELDYILKRSGVRTADETPDSKERRSQMGSSFIRFGRNHMANNGENIEYSLNTVNDLDASSRIPRGRSDVIIRFGRSGSGKLDSMILRNNKLLKVIPIEIFCADILAGDNSPELTRLCNSFM

>Diachasma alloeum_FMRFamide

MINHGLIYGLLITMNLVLVKGTVMRPLKIDSSPLSIYKTSLDNELDYVMKRSGLQNVDDSPDSKERRSQMGSSFIRFGRNHHQGSLESPDDSANNLNNVNSGSSRTPRGRSDVIIRFGRGGLSSLDFPRHSSVGTSVTIPTLNLQKLAVICPTILTTPESALHNDLLVRLCTSLSDNNY

>Diachasmimorpha longicaudata_FMRFamide

MINHGLIYGLIITMNLALAKGTVMRPLKIDSSPLSIYKTSLDNEFDYVLKRSGLQNVDDSPDSKERRSQMGSSFIRFGRNHHQGSLESPDDSANNLNEVNP

>Fopius arisanus_FMRFamide

MINHGLIYGLLITTNLMVVRGTILRPLKMDSPSHVYKMSLDNEFDYVLKRSGSQNVEESPDSKERRSQMGSSFIRFGRSHQANLESPDNSGNNMNSDDSGSSRIPRGRSDVIIRFGRSGVQSFNFPRVSSASRSSMIPLNLTKLAAICPSILTNPESALHNDLLLRICTSLTLQSSDFDVNQWNN

>Megastigmus spermotrophus_FMRFamide

MSHLSLAIGVVLVLHCCLSITASPSSDLLSEPGYSSNQQQQQQQQQQERNLEYALKRSLLIPASDGLLSLDSRERRSSGSNMGSSFIRFGRGGDLDAVSQPHTRSDIIIRYGRDGLNNGRLTRLRQERMRRLARLALICAAKNQEDAPASSAEEKILREICKDAPLHPTVPDFV

>Microplitis demolitor_FMRFamide

MIAYGLIYGLVIIGNSILSSATILSPMKGGDPSSLHLYKTNLRPLNELDYILKRSAGAVTNVDESPDSKERRSQMGSSFIRFGRNHMTGTGENIEYSLNGLSDVDSSSRIPRGRSDVIIRFGRSGSGKIDSMILRNNKLLKVIPIDIEQLGIVCSDILAADNSSAELARLCNSFMSNDEKNE

>Nasonia vitripennis_FMRFamide

MSRLALLLGVLLGLQYYGLVAGSPVSSADYPEQQQQQQQQQQERGLEYVQLKRGLLALSAAELLDSRERRSSSSGGNLGSSFIRYGRNGDDLGLSQPHTRSDVIIRYGRDGLNGRLNRLRQERMRRLARLALICAAKSQEEGPANSAEEKILREICKDAPLHPTVPDFV

>Pteromalus puparum_FMRFamide

MAQLALVLGIVLGLQYYGLTAASPVSPDYLEQQERGGLEYALKRGLFLPLSAADVLDSRERRSSSGGNMGSSFIRYGRNSDDLGVSQPHTRSDVIIRYGRDGLNGRLNRLRQERMRRLARLALICAAKNQEESPANSAEEKILREICKDAPLHPTVPDFV

>Telenomus podisi_FMRFamide

MITHVIFCGLVVVCQILLASTSPLKLEGYSGLGDLETVLKRAKQELAHEQDAKERRSNAGSSFVRFGRSRKSLFSEIDQAQDYPMDHPQTRSDVIIRYGRSDKKNSHKPRRSDSGFIRYGRSSMEKDLQLLCSAFEETSLDQILHFYPAELVRLCESLEEERAKK

>Trichogramma pretiosum_FMRFamide

MGKVHLREGAVVLLALLQMGLCGPSSDLMSDYGNESQEGQLQGASDELAMKRNSLLSIPPEALLLDSRERRSNTPAGSSFIRFGRSGSSNGGAISPPHTRSDVIIRYGRDGAAGPNSRLTRLRQERMRKMARLALLCAESRSQQDDYAMSSAEDKILREICRDTPLHPTVPDFV

>Argochrysis armilla_Insulin-like peptide 1

MMSVYRYKVLIVIILMALLMPRSGNSQLNMFQYTRKRSAMSTVHKYCGKNLSNALKVICDGVYNQMFKKNGQEMEMDDYPFPYDESYPFRSRASANAMMGRFGGARLRRQSRGVYDECCIKACSLEEMSSYCGRR

>Ceratosolen solmsi_Insulin-like peptide 1_partial

MSTYRLSSLATLALLGLIVGQLAIAQSDIYQYEEKRQGASKYCGKHLSNMLQLVCHGLYNSMFKKSGQEMEMEDYPFAYEDSYPFRSRASANAMLGRFGGSRFRRNTRGVHDECNIGESSGTARHLNGTV...

>Chrysis viridula_Insulin-like peptide 1

MSVYRYKVLIVIVLMALLMPRSGNSQLNMFQYTRKRSAMSTVHKYCGKNLSNALKVICDGVYNQMFKKNGQEMEMDDYPFPYDESYPFRSRSSANAMMGRFGGPRLRRQSRGVYDECCIKACSLDEMSSYCGRR

>Cotesia glomerata_Insulin-like peptide 1

MSTYRCNVIAILVVVVLLAINLTKGQSENFPYGEKRQEGSATHYCGKRLSNALQIFCNGVYNSMFKKSGFEMEMDDYPYAYDYPLRSRASANAMMGRFGGARFRRQSRGVHDECCVKPCSISELMSYCGN

>Cotesia rubecula_Insulin-like peptide 1

MSTYRCNVIAILVVVVLLAINLTKGQSENFPYGEKRQEGSATHYCGKRLSNALQIFCNGVYNSMFKKSGFEMEMDDYPYAYDYPLRSRASANAMMGRFGGARFRRQSRGVHDECCVKPCSISELMSYCGN

>Cotesia vestalis_Insulin-like peptide 1_partial

...MFKKSGFEMEMDDYPYAYDYPLRSRASANAMMGRFGGARFRRQSRGVHDECCVKPCSISELMSYCGV...

>Diachasma alloeum_Insulin-like peptide 1

MSTCRSNVIVTLTLAVAFTLQLINAQPDIYQYGEKRQDDSSARKYCGKHLSNALQLICDGIYNPMFKKSDQEMEIDDYPYTYDYPLRPRANANTMMGRFSGVRFRRESRGVHDECCVKPCSMPELMSYCGH

>Fopius arisanus_Insulin-like peptide 1

MSTYRSNVIVTLVLAVAFILHLINAQSDIYQYGEKRQDGSTARKYCGKHLSNALQLICDGIYNPMFKKSGQEMEMDDYPYAYDYPFRTRASANAMMGRFGGVRFRRQSRGVHDECCVKPCSMSELMSYCGH

>Ganaspis sp._Insulin-like peptide 1

MSTHRLYVLVTLVLAASLLIPQLVKAQLDLFKHVQKRSGMNKHYCGKHLSNALQLMCNGQYNSMFKKSGQEMEIDETPYVFDDFFPFRTRQSANAMIGRFGGGRFRRYSRGVHDECCVKPCSIEELTSYCGL

>Leptopilina boulardi_Insulin-like peptide 1

MSTHRLYVLLTLVLAASLIIPQLVKAQLDLLQNVQKRSVVNQHYCGNRLSNALQLFCNGQYYSMFKKSGQEMEVDETPFAYDEMYPFRSRQSANAMIGRFGGGRFRRYSRGVHDECCAKPCSIDELTSYCGF

>Leptopilina clavipes_Insulin-like peptide 1

MSTHRLYVLVTLVLAATLIIPQLVKAQLDLFQHVQKRSGMNKHYCGKHLSNALQLMCNGQYNSMFKKNGQEMELDETPFAYDELYPFRSRQSANGMIGRFGGGRFRRYSRGVHDECCAKACSLEELTSYCGL

>Leptopilina heterotoma_Insulin-like peptide 1

MSTHRLYVLVTLVLAASLIIPQLVKAQLDLFQHVQKRSGMNKHYCGKHLSNALQLMCNGQYNSMFKKSGQEMEIDETPFAYDELYPFRSRQSANAMIGRFGGGRFRRYSRGVHDECCAKPCSLEELTSYCGL

>Megastigmus spermotrophus_Insulin-like peptide 1

MSTYRLNSLVTLALVGLVVFLQLANAQSDIYQYEKRQTSAKYCGQKLSNTLQLICHGSYNSMFKKNGQEMEMDDYPLDDSYPFRSRAKATAIIRGRFGGFGGGRFRRDTRGVYDECCLKSCSIQEMRSYCADPL

>Microplitis demolitor_Insulin-like peptide 1

MSTYRCNVTAILLVVGLLVINFTKGQSDNFPYGEKRQEGSATHYCGKRLSNALQLFCNGVYNSMFKKSGLEMEMDDYPYAYDYPLRSRASANAMMGRFGGARFRRESRGVHDECCVKPCSIPELMSYCGN

>Nasonia giraulti_Insulin-like peptide 1

MSTSRLSGLVTLTFAILLLLQLVQAQSDVYQYEQKRQGASKYCGQQLSNALQLVCHGRYNPMFKKSVGQGMEMDDYPFNYDDSYPFRSRAVANAMMGRFGAGRFRRDSRGVHDECCLKSCTMNEMRSYCAIPQ

>Nasonia vitripennis_Insulin-like peptide 1

MSNMSTSRLSGLVTLTFAILLLLQLVQAQSDVYQYEQKRQGASKYCGQQLSNALQLVCHGRYNPMFKKSVGQGMEMDDYPFNYDDSYPFRSRAVANAMMGRFGAGRFRRDSRGVHDECCLKSCTMNEMRSYCAIPQ

>Psyttalia lounsburyi_Insulin-like peptide 1

MSTYRFNMFLAMAVPMAFILQLCSAQLDIYHYDEKRQDGSSPHKYCGKHLSNALQLICDGIYNPMFKKSDREMEIDDYVYAYDYPLQVRPNANAVMGRFGDVRFRRQSRGVHDECCVKPCSMAELMSYCGH

>Pteromalus puparum_Insulin-like peptide 1

MSTSRLSGLVTLTFAILLLLQLVQAQSDVYQYEQKRQGASKYCGKQLSNTLQLVCHGLYNSMFKKSVGQGMEMDDYPFNYDDSYPFRSRAVANAMMGRFGAGRFRRDSRGVHDECCVKPCSLSEMLSYCAKPQ

>Telenomus podisi_Insulin-like peptide 1

MSRTQASGLLFSLLAVVIVCQMSAQQSIYQFVRKRPSNAMNKYCGKNLANALHLLCNGLYNSMFKKRNQEMIDPVYDNSFPFTSRVVAKTMMGRFRGNKFRRQVRGVHDECCAKPCSIDELSSYCAQ

>Argochrysis armilla_Insulin-like peptide 2

MTRMEGNTMRGSRCCWILRFVLMTLVVLSVLQLGQAAPYRKSHQRTLRLCSRSLSDALYLVCRERGGYNEPFSYSEEDEPRGYTGPGLVEECCYHSCSYEQLEQYCKPSSEEKRSGVIGEEYRIVNLPYSSARTEESGEEGPRLNTEQTGEAQN

>Ceratosolen solmsi_Insulin-like peptide 2

MSRWLCLKMLLLGLLLLGPLLLALTEAGPVLTGAGVGAGHRRYYRRAVRLCSRSLSDALYLLCKDRGYNEPFSSSGETEVRHTTGPGLVEECCYNACSIEQMEQYCKPRGKQAQP

>Chrysis viridula_Insulin-like peptide 2_partial

...CRERGGYNEPFSYSGEDEPRGSTGPGLVEECCYHSCSYEQLEQYCKPSSEEKRSGVIGEEYRIVNLPYSSARTEESEEEGPRLATKGVGETQN

>Fopius arisanus_Insulin-like peptide 2

MAQTYLNSTLLVILVLSMHWCAEGAPQMIRLCSKSLSDALYLTCMGRGYQDFITYSDGLDPKVTLGTGLVEECCHQPCTYKQLEQYCKPLPSEENSNSRIEESLRIVNLPYSSRRTVTKEGAEDEVQIKNSIKEVHEESP

>Diachasma alloeum_Insulin-like peptide 2

MSQFNYLNLMFIISLVLGAYWCVESAPFPKTIRLCSKGLSDALYLVCWGRGYNEPVPYTGGRHSKRSRSYDTGLVEECCHQSCSLQHLQLYCKPLASGENSNEGM

>Cotesia glomerata_Insulin-like peptide 2

MYIIKFTFLSRRLSVILLGLLLFSLTLVESRPHQKTIRLCSKSLSDALYLVCNNRGFNEPFSNNGDDNSKASGGPGIVEECCHRWCSYEQLEQYCKPPSN

>Cotesia rubecula_Insulin-like peptide 2

MYTIKFTFLSRRLSVILLGLLLFSLTLVESRPHQKTIRLCSKSLSDALYLVCNNRGFNEPFSNNGDDNSKAPTGPGIVEECCHRWCSYEQLEQYCKPPSN

>Cotesia vestalis_Insulin-like peptide 2

MYTIKYTFHSRRLSVILLGLLLFSLTLVESRPHQKTIRLCSKSLSDALYLVCNNRGFNEPFSNNGDDNTSKAPTGPGIVEECCHRWCSYEQLEQYCKPPSN

>Microplitis demolitor_Insulin-like peptide 2_partial

...LGLLLFLMTPVESRPHQKTIRLCSKSLSDALYLVCSTRGFNEPFSNNGDDNSKAPTGPGIVEECCHRWCSYEQLEQYCKPPSN

>Ganaspis sp._Insulin-like peptide 2

MKPSIASRFCGACTCIIVTFVVIQLIGFSDSKPYGLSGGQQTIRLCSRSLSDALYLICQGRGYNSPFSYSNEDETRTTSTNGPGLVDECCLQSCNIKQLELYCKPPNDDSTSQDGMDESVKIVNLPDSLETKQLDDVKLETLKHS

>Leptopilina clavipes_Insulin-like peptide 2_partial

...ECCLQSCNIKQLELYCKPPNDDSTSQDGMDESVRIVSLPDSLEAKPSDDITRGTLKHSR

>Leptopilina heterotoma_Insulin-like peptide 2

MKSSIATRNCGAWTCVFVTLVVVQIFGFSYSMPFGTGQQTIRLCSRSLSDALYLICQGRGYNSPFSYSSEDDTRTTNTNGPGLVDECCLQSCNIKQLELYCKPPNDDSTSQDGM

>Megastigmus spermotrophus_Insulin-like peptide 2

MMLALLVIALVIDNHQADAAAITAIHARFPRTTVRLCSRSLSDALYLLCKDRGFNEPFSTSSEVDSKHVSAGPGLVEECCYKACSVEEMAQYCKPEKTSN

>Nasonia giraulti_Insulin-like peptide 2

MYPSDSDRTTSTTRRSIGSRCWWLCLLLVLLVLSLELTRHQAEANNIHRRFYRRAVRLCSRSLSDALYLLCKDRGYNEPFTSSETEVRHTTGPGLVEECCYNSCSIEQMEQYCKPRNKA

>Nasonia vitripennis_Insulin-like peptide 2

MYPSDSDRTTSTTRRSIGSRCWWLCLLLVLLVLSLELTRHQAEANNIHRRFYRRAVRLCSRSLSDALYLLCKDRGYNEPFTSSETEVRHTTGPGLVEECCYNSCSIEQMEQYCKPRNKA

>Pteromalus puparum_Insulin-like peptide 2

MYPSDSDRTMTTTTTRRTAGSRCWWLCLLLVLLVLSLELTRHQAEANNIHRRFYRRAVRLCSRSLSDALYLLCKDRGYNEPFTSSETEVRHTTGPGLVEECCYNSCSIEQMEQYCKPRNKA

>Trichogramma pretiosum_Insulin-like peptide 2

MATIRMTLVLFALMLLIGSALLPAPSGGGGVEAFSLHRRLPRRSVRLCSRSLSDALYLLCKDRGYNEPFSSSDEEEIRHATGPGLVEECCYNACSIQQMEQYCKPRKDKKDGEQQQAGGAQSKR

>Argochrysis armilla_ITG-like

MRNMKVQRNIAVTVVALLSTMHFGAEAWGGLFNRFSPEMLSNLGYGGHGGYLSRTGLLQRPLSDSYNGPYGNSLEEVLDDPCYKKKCVTNEHCCAGSVCVVIDGDIGTCVYEYGLKQGELCRRDNDCETGLMCADSASGDIRTCQPPITGNKQYSEECNMSSECDISHGLCCQMQRRHRQTARKVCSYFKDPLVCIGPVAVDQIKSVIQYTSGEKRITGQGNRLFKRMPFA

>Ceratosolen solmsi_ITG-like

MKTYVMLTLAVLSSSINGEVDAWGGLFNRFSPEMLSNMGYGSHGGYPGRSSAFLQHGGSFNDNGLDEGAEEPCYERRCNTNEDCCAASVCINPDGEWPEGRCMFIYGLKQGELCRRDNDCETGLMCAEVAGSDSLSCQPPVTSNKQYSEQCSMSGECDISRGLCCQLQRRHRQIPRKMCSYFKDPLVCIGPVAANQVKQIVQYTSGEKRITGKQNRLYK

>Chrysis viridula_ITG-like

MMAHRNIALTVVALLSTMHFEAEAWGGLFNRFSPEMLSNLGYGGHGGYLSRTGLLQRPLSDNYNGPYGNSLEEVLDDPCYKKKCVTNEHCCPGSVCVIIDGDIGTCVYEYGLKQGELCRRDNDCETGLMCADSASGDIRTCQPPITGNKQYSEECNMSSECDISHGLCCQMQRRHRQTARKVCSYFKDPLVCIGPVAVDQIKSVIQYTSGEKRITGQGNRLFKRMPFA

>Copidosoma floridanum_ITG-like

MSPKLCVIAVVVIALSSDSLNGAEAWGGLFNRFSPDMLSNMGYGGGHGGIMSRPSAYLQQAGSLSENNLDEGADEPCYERRCTTNEDCCSMSVCINFEDGEWPEGRCMFVYGLKQGELCRRDSDCETGLVCAELAGTDSLSCQPPVTSNKQYSEQCSMSSECDIGRGLCCQLQRRHRQIPRKMCSYFKDPLVCIGPVATDQVKKIVQYTTGEKRITGKDSRLYKQGFP

>Cotesia glomerata_ITG-like

MINKLGILIGIVMMQQHIEVNAWGGLFNRFTPEMLSNLGYGGHSGGYKPSYMQRPLTGNYANNFAEGLETIDDPCEDRRCVSNEYCCSGQICIFDRDNEGFCVYPFGLKQGELCRRDSDCETGLMCTDVIGADTRSCQPPITSNKQYSEECTISSECDITRGLCCQVQRRHRQSTRKVCSYFKDPLVCIGPVATDQIKSVVQYTSGEKRITGQNNRILFKRGLFI

>Cotesia rubecula_ITG-like

MINKLGILIGIVMMQQHIEVNAWGGLFNRFTPEMLSNLGYGGHSGGYKPSYMQRPLTGNYANNFAEGLETIDDPCEDRRCVSNEYCCSGQICIFDRDNEGFCVYPFGLKQGELCRRDSDCETGLMCTDVIGADTRSCQPPITSNKQYSEECTISSECDITRGLCCQVQRRHRQSTRKVCSYFKDPLVCIGPVATDQIKSVVQYTSGEKRITGQNNRILFKRGLFI

>Cotesia vestalis_ITG-like

MIHKLGILIGIVMMQQHIEVNAWGGLFNRFTPEMLSNLGYGGHSGGYKPSYMQRPLTGNYANNFAEGLETIDDPCEDRRCVSNEYCCSGQICIFDRDNEGFCVYPFGLKQGELCRRDSDCETGLMCTDVIGADTRSCQPPITSNKQYSEECTISSECDITRGLCCQVQRRHRQSTRKVCSYFKDPLVCIGPVATDQIKSVVQYTSGEKRITGQNNRILFKRGLFI

>Diachasma alloeum_ITG-like

MIMGKSNIFCTLLIVGILEMNGGVEAWGRLFNRFTPEMLSNLGYGGQGGYRPSSLQRPLVGNYGNDFGEALQFAGDDDSCDHRPCGRNEYCCPGQICLANDADGGPEGTCFYFRGLKQGELCVRDNDCESGLMCGDVPGGDTRSCQPPVTSNKQYSEECLMSRECDISRGLCCQIQRRHRQTTRKICSYFKDPLVCIGPVATDQIKSIVQYTSGEKRITQGQHNRNLLYKRGLFI

>Diachasmimorpha longicaudata_ITG-like

MIMMKSSIFCTLVVLGILEMNGEVEAWGGLFNRFTPEMLSNLGYGGQGGGYRPSSLQRPLVGNYGNDFGDGLQFVGDDDSCNHRPCGRNEYCCPGQICLANDADGGPEGTCYYFRGLKQGELCVRDNDCETGLMCGDVPGGDTRSCQPPVTSNKQYSEECLMSRECDITRGLCCQVQRRHRQATRKVCSYFKDPLVCIGPVATDQIKSIVQYTSGEKRITQG...

>Fopius arisanus_ITG-like

MGKSCFICAIVVAVILAMNGGADAWGGLFNRFTPEMLSNLGYGGQGGYKPSYLQRPLVGHYGNDFGEGLQFAGDDDPCDHRPCGSNEHCCPGQICMNVAGGPEGTCFYAWGLKQGELCARDNDCETGLMCADVAGGDTRSCQPPVTSNKQYSEECLMSRECDITRGLCCQIQRRHRQATRKVCSYFKDPLVCIGPVATDQIKSIVQYTSGEKRITQGQHNRNLLYKRGLFL

>Ganaspis sp._ITG-like

MKTHTGIILAIVIITICSINREIEAWGGLFNRFSPEMLSNLGYGSHGGYQGKSSYLQRPGLSDGFTGNSLEESADEPCYGRRCTANEHCCPGSVCVDVDGIIGSCLFAYGLKQGELCRRDNDCETGLMCSEVAGGETRSCQPPVNSNKQYSEECSMSGECDISRGLCCQLQRRHRQTPRKVCSYFKDPLVCIGPVATDQIKSVIQYTAGEKRITGQGNRVYKRTPFV

>Leptopilina boulardi_ITG-like

MKTHTGIIFAIVFVTTCSIYTEVEGWGGLFNRFSPEMLSNLGYGSHGGYQGKSSYLQRSGLSEGFTGNSLEESADEPCYGRRCSANEHCCPGSICVDMDGIIGSCLFTYGLKQGELCRRDNDCETGLMCSEVAGGETRSCQPPVNSNKQYSEECSISGECDISRGLCCQLQRRHRQSPRKVCSYFKDPLVCIGLVATDQIKSVIQYTAGEKRITGQGNRVYKRTPFV

>Leptopilina clavipes_ITG-like

MKTHTGIIFAIVIIATCSIYTGVEGWGGLFNRFSPEMLSNLGYGSHGGYQGKSSYLQRSGLSEGFTGNSLEESADEPCYGRRCTANEHCCPGSVCVDVDGIIGSCLFAYGLKQGELCRRDNDCETGLMCSEVAGGETRSCQPPVNSNKQYSEECVTSGECDISRGLCCQLQRRHRQSPRKVCSYFKDPLVCIGPVATDQIKSVIQYTAGEKRITGQGNRVYKRTPFV

>Leptopilina heterotoma_ITG-like

MKSHTGIILAIVIITTCSMHREVEGWGGLFNRFSPEMLSNLGYGSHGGYQGKSSYLQRSGISEGLTGNSLEESADEPCYARRCTANEHCCPGSVCVDVDGIIGSCLFAYGLKQGELCRRDNDCETGLMCSEVAGGETRSCQPPVNSNKQYSEECSMSGECDISRGLCCQLQRRHRQSPRKVCSYFKDPLVCIGPVATDQIKSVIQYTAGEKRITGQGNRVYKRTPFV

>Megastigmus spermotrophus_ITG-like

MKSCTVLALVALTSCSINAGVEAWGGLFNRFSPEMLSNMGYGSHGGYMGKSSAFLQHAGSYSDNGLDDGADEPCYERPCNSNEDCCPGSVCYNPEGEPEGRCIFIYGLKQGELCRRDNDCETGLMCAEVAGTDSLSCQPPVTANKQYSEKCSMSSECDISRGLCCQFQRRHRQIPRKMCSYFKDPLVCIGPVAADQVKQIVQYTAGEKRITGKNNRLYKRGFA

>Microplitis demolitor_ITG-like

MKRASGKPITMFNKIGILICMMMMQQRIEVNAWGGLFNRFTPEMLSNLGYGGHSGGYRPSYMQRPLTGNYANNFAEGLETIDDPCEERRCVSNDYCCPAQICIIDRENEGVCAYVFGLKQGELCRRDSDCETGLMCTDVIGADTRSCQPPITSNKQYSEECTISSECDITRGLCCQIQRRHRQSTRKVCSYFKDPLVCIGPVATDQIKSVIQYTSGEKRITGQNNRILYKRGLFI

>Nasonia giraulti_ITG-like

MKTTCAMLLAVLALMNGGADAWGGLFNRFSPEMLSNMGYGSHGGYLGRSSAFLQHGSSFSDNGLDDGADEPCYERRCNTNEDCCPGSVCMNPEGEWPEGRCMFIYGLKQGELCRRDNDCETGLMCAEVAGSDSLSCQPPVTSNKQYSETCSMSSECDISRGLCCQLQRRHRQTPRKMCSYFKDPLVCIGPVAADQVKHVVQYTSGEKRITGKNNRLYKRGFA

>Nasonia vitripennis_ITG-like

MKTTCAMLLAVLALMNCGADAWGGLFNRFSPEMLSNMGYGSHGGYLGRSSAFLQHGSSFSDNGLDDGADEPCYERRCNTNEDCCPGSVCMNPEGEWPEGRCMFIYGLKQGELCRRDNDCETGLMCAEVAGSDSLSCQPPVTSNKQYSETCSMSSECDISRGLCCQLQRRHRQTPRKMCSYFKDPLVCIGPVAADQVKHVVQYTSGEKRITGKNNRLYKRGFA

>Orussus abietinus_ITG-like_partial

...TFLPVVGSCFYAYGLKQGELCRRDNDCETGLVCADVVGGETRSCQPPVTSNKQYSEECNMSGECEIGRGLCCQLQRRHRQTPRKVCSYFKDPLVCIGPVAIDQVKSIVQYTSGEKRITGQGNRIFKRTPFA

>Pteromalus puparum_ITG-like

MKTTCAMLLAVFALMNGGVDAWGGLFNRFSPEMLSNMGYGSHGGYLGRSSAFLQHGSSFSDNGLDDGADEPCYERRCNTNEDCCPGSVCMNPEGEWPEGRCMFIYGLKQGELCRRDNDCETGLMCGEVAGSDSLSCQPPVTSNKQYSETCSMSSECDISRGLCCQLQRRHRQTPRKMCSYFKDPLVCIGPVAADQVKHVVQYTSGEKRITGKNNRLYKRGFA

>Telenomus podisi_ITG-like

MRANINAALMLLVLSSFVDEGVDAWGGLFNRFSPEMLSNMGYGAHGGYPGKSSTYLQRPMSGSYANSFGDSLDEAVLEKEACYGKRCTANEHCCPGSVCVDDNSVTGYCRFSYGNKHGEICRSDNDCEDGLVCTDNIEEDTRTCQYPIYSNKQYSEECNMSGECDVTRGLCCQLQRRHRQASRKVCSYFKDPLVCIGPVATNQIKSVIQYTSGEKRITSQGNRLYKRTPY

>Trichogramma pretiosum_ITG-like

MKLQVALLGLVACWMSGGSGVGKVAAWGGLFNRFSPEMLSNMGYGGGAGHGNGGFLGRQSAFVQHGGSLSDNMLDEGMEEPCYERHCQTNEDCCLEYVCIHPEGEWPDGRCMFVYGLKQGELCRRDNDCETGLVCAEVAGSDSFSCQPPVTSNKQYSESCTMSSECDISRGLCCQLQRRHRQTPRKMCSYFKDPLVCIGPVAADQVKQIVQYTSGEKRITGKNNRLYKRGYD

>Cotesia glomerata_Ion transport peptide

MQVHHQRQPSSLSTTNLSVKLATSIPSARNQHTCQPVNLLSTSITTMSGRIVNFSSPLYINRNFVSSSVLLSIIAWSVTILLISSCLDVADAGLVMGHSLGKRSYFDLGCKGVYDKSIFARLDRVCEDCFNLFREPQLHTLCRQDCFRTNYFKSCTQVLTLEDEEEKFQEMIEYLGRKK

>Cotesia rubecula_Ion transport peptide

MQLHHQRQPPLLSTTNLSVKLATSIPSARNQHTCQPVNLLSTSITTMSGRIVNFSSPLYINRNFVSSSVLLSVIAWSVTILLISSCLDVADAGLVMGHSLGKRSYFDLGCKGVYDKSIFARLDRVCEDCFNLFREPQLHTLCRQDCFRTNYFKSCTQVLTLEDEEEKFQEMIEYLGRKK

>Diachasma alloeum_Ion transport peptide

MMQRQSRQSSITVSNLCRQSLNNGTTSLRSRTTGTIVSSATSRSTLSNSTSSSSSSTCPPVLLSVLAWSVTLLLISSCLGIADAGVLMGHPLGKRSFMQLQCKGIYDKSIFARLDRICEDCYNLFREPQLHTLCRQDCFQTKYFTSCIQALLLEDEKEKFLEMVEYLGRKK

>Fopius arisanus_Ion transport peptide

MCWDTFWLFVLNVRMIVIVGHKMMQRESRQSSIAVSTLCRQSLNDGTTSLRSRGSGASGPVVPSATPKWLNHSSSSSSSTCPPVLLSVLAWSVTLLLISSCLGIADAGVLMGHPLGKRSFMELQCKGIYDKSIFARLDRICEDCYNLFREPQLHTLCRQDCFQTKYFTSCIQALLLEDEKEKFLEMVEYLGRKK

>Nasonia vitripennis_Ion transport peptide

MMQHRQERHGSRSRRAQSTSNSPVLGCRSQSRRATSTRRPLPRPAQLCSNVQHSSSPASSSIAWSPAPSYSSSSTSSSSNSMSLLSVLAWSMTLLLVSSCLGIADAGTLMGHPFSKRSFMDIQCKGVYDKALFARLDRICEDCYNLFREPQLHTLCRHNCFGTEYFTSCIQALLLEDEKEKFQDIAEYLGRKK

>Argochrysis armilla_Ion transport peptide-like

MMNRQETSNPSLANDRFAETCPAAISYDSGCPTTSSRSSSRSSSRSSARSSTPLPSLLSAVALSMTLLLVFSCLEMASAKSGVLLPALSKRSFLEISCKGVYDKSIYARLDRICEDCYNLFREPQLHSLCRKDCFTSDYFKGCIDVLLLHDEVEQIQTWIKQLHGADPLV

>Ceratosolen solmsi_Ion transport peptide-like

MMHRQETRNSYAIATERSQLLRRSMSRSESPKASLTRSPRSPRSTGRCRSGTGNGSLDHCSSASSWTTGSSSSLLSVLACSMTLLLFTSCLGIADAGALMGHPFSKRSFMDIQCKGVYDKSLFARLDRICEDCYNLFREPQLHTLCRKNCFTSDYFKGCLDVLLLQDEMEWIQTSIKQLHGADPGV

>Chrysis viridula_Ion transport peptide-like

MTLLLVFSCLEMASAKSGVLLPALSKRSFLEISCKGVYDKSIYARLDRICEDCYNLFREPQLHSLCRKDCFTSDYFKGCIDVLLLHDEVEQIQTWIKQLHGADPLV

>Copidosoma floridanum_Ion transport peptide-like

MMHRQQQSTQDSCGAAPTLKQQQLTTCWSTTDQRTTISKRPITRSSSRWSEAAATGTIVKKSGTISTRTEFRRKGITRYSNSTNNSSSSLRYSGSLFSTTCSSLFSSSSSSSNSLLSILAWSMTLLLVSSYLGIADAGSFMGHSLGKRSFLDIQCKGVYDRSLFARLDRICEDCYNLFREPQLHTLCRKNCFTTDYFKGCLDVLLLEDEMEWIKTSIKQLHGADPGV

>Cotesia rubecula_Ion transport peptide-like

MQLHHQRQPPLLSTTNLSVKLATSIPSARNQHTCQPVNLLSTSITTMSGRIVNFSSPLYINRNFVSSSVLLSVIAWSVTILLISSCLDVADAGLVMGHSLGKRSYFDLGCKGVYDKSIFARLDRVCEDCFNLFREPQLHTLCRKDCFTTYYFKGCVETLLMQDEIEQFKNAIKQLHGADPGV

>Cotesia vestalis_Ion transport peptide-like

MQLHHQRQPSSLSTTNLSVKLATSIPSARNQHTCQPVNLLSTSITTMSGRIVNFSSPLYINRNFVSSSVLLSIIAWSVTILLISSCLDVADAGLVMGHSLGKRSYFDLGCKGVYDKSIFARLDRVCEDCFNLFREPQLHTLCRKDCFTTYYFKGCVETLLMQDEIEQFKNAIKQLHGADPGV

>Diachasma alloeum_Ion transport peptide-like

MMQRQSRQSSITVSNLCRQSLNNGTTSLRSRTTGTIVSSATSRSTLSNSTSSSSSSTCPPVLLSVLAWSVTLLLISSCLGIADAGVLMGHPLGKRSFMQLQCKGIYDKSIFARLDRICEDCYNLFREPQLHTLCRKNCFTSDYFKSCLDVLLLQDEMEQIQIWIKQLHGADPGV

>Fopius arisanus_Ion transport peptide-like

MMQRESRQSSIAVSTLCRQSLNDGTTSLRSRGSGASGPVVPSATPKWLNHSSSSSSSTCPPVLLSVLAWSVTLLLISSCLGIADAGVLMGHPLGKRSFMELQCKGIYDKSIFARLDRICEDCYNLFREPQLHTLCRKDCFTSDYFKSCLEVLLLQDDMKQIQIWIKQLHGADPGV

>Ganaspis sp._Ion transport peptide-like

MMHRQQRRSTSQSLALNSCAAERLSSTFAASASQLTSPAFSTCSTSSTSSCPALLSVLAWSMTLLLVSSCLGIADAGVLMGHPFSKRSFMEIQCKGVYDKSLFARLDRICEDCYNLFREPTLHTLCKKNCFTSDYFKGCVEVLQLEDEMEQIQTSIKQLHGADPGV

>Leptopilina clavipes_Ion transport peptide-like

MMHRQQRRSTSQSLALNSCAAERLSSTFAASASQLTSPAFSTCSTSSTSSCPALLSVLAWSMTLLLVSSCLGIADAGVLMGHPFSKRSFMEIQCKGVYDKSLFARLDRICEDCYNLFREPTLHTLCKKNCFTSDYFKGCVEVLQLEDEMEQIQTSIKQLHGADPGV

>Megastigmus spermotrophus_Ion transport peptide-like

MMHRQKERDSCGPATQKHGSLSRSMSRSQVSRTSFSRSSSTRRSSSSILSSSSHSSPATSTWSSSSTSTTSTSLLSVLAWSMTLLLVSSCLGIADAGTLMGHPFSKRSFMDIQCKGVYDKSLFARLDRICEDCYNLFREPQLHTLCRKNCFTSDYFKGCLDVLLLQDEMEWIQTSIKQLHGADPGV

>Microplitis demolitor_Ion transport peptide-like

MQLRHQRQSPSLSASSLSVELANSTSNTSSRHICQPINPLPSTSMTTMSGRILNLSSPLFINRSFICSSVLLSIIAWSVTLLLISSCLDVADAGLVMGHPLGKRSYLDLGCKGVYDKSIFARLDRVCEDCYNLFREPQLHTLCRKDCFTTYYFKGCVETLLMQDEIEQFKMAIKQLHGADPGV

>Nasonia giraulti_Ion transport peptide-like

MMQHRQESHGSCSGRTQSTSNSPVLGCRSQSRRATSTRRPLPRPAQLCSNVQHSTSIACSPAPSYSSSNSMSLLSVLAWSMTLLLVSSCLGIADAGTLMGHPFSKRSFMDIQCKGVYDKALFARLDRICEDCYNLFREPQLHTLCRKNCFTSDYFKGCLDVLLLQDEMEWIQTSIKQLHGADPGV

>Nasonia vitripennis_Ion transport peptide-like

MMQHRQERHGSRSRRAQSTSNSPVLGCRSQSRRATSTRRPLPRPAQLCSNVQHSSSPASSSIAWSPAPSYSSSSTSSSSNSMSLLSVLAWSMTLLLVSSCLGIADAGTLMGHPFSKRSFMDIQCKGVYDKALFARLDRICEDCYNLFREPQLHTLCRKNCFTSDYFKGCLDVLLLQDEMEWIQTSIKQLHGADPGV

>Orussus abietinus_Ion transport peptide-like

MMYRQQRQSSASSQWSSTSPTSTTSSSSPSTWSSSSSRSSWPVPVFAWTVALLLVSSCLGLADAGVLMGHPLGKRSFMDIQCKGVYDKSLFARLDRICEDCYNLFREPQLHSLCRKNCFTSDYFKGCLDVLLLQDEMEQIQTWIKQLHGADPGV

>Pteromalus puparum_Ion transport peptide-like

MSLLSVLAWSMTLLLVSSCLGIADAGTLMGHPFSKRSFMDIQCKGVYDKALFARLDRICEDCYNLFREPQLHTLCRKNCFTSDYFKGCLDVLLLQDEMEWIQTSIKQLHGADPGV

>Telenomus podisi_Ion transport peptide-like

MMHRQLRRISSSSCQSYPASNYSDEATIVASTLPPTTISSWTTSTSTSSTLLSIFTWSMALLLVSSCLGFADAGILAGHPFSKRSFLDIQCKGVYDKSLFARLDRICEDCYNLFREPQLHTLCRKNCFTSEYFKGCLDVLLLQDETERIQTFIKHLHGAEPSI

>Trichogramma pretiosum_Ion transport peptide-like

MMMQQQKQRTPLNRLLAPVESCNESGLVDVRTSTRSSSKSPIIRNQRTLETRTRTTSSTSRAAAAAAAATTATTKTAVSAATTCPARPTSSRHVQRSTTSVAPNSSVSSLSSSSWNSSRGSSSTSTSSSIRRLSVAAWALALLLVLASSCFGGAEAGSIMGDSFSKRSFMDIQCKGFYDRSHFARLDRICEDCYNLFREPQLHSLCRNNCFTSEYFKGCMDVLLLQDEEAKIQQAIKILHGADPGV

>Cotesia glomerata_kinin_partial

...CNDSSKVEEEFGKNNKLTGVVIKPAKIFLLSKNKLKSQIWKKLWRVNQLKKNSIPDKKISKKSLLSVGFHEGPSYHMELHPSGVPISKCVHYVKMQPANKERRNKVFNAWGGKRNSIPFKLDPKIRRPSRMPFNSWGGKRQVDYYQDNAEDLNKKGINVADPGVKKPFNSWGGKRSYGHYYHLYSSDKPV

>Cotesia rubecula_kinin_partial

...CNDSSKVEEEFGKNNKLTGVVIKPTKNFLVSKNKLKSQIWKKLWSVNQLKKNSIPDKKISKKSLLSVGFHGGPSYHMELHPSVIPISKCVHYVKMQPANKERRNKVFNAWGGKRNSIPLKLDPKIRRPSRMPFNSWGGKRQVDYYQDNAEDLNKKSINVADSGVKKPFNSWGGKRSFGHYYHLYSSDKPV

>Cotesia vestalis_kinin_partial

...WRVNQLKKNPIPVDKKISKKSLLSVGFHEGPSYHMELHPSGVPISKCVHYVKMQPANKERRNKVFNAWGGKRNSIPFKLDPKIRRPSRMPFNSWGGKRRQVDYYQDNAEDLNKKGINVADPGVKKPFNSWGGKRSFGHYYHLYSSDKPV

>Diachasma alloeum_kinin

MWHLIGITILLLVSKSVDIEGSQDVYLRNNERQKMINWLEAMKMGLASHDATWKELKDSLRHLQRRSIIKVGFHDGPTMHRLSSRSPAFATWSFWPDIESDKERRNPSFSPWGGKRDSSAYKPDPKIRRPARVPFNSWGGKRDGEHTDVIKKPFNSWGGKRSLVIEDILRVPASDILRNSWHHSIINNPKSVVLDPHDFLMSDFNQMEKKEFLNAEGMAVNNNLPRVPFDPWGGRKRESDESSEIDITVAPSPHIDSE

>Fopius arisanus_Kinin

MWHVIGITVVFLVSQSVEGSQDVYLSNNEKQKMINRLDAMKMGLSLVPHDAIPKGLKESLRHLQRRSILKMDFRDGPIMHRLSSRSPEFARWAFWPDIESDKIISSMEQHRNHALVLQEGRNPSFSPWGGKRDSSAYKPDPKIRRPARVPFNSWGGKRDGEHTGVIKKPFNSWGGKRSLLIGDILRAPTGDISRNSWRHSVIDNPKLVTLDPHDFPMNDFDEMEKKEFKPEGMSINNNMPRVPFGPWGGRKRQSDESSENYMSVSPSSSYVDSE

>Orussus abietinus_Kinin

MDHMEQEGDMWAPLAVALLLSQTVFASFEDENLSNSEDAGDAGKDTKMSEFQDPIKIQNASPTPDPERDEATFKLETDPLLKLFLTRILEEAGRKGIDETENRRKRSTDEVLAEDTAKRRDGTTLSAWLYWPIRQKQVFPADHRDTAQEKKSPAFNPWGGKRFSYDLFPNIRRPMRVPFNSWGGKRDGKNIRRLAEDKRTKFFNWGGKRTSFNSWGGKRDWEIANPCRTIAEDPERNSKNALIYDKRSEDKLKDPEEVEEKTSTQNDEKRVRFNSWGGKRFIHPSLPKEISEMHNYADEFSDEWLIAPNKRDGNNARSRIIFVPWGGKRNTNEDILNPNPHLTHQSVNIRKVFFPWGG

>Argochrysis armilla_Myosuppressin

MNAAIFLVFSMTTMAALVGETLAMPPAQCSPGLLDEVPPRIRKVCAALSTIYDLSSAMENYIDDKVPVLRDNMPLPDSGVKRQDVDHVFLRFGKRR

>Copidosoma floridanum_Myosuppressin

MKYNSCAALALVCLVCVTELRIQALAALPPQCHPNKLHELPSRVTRVCMALLTIRDLGTSMEQYIMENAPMLSVDDLDALPQRDTMKRQDVDHVFLRFGKRR

>Cotesia rubecula_Myosuppressin

MNYLLSTTFIIMSVLSGEILGMPPAQCNPSILDEVPPRIRKVCDALSTIYQLGMAMENYIIDKAPILRENVPVIGNNVKRQDVDHVFLRFGRR

>Cotesia vestalis_Myosuppressin

MNYLLSTTFIIMSVLSGEILAMPPAQCNPSILDEVPPKIRKVCDALSTIYQLGMAMENYIIDKAPILRENVPVIGNNVKRQDVDHVFLRFGRR

>Cotesia glomerata_Myosuppressin

MNYLLSTTFIIMSVLSGEILGMPPAQCNPSILDEVPPKIRKVCDALSTIYQLGMAMENYIIDKAPILRENVPVIGNNVKRQDVDHVFLRFGRR

>Diachasma alloeum_Myosuppressin

MKCNTCLLMTLTITILVILSPEIYGMPPAQCSPGLLDEVPPRIRKVCVALSTIYELGSAMENYIDDKVPVIHENIPLPDSGVKRQDVDHVFLRFGRRR

>Fopius arisanus_Myosuppressin

MKCSTCLLTLTITVLVVFPGIQGMPPAQCSPGLLDEVPPRIRKVCVALSTIYELGSAMENYIDDKVPVIHENIPLPDSGVKRQDVDHVFLRFGRRR

>Leptopilina heterotoma_Myosuppressin

MKYNNAVLLLASATIVIVFSGEVFGMPPAQCSPGFLDEIPPRIRKVCAALSTLYELGTAMENYIEDKVSVLHETVPLPDSGVKRQDVDHVFLRFGKRR

>Microplitis demolitor_Myosuppressin

MNLITSTTLIILSILSGEILGMPPAQCNPSFLDEVPPKIRKVCVALSTIYELGAAMENYIDDKAPVLRENAPLINGVKRQDVDHVFLRFGKRR

>Nasonia vitripennis_Myosuppressin

MLKYSSSRSNVAMLTLLCLCAVLATLCGEAHGMPPAQCSPGLLDEVPPRIRKVCAALSTIYELGSAMENYINDKVPVLREDIPLPDSGVKRQDVDHVFLRFGRRR

>Orussus abietinus_Myosuppressin

MKCNTAMASMAFATILLVLSGHCYAMPPAQCSSGVLEEIPPRIRKVCAALSTMYELSAAMENYIEDKVPVLRENIPLPESAVKRQDVDHVFLRFGRRR

>Pteromalus puparum_Myosuppressin

MMKYSNSSSGSNGNSGNVAMLTLLCLCAFLATFCGEAHGMPPAQCSPGLLDEVPPRIRKVCAALSTIYELGSAMENYINDKVPVLREGIPLPDSGVKRQDVDHVFLRFGRRR

>Trichogramma pretiosum_Myosuppressin

MASKVSISSLLTLAVIMVLATLLAQHQVHAMPPTQCSPNLLDEVPPRIRKVCSALSTLYELGSAMENYINEKVPVLRENIPLPDSGVKRQDVDHVFLRFGRRR

>Cotesia glomerata_Natalisin

MRGFILILIHSIAIIECENQVGSQIQFPKDPNGQIQDTKFHSQTRFESSNQKLQVKSPGILSKFDSECNCPEKTYIVFSSADSMKKFQRRSSSGGRMVEHPFYVDEPGWVMLSESNTNLMSNDNKLIKADQDPFYLARGRKSSMTVNFPFSNEKKRSFNLKDRQKRSDISDLLHEPFFISRGKKKSYNDYYYHRGDTRVNENNRGIYLYDKRNEGSDNNGYSYKLLEDNKDTDGQSRGRRDFFDQLLRENDPFYVTRG

>Cotesia rubecula_Natalisin

MRGFILILIHSIAIIECKNQVGAKIQFPKDFNGQIPDTKFHSQTRFESTNQKLQVKSPGILSKFDSECNCPEKTSIVFLSVDNMKKFQRRSSSGGRMVEQPFYVDEPGWVMLSESNTNLMSNDNKLIKADQDPFYLARGRKSAMKMNFPFSDEEKRSFNLKDRQKRSDISDLLHEPFFISRGKKKSYNDYYYHRGDTRVNENNRGIYLYDKRNEGSDNNGYSYKLLEDNDDTDGQSRGRRDFFDQLLRENDPFYVTRG

>Diachasma alloeum_Natalisin

MRIINTLFIIILLSASIRDGSCTPTRIILDGNARDLAAPASTVWDPRDTYRGLFSPRTPQFDMYRPTMDQLRNFGYAMGQIGDDRLFSPRTPQFDMYRPTMDQLRNLGYAMGQSGDGRLRTGFSNPPRPSRILATRCENLDDVKKRNFLMSFGLGTGVRCSRTIREWTLQEPLYVDEPGWVSLEIKGDGNEDPGVNGDDPFIMARGKRFIDKIRVDSANVPQTSSSKPTREVRRSEGVNMNGETDESMKSFYRGLEKYLAARSGYVQPRDKHSDVMDILNEPFFISRGKKSRGKKNDFQGITNLEFHSTNSPGNYRSIRDRRGEIVEQLLKEQDPFYIARGKRSANDLSALLRDWKK

>Fopius arisanus_Natalisin

MSSLFVFVLLSASIRRGSCTPTRVILDGNTRELPAPLTSWYLWDTDGGLFSPRIPQFDMYRPGKAQGRNFGYYPLGRMRAERLPMGFLNGRQPTRLLETRCENLEDLKRQDFFMSSGTPIRCRRTIRQWTMQEPLYVEEPRWIPLDVKGDFNEEPGFNEEDPFILARGRRSTDKNQSNSSRAKREIHQSANMIEDTDDRMKNFYRELERYLAGRSGYVQPRNKHSDIMDILNEPFFISRGKKSRSKKNDLQRAVDSDVHLQSSPGSYRPVRDRRGEIVEQLLKEQDPFYIARGKKSTNDRLSIIEDWKK

>Microplitis demolitor_Natalisin_partial

...MSTNLTRALIQISSRSKRVDKTNKLSRRRRTLSGWLLERPFYVDEPGWVSLTESDSNYLLNDNKLIKTAEQDPFYLTRGKKNSNLINLKSLIGNGKKNNLPRRYKHSDILDILHEPFFISRGKKNDNNNNVNDDYYGSDSSVNENNRGIYDKRNEGTVNDGYKLLEYDDDDTNRSLRDRRGDFFDQLLKENDPFYVTRG

>Ceratosolen solmsi_Neuroparsin

MRASHTDCALVLLLLLGILPLFLGHPTIIERGEQRSECEGCADECEKCKYGFAFSNWCGLKECLKGPGEMCGGVRNKYGACGEGMYCRCNKCTGCSIDTLECFSGFCPIYEQMQLRHPEHMQGLQLDK

>Chrysis viridula_Neuroparsin_partial

...ISKVCGTLVCKRGPGGNCGGPSDSWGICGEGLMCNQCNKCVGCSLDTYFCDWTPCLPQKNPVPHRTDTFTIEK

>Copidosoma floridanum_Neuroparsin

MPVSRNTCCLTLVVLLVLLLRDALTLSIGRPYPTIIERSEQRSECESCAGECDKCKYGHAFSRFCGVEECLKGPGESCGGMRNKYGECGDGMYCRCNKCNGCSSDTLECHSVVCPIYETRSGHPVAHTNSLMLLDK

>Diachasma alloeum_Neuroparsin

MLTIQAISMVAFLAILPTGVLCHPALRIRDVERAGCRPCGDECEACEYGVAYSPFCGVLECRKGPDEHCGARGTCGDGMYCLCERCFGCSTDNLKCSDLTPTCLPRETRYRNLQRSRQFTMV

>Fopius arisanus_Neuroparsin

MLSIRVISIFALLIVLPTGLLSHPFIRLSDLEKMSCRPCGEDCDGCEYGVVFSPLCRALECRKGPDEHCSTENSCAEGLSCICEKCIGCSIEKLKCSDSILTCLPRQGRYKHLERSRQFSMV

>Ganaspis sp._Neuroparsin

MAIQKVCAILLVTLLPLCIAHPMIKHLEEEKFLCESCGDDCDKCKFGVTFSSLCEVWQCQRGPGEICGGRGDKYGNCGEGLSCHCNKCVGCSTDSLDCYVNNCLPRTEIQLSHPNHFNEFILGK

>Leptopilina boulardi_Neuroparsin

MASQSICTFLLVTILPLCIAHPMIKDREEERLLCETCGDDCDKCKFGVTFSSLCEVWQCQRGPGEICGGRGDKYGVCGDGLSCHCNKCVGCSIDSLDCYVNNCLPRTEIQLSHPNRFNELILGK

>Leptopilina clavipes_Neuroparsin

MASQSMNVFPLVTILPLCIAHPMIKDREEERLLCETCGDDCDKCKFGVTFSSLCEVWQCQRGPGEICGGRGDKYGVCGDGLSCHCNKCVGCSIDSLDCYVNNCLPRTELQLTHPNRFNELILGK

>Leptopilina heterotoma_Neuroparsin

MASQSINLFLLVTILPLCFTHPMIKDREEERLLCETCGDDCDKCKFGVTFSSLCEVWQCQRGPGEICGGRADKYGVCGDGLSCHCNKCVGCSIDSLDCYVNNCLPRTEIQLSHPNHFNELILGK

>Microplitis demolitor_Neuroparsin

MFKHQIFYYTSLKLIVIILSTFILSSASIIRVRESERRIECPRGCAGECDKCEYGVSVSQQCGVLECRKGPDERCGGHGNGACGDGMICLCERCIGCSTDTLECSSTHFDHPCLPPGINDNTDPRYLDFNNNHIFYG

>Nasonia vitripennis_Neuroparsin

MASDTLYIFCMATLILLRQVLNELAGGIKPAGGTATPPQRPQHRRTAPGLDSSDQKNDVLSADRFLGHPTIIERAVQRSECEGCGANCKQCKYGSAYSLWCGLDECLKGPGEMCGGVRNKYGECGDGMYCRCNKCTGCSIDTLECFSGFCPILEQQMQLRHQDHSMHGLQLDK

>Orussus abietinus_Neuroparsin

MQAIVTVYVLALIAGNILCSAHPARRPVCEGCGDECDKCEFGVTKSALCGISECRKGPGEVCGGPSNSWGICGDGLICSCNRCAGCSIDSLVCFTNMTCLPHQSLESRHMDFLNRLAFK

>Trichogramma pretiosum_Neuroparsin

MHEAKPITRTTRALIFLLLTSLPMLPAHPTIIERAEAQLQRSECDGCGDQCAKCKYGSAFSRWCGLRECLKGPGEICGGARNKYGVCGDGMYCRCNKCTGCSIDTLECFSGFCPVYEQQIQLRHPDNLQGLQLDK

>Argochrysis armilla_Neuropeptide F

MQSLAKAQAMSNVAHIAWCFLLITAFGPLLIHGEPEPMARPTRPKVITSPEELRRYLDSVKDFYTLNGKARYGKRAGVSSAT

>Ceratosolen solmsi_Neuropeptide F

MYFPLALFHLVSLTWIVISIGATEVRSEPQPIARPTRPKVFTSPDELKEYLDNIKIYYTLNGKARYGKRADPHPMQPTFENPSNFPRMILAISQQNQWDQQGDLALTDKEDKNKLQIMYPYERMTRYGIME

>Chrysis viridula_Neuropeptide F_partial

MQSFAKTQTMSNAAHVAWCFLLIAAFGPLLIYGDPEPMARPTRPKVITSPE...

>Copidosoma floridanum_Neuropeptide F

MRTSKEQLQVMYFVLGIIILQSITAQSDTEPMARITRPEVFSNPDDLKQFLDLVKGYYSLNGKARYGKRKDMSMDHYVCLRPSQLHEQKLLNLQNISGYKRKQEKSVLRYFQPRDCRCY

>Cotesia vestalis_partial

...LSDNIGSTLALIVLTTWIFGSGVTCDEPETMSRPTRPKMLGSSDELRKFLDVVRDYYTVSSKARYGKRGVIPAIIVHDRPHTR

>Megastigmus spermotrophus_Neuropeptide F

MRIPMALFHFVYLALVVMAIGSVIVRSEPEPMARPTRPKVFTSPEELKHYLELVRVYYTLNGKARYGKRADSYSMPHNYEPPLNLLRSILAISRQKQQQHQQDNHKHIDKEDRNMLQIIHPYDCGTRYYETME

>Nasonia giraulti_Neuropeptide F

MRVRLAMIHLTYLTLVVLALGSVSVQSEPEPMARPTRPKVFESPEELRQYLDLVKEYYSLSGKARYGKRAEPYPMQHSYEHPLNFLRMILAFSRQKQRNQMEKQELVDKDERSKMQIMQPYDRVARYYEIVE

>Nasonia vitripennis_Neuropeptide F

MRVSLAMMHLTYLTLTVLALGSVSVQSEPEPMARPTRPKVFESPEELRQYLDLVKEYYSLSGKARYGKRAEPYAMQQGYEHPLNFLRMLLAFSRQKQELVDKDERSKLQIMQPYDRVARYYEIME

>Trichogramma pretiosum_Neuropeptide F

MKSTGLAILLLTLILVEFRSVKCNPEAMMRPTRPKVFTSPEELRQYLELVKDYFTVHGKARYGKRADHQSTLINKNVYLRNLLEILMTLNMHNQLNEVEGRYESVENNNDDSHDGVNELLEKMHLFLE

>Argochrysis armilla_Neuropeptide-like precursor 1

MALSAGRLISFLLYVFVVNDLRLATVKCQDEEGSQCLPKKTFVALLRLPEVNSNLAAYSRTARIIHDSKNRNDIAHLKALSSEENDDAEICIPANVYVELFRNSAMRGHLSPIERGQKMMENPNDRGYDSFEDDSISEAEKRSLATLAKNDDLPVTIRDRKGKQEGDKRSVLTGQSVESLLEGLLDEQTRRNMPDILQEYTLPNGEIDLDALTRDYSQGKRNIASLARDYGLPSGKRNVASLARDFALPNGKRNIGAMAREHLLPMGGKRNVASLARVYMLPQNGKRNIAALARDYSLPSGKRYADEFSYDDYDDEEKRNIASLARNADWPGLAKRSVVVPPARVILRILTRHGRSLNDRYLDGIKDETLDLQKLPRESSWNGQEEAKHQTSEVRVPLGMNGGDSNHLKDKNDSNSNAEKRRKREIDFSGEYPLPVMQNANVLDYEELIEALTGQYPNTEKRFMGRIPQMGPRPTTPPTRRLGR

>Cotesia glomerata_Neuropeptide-like precursor 1

MMLIRRRCTSLLLIFLAIVVDQLQIPLVTCQEEEDTQCLPRKTFLAFLRLPEVSSNLAAYSRSARIIQDGRNDFMYLKALSTEDMSDDTKICLPAEVYFEIFSDPTMRAHLSVIERAQRMVEDLEHDYTSFEAEKRSIATLAKNDDLPASIQERYQGDDLDDDEKRSQSSMTAEEILKMYAGHTPEELGKSAESIITAYTLPSGEIDIESLSRDFHNAKRNVGALARDYALPSGRRNRASSDSDSQESSSNSNKRNIAMLARDRLLPKGNKRNIGSLGRVYIVPSYQGKRNIGALARDSMLPMGKRYLGALVKSGGYPYIYNKRNIASLAKNGLYNYVKRNIGTLARDWNLPQSRHSRSLNEKELGAARIDLDNLQRIDSAKHPEDHHHNHELDFNKLRKGSKVNKNTSILESELVDAKNKSRNKRQIDYSEEYPLPVMQNTNVLDYDDFMEALIAGYPIAEKRFMGAGSESSERHENGDDNDDGTNDDKSINQSLMGYQDVYQPSRIPQMGPRPTTPPTRRLGR

>Cotesia rubecula_Neuropeptide-like precursor 1

MMLIRRRCTSLLLIFLAIVVDQLQIPLVTCQEEEDTQCLPRKTFLAFLRLPEVSSNLAAYSRSARIIQDGRNDFMHLKSLSTEDMSDDTKICLPAEVYFEIFSDPTMRAHLSVIERAQRMVEDPEHDYTSFEAEKRSIATLAKNDDLPASIQERYQGDDVDDDEKRSQSSMTAEEILKMYAGHTPEELGKSAESIITAYTLPSGEIDIESLSRDFHNAKRNVGALARDYALPSGRRNRASSDSDSQESSSNSNKRNIAMLARDRLLPKGNKRNIGSLGRVYIVPSYQGKRNIGALARDSMLPMGKRYLGALVKSGGYPYIYNKRNIASLARNGLYNYVKRNIGTLARDWNLPQSRHSRSLNEKELGAARIDLDNLQRIDSAKHPEDHHHNHELDFNKLRKGSKVNKNKSILESQLVDAKNKTRNKRQIDYSEEYPLPVMQNTNVLDYDDFMEALIAGYPIAEKRFMGRIPQMGPRPTTPPTRRLGR

>Cotesia vestalis_Neuropeptide-like precursor 1

MMLIRRRCTSLLLIFLAIVVDQLQIPVVTCQEEEDTQCLPRKTFLAFLRLPEVSSNLAAYSRSARIIQDGRNDFMHLKALSTEDTSDDTKICLPAEVYYEIFSDPTMRAHLSVIERAQRMVEDPEHDYTSFEAEKRSIATLAKNDDLPASIQERYQGDDVDDDEKRSQSSMTAEEILKMYAGHTPEELGKSAESIITAYTLPSGEIDIESLSRDFHNAKRNVGALARDYALPSGRRNRASSDSDSQESSSNSNKRNIAMLARDRLLPKGNKRNIGSLGRVYIVPSYQGKRNIGALARDSMLPMGKRYLGALVKSGGYPYIYNKRNIASLARNGLYNYVKRNIGTLARDWNLPQSRHSRSLNEKELSAARIDLDNLQRIDSAKHPEDHHHNHELDFNKLRKGLKVNKNKSILESELVDAKNKSRNKRQIDYSEEYPLPVMQNTHVLDYDDFMEALIAGYPIAEKRFMGRIPQMGPRPTTPPTRRLGR

>Diachasma alloeum_Neuropeptide-like precursor 1

MANCKGSYCLLLVVLALVGDQLQIPVVTCQEDDVSTRCLPRKTFYAFLRLPEVSSNLAAYSRTARIIQDARNDFIHLKTLSTPGREDSDDARICLPAIVYYEIFSDPTMRAHLNAIERAQKMVEDPERVDEAGFFDSEKRSLATLAKNGDLPASIQERVQERQEDGEKRSEASLTPEELLKLYVGESSGEKPTEMGYPLPRSSLDAEPEDFFTGKRNIAALAREYALPGRRNIASMAREFGLPSGKRNVGALARDRQLPSGKRSPPYTLGRFFVIPVTTGKRNVGSLARDSALPPYGKRGIASLAKNGDFPFPKRNVGTLARDWSLPQSRHGRSLDEEEMKDAVVNRRVGEDEGSNGSSPFEAQLNDVKARGKRQADYTDEYPLPVMQNTNVLDYEDIIEAIANGFPQAAKRFMGRIPQMGPRPTTPPTRRLGR

>Fopius arisanus_Neuropeptide-like precursor 1

MEHYKGRNCCLLLVVLALVGDHLQIPVVTCQEDEVSTRCLPRKTFYAFLRLPEVSSNLAAYSRTARIIQDARNDFVHLKGLSTTSREEGDDGRICLPAYVYYEIFNDPTMRAHLNAIERVQKMMVEPERIDESGIFDSQKRSLATLAKNGDLPVSIQERAQDGQEDDEKRSEASMTPEELLKLYGGESSEEKPTEMMSYGIPSALDTESEDFFSHWGKRNLAALARESALPGRRNIASMAREFGLPTGKRNVGTLARDRQLPSGKRRPSYTLGRLFVIPVATGKRNVGSLARDSALPPYGKRGIASLAKNGDFPFPKRNVGTLARDWSLPQTRHGRSPDENEEDNRSNSFERQLDDVKARTKRQADYSDEYPLPVMQNSNVIDYEDIIEAIANGFPQAAKRFMGRIPQMGPRPTTPPTRRLGR

>Microplitis demolitor_Neuropeptide-like precursor 1

MPSSRHFTSLLLILFAIIVDQLQIPLVTCQEEEDMQCLPRKTFLAFLRLPEVSSNLAAYSRSARIIQDARNDFMHLKALSSEDTSDDTKICFPAEVYYEIFSDPTMRAHLSVIERAQRMVENPEHDAVNFETEKRSIATLAKNDDLPASIQDRYQGEDDDEKRSQSSMSPDEILKMYVGHTPEELGKSAEDIINEYTLPNGEIDVESLSRDFHSTKRNVAALARDYALPSGRRNSASADSQQSSLHGDKRNIAMLARDRLLPKGYKRNIGSLGRVYIVPSYQGKRNIAALARDSMLPMGKRYLGALVKSGGYPYIYNKRNIASLARNGVYNYVKRNIGTLARDWNLPQSRHSRSLNEKELGAARIDLDNLQRIDSTKHNDDLHAHNHDFDLNKFSKGFKLNKNKNSNKSILESELVDAKNKSRSKRQIDYSEEYPLPVMQNANVLDYDDFMEALIADYPIAEKRFMGRIPQMGPRPTTPPTRRLGR

>Argochrysis armilla_Orcokinin A_partial

...DRYFEDRSNSYQPRMLSVDGRFLRQTRRGLDSLSGATFGESKRSAAARRASSPSHQFDRYAKRNIDEIDRTAFDSFFKRNFDEIDRDGFDGLDDFTKRLSDYFTNRQRR

>Ceratosolen solmsi_Orcokinin A

MEALAKEFEKEGVAALRNQDVNAHLTKNSQNDINVVNYAHNKKVSGHRKSESPDSFKKYTEAVLYPADLISISNNKRNIDEIDRSGFSGFNKRNFDEIDRSGFSNFNKRNFDEIDRSGFSGFNKRNFDEIDRNGFSGFSKRDFDDIDRNGFSNFNKRNFDEIDRNGFSNFNKRNFDEIDRSGFSGFNKRNIDEIDRSGFSGFNKRNIDEIDRNGFSGFDKRNFDEIDRSGVPSFAKRSLPSRFKGVGHRRR

>Chrysis viridula_Orcokinin A_partial

...WVSASPIQFQEESGILRQETYPPVEYLGRYFDDRSNLYEPRIFSVDGRFLRQTRRGLDSLSGATFGESKRFAAPSYSKRNIDEIDRTAFDSFFKRNFDEIDRDGWDGFVKRLDDYLANRKQR

>Copidosoma floridanum_Orcokinin A

MEKRSVTVALLFGLVCATLLDDALTSPVQPQEENIVLNSKFFSKSPEYVEALAKELEKESIAAYMGKQMSRNPHGDNDFGLGRAEKLFQQRKIELPSGGGPYTGQLMRMESRKRNFDEIDRDSGFSGFSRKRTFDEIDRTGFPSFNKRNNLDEESDRSYFDEHVNRHQLVR

>Cotesia glomerata_Orcokinin A

MVNSRFIGSAFILAAVTCIFAVPIQLHDETMGLNEGMYASPELAEALARSYAALGTSRLQNPRDDSHAGHLRQPSRLVFERDSDLRRKSGWPNTVNGVEIYEGTLKRNIDEIDRTGFDNFVKRNFDEIDRSGWDSFVKRRIANAYFSGRQH

>Cotesia rubecula_Orcokinin A

MVNSRFIGSAFILAAVTCIFAVPIQLHDETMGLNEGMYASPELAEALARSYAALGTGRLQNPRDDSHAGHLRQPSRLIFERDSDLRRKSGWPNTINGVEIYEGALKRNMDEIDRTGFDNFVKRNFDEIDRSGWDSFVKRRIANAYFSGRQH

>Cotesia vestalis_Orcokinin A

MVNSRFIGSAFILAAVTCIFAGPIQLHDETMGLNEGMYASPELAEALARSYAALGTGRLQNPRDDSHAGHLRQPSRLVFERDSDLRRKSGWPNTVNGVEIYEGALKRNMDEIDRTGFDNFVKRNFDEIDRSGWDSFVKRRIANAYFSGRQ

>Diachasma alloeum_Orcokinin A

MGIARAISFSGLVLVFLTAASAIPLQMRGETSGLNEAVYTEPELMEALARSYGALGGGAREGRDFRIARQSRQGLDSLSGATFGESKRFDPRRKPELSNLSQMELYNGGFKRNIDEIDRAGFDSFSKRNFDEIDRAGWDSFVKRRFVDAYLASRQH

>Fopius arisanus_Orcokinin A

MGIARAISVAGLLVVFFIEASAIPLQMRGETSGLNEAVYTEPELMEALARSYGAIGGARESPMRDSRINRQSRQGLDSLSGATFGESKRFDPRRKPELSNLSQMELYNGAIKRNIDEIDRAGFDSFSKRNFDEIDRAGWDSFVKRRFVDAYLASRQH

>Megastigmus spermotrophus_Orcokinin A

MRGCYGSVWREIALAATFCAILVQASPTPPLRENIPLNAAVFSQPEYVEALAKELEKEGVGALKSQDEVGQSGRNSRDEYEGAAGYGHNKKFTGRRNSDSPNSLSRYSDSVLYPSELIDLSNRKRNFDEIDRSGFSSFNKKRNFDEIDRNGFSGFSKRNFDEIDRTGFSGFNKRNFDEIDRNGFSGFSKRNFDEIDRSGFSGFNKKRNFDEIDRSGFSGFNKKRNFDEIDRNSLSGFHKKRNFDEIDRNGFSGFSKRNFDEIDRSGVPGFAKRSVSSRSKLNGHRRR

>Microplitis demolitor_Orcokinin A

MMTTRFIGSAFIFAAITCIFAVPIQLHDETMGLNEGVYTQPELAEALARSYAALGAGRIQNSRDETRAHFRQPSRLNPGLTFENRDSDLRRKSGWPITVNGMEIYEGSLKRNIDEIDRTGFDNFVKRNFDEIDRSGWDSFVKRRIANAYLAARQH

>Nasonia giraulti_Orcokinin A

MLLVVALCCALVAGLQASPISPQREIIPLNAEAFAQPEYVQALAKELEKEGVAELKSQAEERQTRNARNDYDSPDYGHGQKFTGHRKPDSTHRLINKGFSETIIYPSDIIEYTNQKRNFDEIDRSGFSGFSKRNFDEIDRSGFSGFN

>Nasonia vitripennis_Orcokinin A

MQEWKSPKQYHYSGGNLDHIGGGNLLRKKVVKIPNEYFTETTTTSVPVETTTSDLKADRRLIGVAELKSQAEERQTRNARNDYDSPDYGHGQKFTGHRKPDSTHRLINKGFSETIIYPSDIIEYTNQKRNFDEIDRSGFSGFSKRNFDEIDRSGFSGFNKKRNFDEIDRTGFSGFNKRNFDEIDRSGFSGFNKRNFDEIDRSGFSGFNKRNFDEIDRSGFSGFNKRNFDEIDRTGFSGFNKRNFDEIDRSGFSGFNKRNFDEIDRSGFSGFNRKRNFDEIDRSGVPGFAKRSIASSRSDRIFCISIIYHAQDI

>Telenomus podisi_Orcokinin A_partial

...IQHLIIDDHQKNAPFNIDGLMKKNFDEIDASGFRGFVSKKNFDEIDRSSFNDFGKRNFDEIDASGFRGFDKKNLFGTFDLDRRDLEKYQRLHLPDYFRIGRLGPRSYLKSSFEDVGTRLQKKN

>Trichogramma pretiosum_Orcokinin A

MKLSAVFSAFLCLSITSFGNSALILPHGNALNTDVLSQAEYVEALAKELEKEGLKSQRGNRHGSKSIETSGFNHAAGFGGQRSRLADEGFSESVIYPAELARLNGNPEKRHFDEIDRNDFNTFKRNIDEIDRTAFNAFKRNLDEIDRTGFNAFKRNFDEIDRAGFGGFADKRNIDEIDRAGFGGFNRKRNFDEIDRSGVPGFAKRSAPRSSSPNNNNKLRR

>Argochrysis armilla_Orcokinin B

MMMANSHASYILPIILAVLASVFASPIQFQEESGILRQDAYPALEYLDRYFEDRSHQLKRNLDQIGGGNLVKRRENGGKLPEEREELFLRNLDHIGGGNLLRSAEEYSRDGTRAGLPHPPPKNARTVDPLGGGNFVKRSINRAGEKSFSVVETDDGILIRYD

>Copidosoma floridanum_Orcokinin B

MEKRSVTVALLFGLVCATLLDDALTSPVQPQEENIVLNSKFFSKSPEYVEALAKELEKERLEEPMRWWTLFKQMLMSKPNHEKDVFKRNLDSIGGGNLLRRDLGNVELDGNSMGSRINGASRTRRRHVYHGGGGSSLDSIGNGNLVRRDTLVLNNDDDDDDDDEQNFFDF

>Cotesia rubecula_Orcokinin B

MVNSRFIGSAFILAAVTCIFAVPIQLHDETMGLNEGMYASPELAEALARSYAALEWQQRNGNSIGARFKKRNLDQIGGGNLLRSLQEQNLPYHVRNLDPIINDHLRSLLNSYQQQNQE

>Cotesia vestalis_Orcokinin B

MVNSRFIGSAFILAAVTCIFAGPIQQLHDETMGLNEGMYASPELAEALARSYAALEWAQRNGNSIDAGIQKRNLDQIGGGNLLRSLQEQNSPHHERNSDSIVTNHILRSLLGNYQGQNQE

>Diachasma alloeum_Orcokinin B

MGIARAISFSGLVLVFLTAASAIPLQMRGETSGLNEAVYTEPELMEALARSYGALEWRNRNERSEISNLEQRNLDHIGGGNLLRREVGSPQRYRKPGVVAYQPIFARGPNGEIIPY

>Diadromus collaris_orcokinin B

MTIIRMLCSPAAVVLAIAACAAGAPVQVQRENIGLDNAAFSPPELAEALARTYEAIEWQRSRGREDEAGNDRTRNLDHIGGGNLLRNLDHIGGGNLLRNLDHIGGGNLLRDLDSSRSGEKFGTIHNKVVDLRKTWNTPVQTEHKNWSSDRGTLDPLGGGNIVRRAVNGFVEPELHLEYVGGGDLLREMNKDDKYKMSLTRARINEPELHEEYIGSYDPLREMNKDQKYTQS

>Fopius arisanus_Orcokinin B

MGIARAISVAGLLVVFFIEASAIPLQMRGETSGLNEAVYTEPELMEALARSYGAIEWASRNERSEIPTLEQRNLDHIGGGNLLRRSLAITPENPKPKIAYSILTRGPYGEMISY

>Megastigmus spermotrophus_Orcokinin B

MRGCYGSVWREIALAATFCAILVQASPTPPLRENIPLNAAVFSQPEYVEALAKELEKEDETSQLAKWWLSYQEKMQNDPDYHNRGSENHRNLDQIGGGNLLKRNLDQIGGGNLLKRNLDQIGGGNLLRRDVTQNQDTTTPYYVSSWVNLDEIGGGGNPARRNLDQIGGGNLLKRNLDQIGGGNLLKRGAYTRTEDATTPFYIIHVQRTGNLDRHTGGGHSLRRNLDQIGGGNLLKRNLDQIGGGNLLRRELDIGEQRTKRELAMRRNLDQIGGGNLLRSSPNRENSRDRYYEDY

>Nasonia giraulti_Orcokinin B

MLLVVALCCALVAGLQASPISPQREIIPLNAEAFAQPEYVQALAKELEKEGTTTESSPRIRRNLDQIGGGNLLREKEQTATLDHNQPKADDFSSLRSKRGYPVHVYEPVLPTLFGITKLTTTTTTVAPKEHREYHYSGGNLDHIGGGNLLRKKL

>Argochrysis armilla_Pigment dispersing factor

MSNSVKYLVAIVILLGLLYPTLGSMDDSERNALGSNFPYGRGLDSELQLARLLMIPQRMCHPKRNSELINSLLGLPKNMNNAGK

>Ceratosolen solmsi_Pigment dispersing factor

MKFWMNRFSRAFIIFTIICCVSASMEEISHNMMIPNNPYGRPLDTDLLARLLLLPQRLCHPKRNSELINSLLGLPKNMNNAGK

>Chrysis viridula_Pigment dispersing factor

MSNSAKYLVAIVILLGLLYPALGSMEDSERNTLGSNFPYGRGLVNIHDPTPYTDSELQLARLLMIPQRMCHAKRNSELINS...

>Copidosoma floridanum_Pigment dispersing factor

MQSWLNGFVKVSILLAIICCASASMEDESHSLVMPNNFYGRDLDNDLLVKLLLTMPQRLCHPKRNSDIINSLLGLPKNMNNAGK

>Cotesia glomerata_Pigment dispersing factor

MIHASKITANILILTVFLLIIDSTDATIEDMDREIAANFPYGRGMDNEQVQLARLLLSYPRGMGHPKRNSELINSLLGLPKNMNNAGK

>Cotesia rubecula_Pigment dispersing factor

MIHASKITANILILTVFLLIIDSTDATIEDMDREIAANFPYGRGMDSEQVQLARLLLSYPRGMCHPKRNSELINSLLGLPKNMNNAGK

>Cotesia vestalis_Pigment dispersing factor

MIHASKITANILILTAFLLIINSTDATIEDMDREIAANFPYGRGMDSDQVQLARLLLSYPRGMCHPKRNSELINSLLGLPKNMNNAGK

>Diachasma alloeum_Pigment dispersing factor

MKPSTKNVNSIFFVILLVIIVDSTLGTIDDADRDLMTANFPYGRGVDNELQLARILYLLPQRGIMCHPKRNSEIINSLLGLPKNMNSAGK

>Microplitis demolitor_Pigment dispersing factor_partial

...MVGTNFPYGRGVDSDQLQLARLLLSYPRGMCHPKRNSELINSLLGLPKNMNNAGK

>Nasonia vitripennis_Pigment dispersing factor

MRSWVSHLIRAFFVFGAILCASASMEDTSHMIMNNPYGRSLDAELITRLLLAPQRLCHPKRNSELINSLLSLPKNMNNAGK

>Orussus abietinus_Pigment dispersing factor_partial

...MLLVPHRLCHVKRNSELINSLLGLPRNMKNAGK

>Psyttalia lounsburyi_Pigment dispersing factor

MKPSTKDVTSIFFVILLVVIVDSTLGTIDETDRDLMTASFPYGRGVDNELQLARILYLLPQRGIMCHPKRNSEIINSLLGLPKNMNNAGK

>Telenomus podisi_Pigment dispersing factor

MQILFKFCLVSILLLGAVAPTLTYYQDYPLNTLMSKYFYRLKDDDLMILKLLLEPQRTNQKRNSELINTLLSLPKTINMAG

>Trichogramma pretiosum_Pigment dispersing factor

MHSEISKIIKIFIILGVIYCTSASMEDPSNTLSIPNEYYGRQLDFDLLTRLLTMPPRLCHSKRNSELINSLLGLPKNMNNAGK

>Cotesia glomerata_Prothoracicotropic hormone

MKIIILAILVIVTENVIGRTYNSGRWTNIILDPDSSNDSSELISNRDISYPGKRNGALRSLEKMHISPRDLCALSNPWQWFCPCETQYGIVDLGIGHYPQYFTSAQCIQKPCHGKFNPCKLIHYKMHVLSPRDINDINIDAEESYIQQPILPESLRSKWQLKPITVPVACIAPNEGRNN

>Cotesia vestalis_Prothoracicotropic hormone_partial

...MHISPRDLCALSNPWQWFCPCESQYGIVDLGIGHYPQYFTSAQCIQKPCHGKFNPCKVIHYKMHVLSPRDINDMNIDAEESYIQQPILPESLRSKWQLKPITVPVACVASNEGRNN

>Diachasma alloeum_Prothoracicotropic hormone

MKTIAVYMTLVTLAIRTDGRQVNFAQWPKLIDPDDSLSLQDDCLTGSCDDLFAEKRSKVLEEVEQMLPVSSSYHSSSGPWQWMCPCETQYGILDLGPGHYPRYLARAHCAPKACRNRLNQCKLINYKVHILRERDPTDGGETSDEQYVEHSVLPEPLRVKWQLKPMKIAVACIAATEGKKN

>Fopius arisanus_Prothoracicotropic hormone

MKSFTIYIALVNLAIITDGRQFNFDQWSKMIDPDDSLGFPDDCITQSCNDLYEEKRSRGFEGTNQMMAATGPYHFSIDPWEWMSCPCETAYIIEDLGSGHYPRYLSRAHCVHKTCRNRLNPCKLLDYKVHILEERDPNEPNDGQYIEQAVLPEPLRVQWQMKPVTIAVACVAVTERRKN

>Nasonia giraulti_Prothoracicotropic hormone

MKLQTFLVLLLTVQHRAKGQLGSYSDDFSDVELPADERDDCSDGTCYAEKRIILPEKIVEHRQLMPRFATKLQSVNLAESFPQPQWRPVCGCVTQHKLVNLGEGHYPRYITTARCKSKTVANRFYQCKYYDYRVHVLVKRGFNSIPKNADELDVRDVEELPLPESLHANWQLFALSVSVACVAVERA

>Nasonia vitripennis_Prothoracicotropic hormone

MKLQTFLVLLLTVQHRAKGQLESYSDDFSDVELPTDERDDCSDGTCYAEKRIILPEKIVEHRQLMPRFATKLQSVNLAESFPQPQWRPVCGCVTQHKLVNLGEGHYPRYITTARCKSKTVANRFYQCKYYDYRVHVLVKRGLNSIPKNADELDVRDVEELPLPESLHANWQLFALSVSVACVAVERV

>Pteromalus puparum_Prothoracicotropic hormone_partial

...VLLLIVHHKAKGQLGSYSDDFSDVEFPPDERDDCSDGTCYAEKRIILPEKPVEHRQLIPRFATKLLSVNSAESFPQSQWRPVCGCVTQYKLLNLGEGHYPRYITTARCKSKTATNRFYQCKYYDYRVHVLVKRGLNSILKNADEPDVRDVEELPLPESLHANWQLFAFSVSVACVAVERA

>Argochrysis armilla_Pyrokinin

MQEPSHTSATWILLLFLLYTVSSISGEYGAREGSSGAVNERTVDAGDFRTCVDGKCIKRTTQEITSGMWFGPRLGRRRRSGERSAINAEINAERDGELEGLANALGAADRLDGWLVLSIPANQKRQSTSFTPRLGRELYGEKFRQREDNVGSMDTENDLELLRSPSLYSPRLGRKLLLPSSSRLPSQLQPLLQRLQENYQRINE

>Ceratosolen solmsi_Pyrokinin

MIGTRARTIVCGLVIVIVSQVAGEYEGQSSEVLESPRLERMHSERSNDCVGNNCIVHHAEGALGAIWFGPRLGRRRRSDAKYSGKTVETLGEVLGVPSLNLVGISDAVNKRQELAYSRREGREFGNNYSTRNLVRGLLHHVEEQPPGRPEHQLTLPMIFTPRLGRSYATPTLEREIHDLLRKLQLQAQ

>Copidosoma floridanum_Pyrokinin_partial

...MAQDCEGSWCKSQHESPLRTKLWFGPRLGRKRRSFGPLFPATRASAEAYDALLADLYSVPGALKLQKLTASPQGVIGAKEKRQETTFTPRLGRDLGNVYPIYDLARLLYEQVRELEDSEQEQQTPPPNFPPRLGRNLLLTESSTTGLAREQMLRYILQQLDL

>Diachasma alloeum_Pyrokinin

MFTISCASACVRVGTLLATIAVVTCDYDGNQAASFADRNLDSNVLCNGGRCTETSDGIAGAMWFGPRLGRKRRSDEKMETIDEEIGAIADVINSGPWAWVAYSGGGDKRHTTQFTPRLGRELTDDFLQKYMAYDFDKNRLSRYIAAPEDASLQRHQSPPPPPPQFAPRLGRNLPFNPSPRLGRHLRFVQKV

>Fopius arisanus_Pyrokinin

MVATSCTSACVLVACLLAAIDLVICDYDGDQPTSLDFNGLCNGGRCSETGDGIAGAMWFGPRLGRKRRSDEKMETIDEDVEAMADVINGGPWTYVQYPGAGDKRHTTQFTPRLGRELTDDILQKYLNYDLERNRLSRYIDREFEDMTINHHQPPTPPPQFAPRLGRHLPFNPSPRLGRHLRFMRRA

>Microplitis demolitor_Pyrokinin

MSVFESNFGFGVAILFLVMIITQVSGDYDGSVENDSGLCLGPDCADRPSNGVSGAMWFGPRLGRRRRSGPVSHVNDDDINTIADAINSGPWALVSYQGTGDKRHSTQFTPRLGRELEDNLLQRYLALGKDNELYHLMDTQINPEHRQLSLPPPPLFAPRLGRQLPFMNSPRLRHLLQNSRNF

>Nasonia vitripennis_Pyrokinin

MELARLINGARTKAILCALLVIVLMANRVAGQYDGRGSDMVEGPRVERMHPETSGGCVGAHCLTQNSEGPVGAMWFGPRLGRRRRSDKFTPKKIEALSEMLGSPNWNLVTIPGGEDKRQETTFTPRLGRELENAISVYDLVRGLVSSVDDQNGKDRDQQAPPPMFPPRLGRTLLTPRLEHELRNLLRKLQMQ

>Telenomus podisi_Pyrokinin_partial

...DFSPNGSNEIETSYDNGLKILRDVGKGTNGNPIRNTKRNTVNSTSVGLWFGPRLGKRRKSTDGPEIETEELDSIAEALSSGPWAFISIPHASRTGIERRQATQFTPRLGREVSENVAMYEQDQLFNKDQDMQAQLPVDSLQQLRYMSRGEREQLSPLLQMLRKSLF

>Trichogramma pretiosum_Pyrokinin

MYRVRIRTTTIIASALFMSAIVVDTVSAQYDSPSGQEAIEGPRVERMHAEASDPCAAGQCLTQNSASQLGGMWFGPRLGRRRRSGGQAEDKLLQLPSSSVSDKKIQQLSEILAAERPSWGIITVPAETEAQSPSLRDGMSKNEYPREPPFTPRLGRELEDRLVGMQLAHSFLERLDNKLDIDDATDGQQQQQDNSQPKFPPRLGRRIPERELRRIFRRFKIYF

>Cotesia glomerata_RYamide

MIACLCIIFTLVNVAFSQPNFYTHGRYGKREETPPTGEVNFNFGSRYGRGENIEPKATNDKSNELMARVDRFYLGSRYGKRERNNNNLFGSSNGENFIDLNNIQDVLRYFYRLQRRTKQLPKDNIDYIDGENFKSKNDQYIFHKDNFIKWKPSVPKIDSAIPMSSCEQ

>Cotesia rubecula_RYamide

MIACLCIIFTLVNVAFSQPNFYTHGRYGKREETPPTGEVNFNFGSRYGRGENIELKATNDKSNELMARVDRFYLGSRYGKRKSNNNNLFGSSNGENFIDLNNIQDGLRYFYRLQRRTKQLPRDNIDYIDGENFKSKNDQYIFHKDKFIKWKPSIPKVDSAIPMSSCEQ

>Diachasma alloeum_RYamide_partial

...MVGSPAKKSDRGFLSRGRIFPASAMDLIVCLSLVLVIANVVLAENTFYTQGRYGKREESHPSAGALPYFSLSRHVRSDKKSNHASNTQDSNTVKVSSRPDRFFLGSRYGKRALPRAVELSDGSPLASLDRLETILRYLNRARRSDQRSSAPNYEIEQIYYDNGYDNGNDDNGVGDNDSKTLCQSDSSQC

>Fopius arisanus_RYamide

MSSTFAVDPIACLWIVLLLTNVILADNNFYTQGRYGKRQESRPESNKRPLFFPSRYGRSIPNSDETSGGGSKIVEISPRPDRFYLGSRYGKRGPVGDEESTGGSRFTSLSRLEAILRYLDRARRSNQHRNKQNYEIEQIYYDNGYDDGNDDNLVEDNDSKTPCQSGSQC

>Leptopilina boulardi_RYamide

MLSSSRKVRLASATLGFSSIELMIWLWISCNFVTLAFSQNNFYTSGRYGKREESAAPVGEVFLAGSRYGRSGMMGMKNSRTGPKVSDMAPRVDRFYTGSRYGKRAHMQLYNAASLKEFEEALNYLDRVQHMKREDRIKYDKNNENNEDADKQEDEQNPLVLCEMFNTSRC

>Leptopilina heterotoma_RYamide

MIANSRKVRLASAVLGFSSIELMIWLWISCNFVTLAFSQNNFYTSGRYGKREESAAPVGEVFLAGSRYGRSGMMTMKNSRTGPKVSDMAPRVDRFYTGSRYGKRAHSQFYNAAALRDFEEVLNYLDRMQHIKKEDRMKYDRNKENNEDVDKQDDEQNPLVLCEMFNTSRC

>Megastigmus spermotrophus_RYamide

MLDNLKLGKLTVWLWISCILVTAVSSQDQFHTNGRFGKRKETRTLSTFQSGSRFGRSESSMVKNTKEMPKLFEIVPRMDRFFYGSRYGKRSSSAPLQAAVHEMQR

>Microplitis demolitor_RYamide

MIACLWIIFTLVNVAFGQPNFYTHGRYGKREETLPTDQLNFNFGSRYGRGEKVELKATNENNEKSNEIMARVDRFFLGSRYGKRGINDNLIDNSNEKFIYLNNIRDALRYYYYRIRREAQQLSSDDINNTDKKNESIINNKTSLSKADSAEPMSSE

>Nasonia giraulti_RYamide_partial

MISSSRKIRRVSDYLKLDKLIVWLWISGIFLTLVSSQDNFYASGRYGKRKYALSMSQIPLCSKFDRSKDRSAGNSLKDSSLFSSARFGRSEDRNTGNSLRDSSSFSPARYGRGEDR...

>Nasonia vitripennis_RYamide

MISSSRKIRRVSDYLKLDKLIVWLWISGIFLTLVSSQDNFYASGRFGKRKYALSMSQIPLCSKFDRSEDRSAGNSLKDSSLFSSARFGRSEDRNTGNSLRDSSSFFPARYGRSEDRSTGNSLRDSSSFFPARFGRSEDRSTGNSLKDSSSFSPARYGRSEDRSSGNSLKESSFFSPGRYGRSEGHKNPKELPKFFEIKPRVDQFFIGSRYGKRSLSMLEPQPPLEALHNQRFEAAIDYLDRIKQNLAE

>Orussus abietinus_Ryamide_partial

...ARCVKNRLGPSSFGLGGIEFAAWIWIGCALFNIVLSQENFYTHGRYGKREEHQQCKCQDCFPSAEKKQLVDV...

>Pteromalus puparum_RYamide_partial

...RSSGNRLKESSFFSAARFGRSEDANNLKKSPKLFEIEPRVDRFFLGSRYGKRSSSVLEPQRAPPEAMNYQRFEAAIDYIDRVKQDLAEVEEIEDETRNTSPDDELDEAIYPYTYTGLSKI

AEEIEDETRDASRDDELVEAIYPNDYTGLSKI

>Trichogramma pretiosum_RYamide

MGSQNKTFNASTIQLIKWFGVSCLLVSVVASQVQENLHINERHGKPDMSMVPTFQIGSRYGRSPLPGKSVDRANRLLMMVPRVDRFYFGSRYGKRGLSSSSAAASPLVSHQQEEQQPSLHDRTTFDDFLNSYGLTEKQFNDIKRIVQEERWINNEGQAEISNDLYLDQI

>Argochrysis armilla_SIFamide

MVSTRQAFALIVILAVLVSTASAAYKKPPFNGSIFGKRSNTVSDYDIMNRALSQMCEAASETCNAWFSRQDAK

>Ceratosolen solmsi_SIFamide

MSAIRLMLVLLVIAAVSIFHVNAAYRKPPFNGSIFGKRANSISDYDIANRAMDAICEIARDNCNAWYAHQDSN

>Chrysis viridula_SIFamide_partial

...AAYKKPPFNGSIFGKRSNTVSDYDIMNRALSQMCEAASETCNAWFSRQDSK

>Copidosoma floridanum_SIFamide

MSAARFMFVLFVVVAVAILDVSSAAYRKPPFNGSIFGKRANTITDYDVASRAMDAICEIARDNCNAWYSHQDSN

>Fopius arisanus_SIFamide

MVSARFVSAILLIVIIATLSVDAAYRKPPFNGSIFGKRSSTGTDYEVINRALSNMCELASETCNAWMMHQDSN

>Microplitis demolitor_SIFamide

MMSLRVIIVLAIIAFIATVNVEGAYKKPPFNGSIFGKRSNSAADFEALSRALGNMCEIASETCNAWLMPQDTN

>Nasonia vitripennis_SIFamide

MSAARFALVLMVVLAVAILNVDAAYRKPPFNGSIFGKRANSVSDYDYASRAMDAICEIAKTNCNAYYGPQDSN

>Orussus abietinus_SIFamide

MASKDIVFALFAILVLVAVFGAEAAYRKPPFNGSIFGKRSSTITADYEVTGRALTTMCEVASETCNAWFAHQDSN

>Telenomus podisi_SIFamide

MNFVRNLLVVLVIFTMAVYIDGAYKKPPFNGSIFGKRSSSFVETDATSKALTAMCEVANDLCFTWYINPDSN

>Trichogramma pretiosum_SIFamide

MLSIRLTLFLLAIFVIMSVNANAAYRKPPFNGSIFGKRANSISVDYDMTNRAMDAICEIARDNCNTWYTRQDSI

>Argochrysis armilla_Short neuropeptide F

MLAKSCTMSIIVAIVIGTALATENYMDYGDDLSDKTSPGENLRELFRLLVQRNALDNAGFVGVPLEHFMSRKSQRSPSLRLRFGRSGPHVPAGILSRPMGDVATFDGN

>Ceratosolen solmsi_Short neuropeptide F

MAKLCTWMIVFFIVGATATVENYMDYGNDASGNDKTANLRELYRFLVQRSADEPEGFPDLASEHLQPRAVERSPSLRLRFGRSYPKYPRSPSLRLRFGRSYSNPVGPLSKLNEGGSSYTGFEEN

>Chrysis viridula_Short neuropeptide F

MLAKRCTMSIIIAVVIGMALATESYMDYGDELSDKTSPGENLRELYRLLVQRNALDNAGFVGVPLEHFMSRKSQRSPSLRLRFGRSGPHVPAGILSRPMTDVATFDGN-

>Diachasma alloeum_Short neuropeptide F

MMKYYGVTVIALFFVFGVAVSTENYMDYIADENADRDLEGLREFYRFLLRRNSMDNSYPSGSNIGSSNLVGIPYEHLMIRKSQRSPSLRLRFGRSGPAMPPDGMMTRLGSSSSGTEEN

>Fopius arisanus_Short neuropeptide F

MKYYGVTAIALFFVFGVAVSRENYMDYTADENVDRDLEDLREFYRFLVRHNSMDNSYPAGVNMAGSSLDIPYEHLMIRKSQRSPSLRLRFGRSGPAIPPEGLMSRLGSSSSGTEEN

>Ganaspis sp._Short neuropeptide F

MSFSRFVGILTCLILAISIVAATETYPDYADEASEKAPENILPELFRLWLERKAWENGFGIPSEHLMIRKSQRSPSLRLRFGRALPPIPVSIHQRYPFVRV

>Leptopilina boulardi_Short neuropeptide F

MNTFNNRFMGILTCLLAVICIIAATETYPDYADETAERIPENILPELYRRLLERKVWDNEYGIPMEHLMIRKSQRSPSLRLRFGRALPPIPTGALPRSNPGSFQEN

>Leptopilina clavipes_Short neuropeptide F

MNTMTFNNRFMGILTCFIAVICIAVATETYPDYADETADRIPENILPELYRRLMERKIWDNEYGIPMEHLMIRKSQRSPSLRLRFGRALPPIPTGAIPRSNPGSFQDN

>Leptopilina heterotoma_Short neuropeptide F

MNTMTFNNRFMGILTCFIAVICIAAATETYPDYADEMADRIPENILPELYRRLMERKVWDNEYGIPMEHLMIRKSQRSPSLRLRFGRALPPIPTGALPRSNPESVQDN

>Megastigmus spermotrophus_Short neuropeptide F

MMSKGCTWTIVLFFIVGAVAAVENYMDYGTDEATSNDKAANLRELYRILVQRSADDTDAFRGLGAEHLQPRAAERSPSLRLRFGRSYTKNPRSPSLRLRFGRSYSSPVDLLSRLNGSGSSSGALEEN

>Microplitis demolitor_Short neuropeptide F V1

MRSYSCAIVLFFIVGVVVAAENYLDYGEENADRNLENLREFYRYLLRRNSFDGSFRSSIPLDSPYEHLMIRKSQRSPSLRLRFGRSGPPAPQGNPITRPASNGESFEDN

>Microplitis demolitor_Short neuropeptide F V2

MRSYSCAIVLFFIVGVVVAAENYLDYGEENADRNLENLREFYRYLLRRNSFDGSFRSSIPLDSPYEHLMIRKSQRSPSLRLRFGRSGPPAPQVNLYPCRHISYIYISPYFITQ

>Nasonia giraulti_Short neuropeptide F

MIAKGCTWTIVFFVIVGVVSAVENYMDYGNESPNSEKSANLRELYRMLVQRSVDDSDSLRDLAEHLQPRAAERSPSLRLRFGRSYPKYPRSPSLRLRFGRSYPSQIGLLSRLNDGESSAAAFEEN

>Nasonia vitripennis_Short neuropeptide F

MIAKGCTWTIVFFVIVGVVSAVENYMDYGNESPNSEKSANLRELYRMLVQRSVDDSGSLRDLAEHLQPRAAERSPSLRLRFGRSYPKYPRSPSLRLRFGRSYPSQVGLLSRLNDGESSAAAFEEN

>Orussus abietinus_Short neuropeptide F

MMTKRCMGAIVFVTCLSIALAAESYKDYEAEDVAERQSENLNNLFRLYLQRNLVDNVGGIPIQHLMIRKSQRSPSYRLRFGRSNGLMPASSGMETSEYENN

>Pteromalus puparum_Short neuropeptide F

MIAKGCTWTIVFFVIVGVVSAVENYMDYGNESPNSDKSANLRELYRMLVQRSVDDSDSLRDLAEHLQPRAAERSPSLRLRFGRSYPKYPRSPSLRLRFGRSYPSQVGLLSRLNDAESSAAAFEEN

>Telenomus podisi_Short neuropeptide F

MRYVYLTCVIVVTINYIATSAESNFIENDASLSDSGEDVRLFIKRLDKRLREMENEKLMELLNDDHYMMRKSQRSPSLRLRFGRSSPNPDVLRILKLKNPVQYNQNQGY

>Trichogramma pretiosum_Short neuropeptide F

MITNHYTWAIVLLSVAGSFAAIENYMDYGNDGANGLESGNNNKPVNFRELYKFLVQHNPNEINWNDLMYMQQQEHLQPRAGLEQRSPSLRLRFGRSYSKYPVRRSPSLRLRFGRSYYPPSSPVGQGDDSTSAAASAAALEAN

>Argochrysis armilla_Sulfakinin_partial

...LDEEEEDLLENKRQQFDDYGHMRFGKREKFDDYGHMRFGRRHE

>Chrysis viridula_Sulfakinin

MNMNITFSCVMAAMWLLCRQSDAAPELVDSVHRRLHNRPVLRGYAIDELLDEEEEDLLENKRQQFDDYGHMRFGKRDKFDDYGHMRFGRRHD

>Argochrysis armilla_Tachykinin

MFISFSVIVVALSIRWSVAEETFTGNDGASAKRAPMGFQGMRGKKDIFPTKNADFSKTAVMGFEEMDSKREAMGPDIEDNLLHDDLEKRAPMGFQGMRGKKEYLGADFEESYQHDEYEKRAPMGFQGMRGKKALIEEYYKRAPMGFQGMRGKKSLEEVLEEIEKRAMMGFQGMRGKKTYYLDYPDEYDKRAMMMGFQGMRGKKDEFPADWEKRARMGFQGMRGKKSFLQDIEELEKRAIMGFHGMRGKKDSYDSYVDYAMDPYPYEKTGPLAFREFEDRIESEKRAPMGFQGMRGKRNTLHLFGSNSAMEKRANGYPGSQ

>Ceratosolen solmsi_Tachykinin_partial

...MASALAEHSAPQIVQKITRDLTHVESSKLPDPRFDLNKYQELHEANNKHLRVPDSYLERLLLERMEKRSSMRGFQGMRGKKNEFDLPPFFAVQIPESSYNGLGNYYNKRARMGFQGMRGKKDLEEMLNEMEKRSKMGFQGMRGKKSENYEEQEPQIIEEYRKRAPMGFQGMRGRKSSFLEELDELEKRALLGFHGMRGKKDIPYEREQIPMDIDGYVEKRPMMMGFHGMRGKRNTNYYEQQQ

>Copidosoma floridanum_Tachykinin

MAREGNVSSRAFAIAMMGLLVVLITSVRAQHETPSSAGHRGNDVGASEFSDAETKKRPAQDLSDLEKYHQMQELRDAGNLMRQLRELDEVLLGPSDYEKRASMRGFQGMRGKKSNEPYDGGARGYYYLDESMLDKRAPYSAFQGVRGKKSLEEVMMGEAEKRARMAGFQGMRGKKSEDFEDSDVEWHEKRAPSHGFQGMRGKKGMTLRDLYDDELVKRALSSFHGMRGKKDMTYEPPNDFDNYVDKRPMMMSFHGMRGKRSLPTKPEDAAGGGGSYKLEKRSPYRFFGSRGKKNPRWDSRAIKGFVGVRGKRLEQPKQLVSTWPSSADVYDELELAAVAAAAAEHNARSHLPRAD

>Diachasma alloeum_Tachykinin

MYVGLIVFVAVLIASQTLAEESKFNDVPSEKRAPMGFQGMRGKKDAENVLESDDFSKRAPMGFQGMRGKKDPAGPDLIDNIIQDDFEKRALMGFQGMRGKKDPLTLSDIKEALYHEEYDKRAPMGFQGMRGKKASEEELQFLDNIKRASMGFHGMRGKKLYADDYEDEYEKRALAMGFQGMRGKRDEYEGEWEKRAPRVFQGFQGMRGKKSFLEELEEMEKRAIMGFQGMRGKKDRLDGYYSGYVDPLDYEKRAPMGFQGMRGKKDGDKRASMGFVGMRGKRNAANIYGSDDNEFLDRLPADFLGSRFFDDEIHFVKRSGDEPVQFREKKIPNWELRGKFIGVRGKRWSPESLGSNENMMNEVLDHIERIGLNNDSTTKFDTQ

>Fopius arisanus_Tachykinin

MYVSSIIIVGVLIASPIFAEDSKLTDVLPEKRAPMGFQGMRGKKDMMSSLETEEFSKRAPMGFQGMRGKKDPTGPDLMDNIIQDEFEKGTPMDLQDLRRKKDLLTLPDIKEALYQEEYDKRAPMRYQGMRGNKELEEFLEEMKRASMGFHGMRGKKIYQDDYEDEYEKRALPMGFQGMRGKRDEYERGRNKRTSLYLPDIGHKSLMDRLENMKFKTFLELQEMDGQNDLFDGYYPEYADPDGYKKRAPMGFQGVRGKRDEDKRGSMGFVGMRGKRNVGGIYGAEDDEYREGLPADFLDSRYMSDDLQFNKRSARGVSGVRGKKIPRWEMRGTFIGVRGKRSPDSHDNMMKVLDDIDRINNDSTTKFDTQ

>Ganaspis sp._Tachykinin_partial

...MMGFQGMRGKKAIAEDEFNKRGPMGFQGVRGKKSLQEYMEELGREYVKRLKPEVFQSVKGKREDMDGGENYEEEWVKRALMGFQGMRGKKNSLDELQELQKRAIMGFQGVRGKRDLQHSFENADEAEIKYILRNLKMQ

>Leptopilina clavipes_Tachykinin

MFAGPIVFFVIIVTGSFAEESLSNDVSQEKRAPMGFQGMRGKKDLTSSENDEYSKRAPMGFQGMRGKKIIIGPDIEESFPFENSDKRAPMGFQGMRGKKEYLVPIFQDPYYNEDFEKRAAMMGFQGMRGKKNINEDEFNKRGPMGFQGVRGKKSFEEILEDMEKRARMPFHGTRGKKSYAYDYPDEFEKRALAMGFHGMRGKREDFNDEWEKRAPMGFQGMRGKKNSLDGIQELQKRAIMGFQGVRGKRDVEQSSGANSEGGKRKLLSIQEMQ

>Leptopilina heterotoma_Tachykinin

MFAGPIIFLVIIVTCAYAEESLSNDVSQEKRAPMGFQGMRGKKDLTSSENDDYSKRAPMGFQGMRGKKIIIGPDIEESFPFENSDKRAPMGFQGMRGKKEYLVPIFEDPYYNEDFEKRAAMMGFQGMRGKKNTNEDEFNKRGPMGFQGVRGKKSFEEILEDMEKRARMPFHGTRGKKSYAYDYPDEFEKRALAMGFHGMRGKREEFNDEWEKRAPMGFQGMRGKKNSFNDIQDLQKRAIMGFQVVNSIFIAIVIITQYILLINNSIK

>Megastigmus spermotrophus_Tachykinin

MYARNRMQLSVGVALLGLIGLALGEHSTSSGGAIAQKDDAEGKKREAQPEYAHDRDDLDKFQELHESSKHLRDPGSFLNRLISASDLEKRASMRGFQGMRGKKNEQMQVQLPQEPAMLYDPSIFYNLAPASAYDYYYDKRAPMGFQGVRGKKSYDDEYEKRAPMGFQGVRGKKDMEEVRPGRLFKVIEELEKRAMMGFQGMRGKKSEEYEEQQQQLQQQQEPQLESVAAEWQKRAPMGFQGVRGRKSSFLEELDDFEKRALLGFHGMRGKKDVPYERVQNEDAEGYVQEKRPMMMGFQGMRGKRSTNYYGSLEGGRYEKRSPYRFFGTRGKKNPRWEMRGKFVGVRGKKWTLTPSQSLVARIIEGLSYDDDDRERMQEVESERH

>Microplitis demolitor_Tachykinin

MYVSSIIVSVLLVTKIVAEDTAFNNNNNDININENDNVLKKRVAAMGFQGTRGKKDANNFDDDEFSKRAAMGFQGMRGKKDPTGPDSMDNLVDYEFEKRAPMGFQGMRGKKDEYLSSLTDDFNDGFYHDDEYDKRALMGFQGMRGKKDSPLEDEYYKRAMMGFQGMRGKKSIEEILDEIEKRAEYYGIPSDRMSLNDYIDDYEKRASAMGFQGMRGKKDEYENFEWEKRAPMGFQGMRGKKALLHELQELQKKAIMGFQGMRGKKNEENYYEYPVDSSEFGKRAPVGFQGMRGKKDGCKRDMRFVGMRGKRNVPGFYDINDYHDSPKSLDSRFIDTPIHFDKRSSYGFFGMRGKKIPRWEIRGKFVGVRGKK

>Nasonia vitripennis_Tachykinin

MTSPAAARLRATTMLLTLLTLGVSAQHSMTTTIRGQTDRDSEIILETKAPQQLPPQLAYVREPEELDGYQALDSTKHLREPSSSYLERLLLTEELDKRASMRGFQGMRGKKSVDPTSSFLAGYSSPEELQQYYEAYEHEKRAPMGFQGMRGKKSSADLEEDAYYKRAPMGFQGMRGKKSAIEEVLEELEKRAMMGGFQGMRGKKSPEPSVWNKRAPMGFQGMRGRKSSFLDELDELEKRALLGFHGMRGKKNGVAELAYEPADMDGYVEKRPMMMGFHGMRGKRSASFYGVGERYEKRSPYRFFGTRGKKNPRWEMRGKFVGVRGKKWSLAPRMPYDELLRH

>Orussus abietinus_Tachykinin

MFVGSSIILAVLIVPSLTEKTSSNDVLSLHDASLLLPEMRDRKELTSVERRAVSKRAPMGFQGMRGKKNVIGPDFADNFLHEDYEKRAPMGFQGMRGKKEYLSNQDDSSANEEYSNEKRAPMGFQGMRGKKSSFDEEYDKRAPMGFQGMRGKKSLEEVLDEIEKRAAMGFQGMRGKKNYYLEYPEDNEKRAMAMGFQGMRGKKDEWAADWEKRKPMGFQGMRGKKSFQDELDELEKRAIMGFQGMRGKKYYNDIDEFAEDYEKRAPKMGFQGMRGKKDIFDSDKSAPMSFQDTRGKKTISESYDDGVMDLSDYEKRALMGFQGMRGKKDSDKRAPMGFQGMRGKKDLDDPFGSTSEFSKRAPMGFQGMRGKKDENPIEYEPFSPFTYFDSRGKKTPRFAIRGKFVAVRGKKWSEGSLFDQSENLLDNIGRTGVNTDSTTTLDIQ

>Cotesia glomerata_Trissin

MNRLYMVIFIVVCALWNCGGILTCDQCGRECAPACGTRQFRACCYNNLRKRAPNLGFKLWLVPPENSEHFKFDYDS

>Cotesia rubecula_Trissin

MNRLYMVIFIVVCALWNCGGILTCDQCGRECAPACGTRQFRACCYNNLRKRAPNLGFK

LWLVPPENSEHFKFDYDS

>Cotesia vestalis_Trissin

MNRLYMVIFIVVCALWNCGGILTCDQCGRECAPACGTRQFRACCYNNLRKRAPNLGFKLWLVPPENSEHFKFDYDS

>Diachasma alloeum_Trissin

MSRLCIFVFLAVCALWTVGETVSCDQCGRECVTSCGTRQFRACCFNNLKKRAPNLGFRLWLAPPKESGHLQLVYDS

>Diachasmimorpha longicaudata_Trissin

MSRLCIFVFLAVCALWTVGETVSCDQCGRECVTSCGTRQFRACCFNNLKKRAPNLGLRLWLAPPKALDIS

>Microplitis demolitor_Trissin

MSRLYMIIFIAVYALWNCSGILTCDQCGRECTPICGTRQFRACCFNNLRKRAPNLGFKLWLVPSEETEHSKFYYDS

>Leptopilina clavipes_Trissin

MTREMGRTIYIILLIAVCALWTFGEALSCDQCGRECMKICGTRGFRACCFNNLKKRVPAVGIWVDPPKQTGHFQLI
