# Supplementary material for: In silico prediction of neuropeptides in Hymenoptera parasitoid wasps
Source: PLoS One. 2018 Feb 28;13(2):e0193561. doi: 10.1371/journal.pone.0193561 (PMC5831470; doi:10.1371/journal.pone.0193561)
Supplement: S2 Fig — Chrysidoidea sequences in phylogeny trees are indicated with blue circles; Ichneumonoidea sequences are indicated with light green squares; Chalcidoidea sequences are indicated with red triangles; Cynipoidea sequences are indicated with light blue rhombuses; Orussoidea with empty squares; Platygastroidea with empty rhombuses. Numbers above branches indicate phylogenies from amino acid sequences and only values above 50% are shown. The numbers of the paracopies carrying the motif are shown by the repeat numbers, and the numbers in parentheses means the numbers of the paracopies predicted from a partial precursor. Identities in alignments are highlighted in dark (100%) and in grey (80%~100%). (PPTX) [file pone.0193561.s003.pptx]

## Slide 1
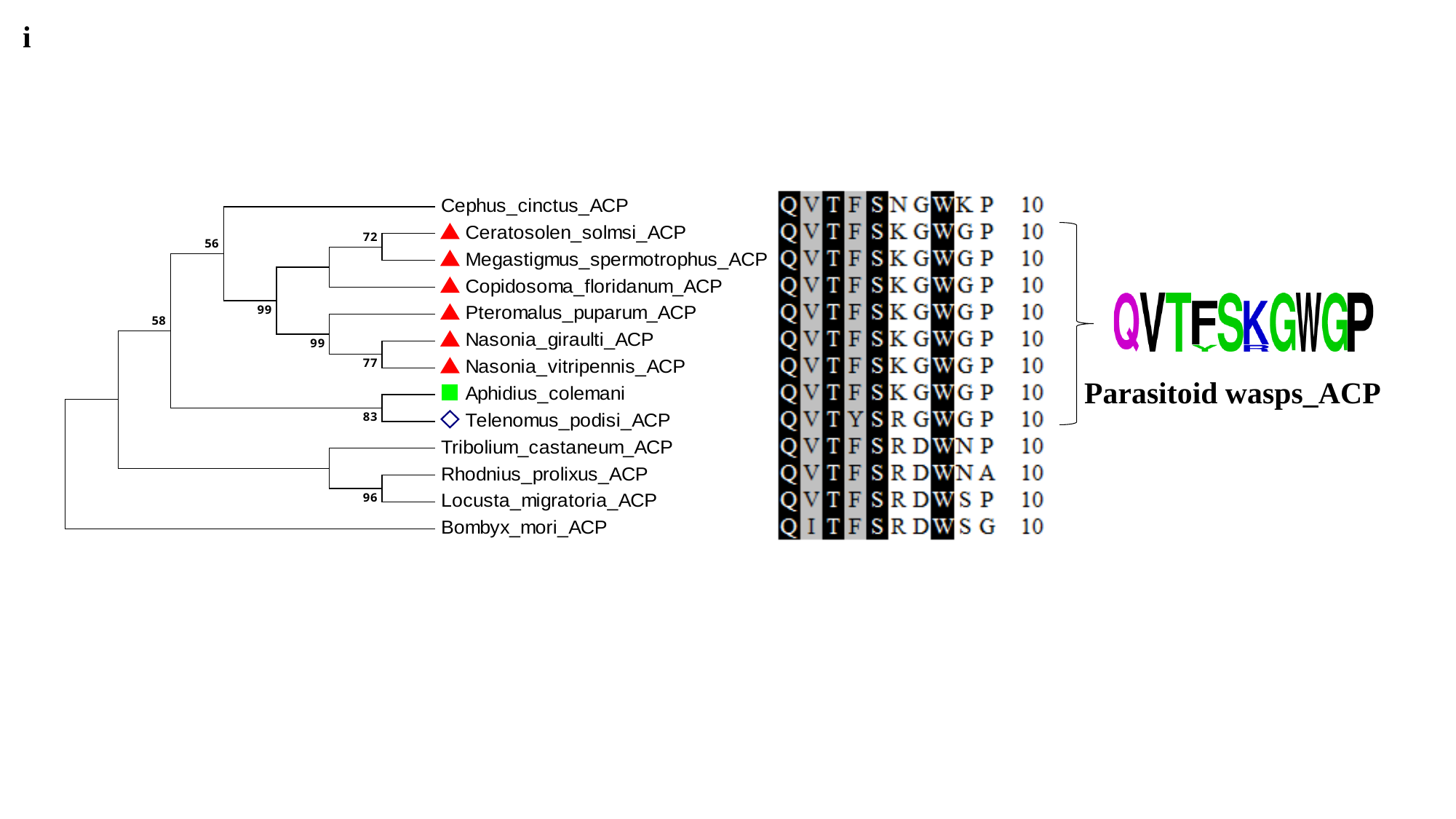

i
Parasitoid wasps_ACP

## Slide 2
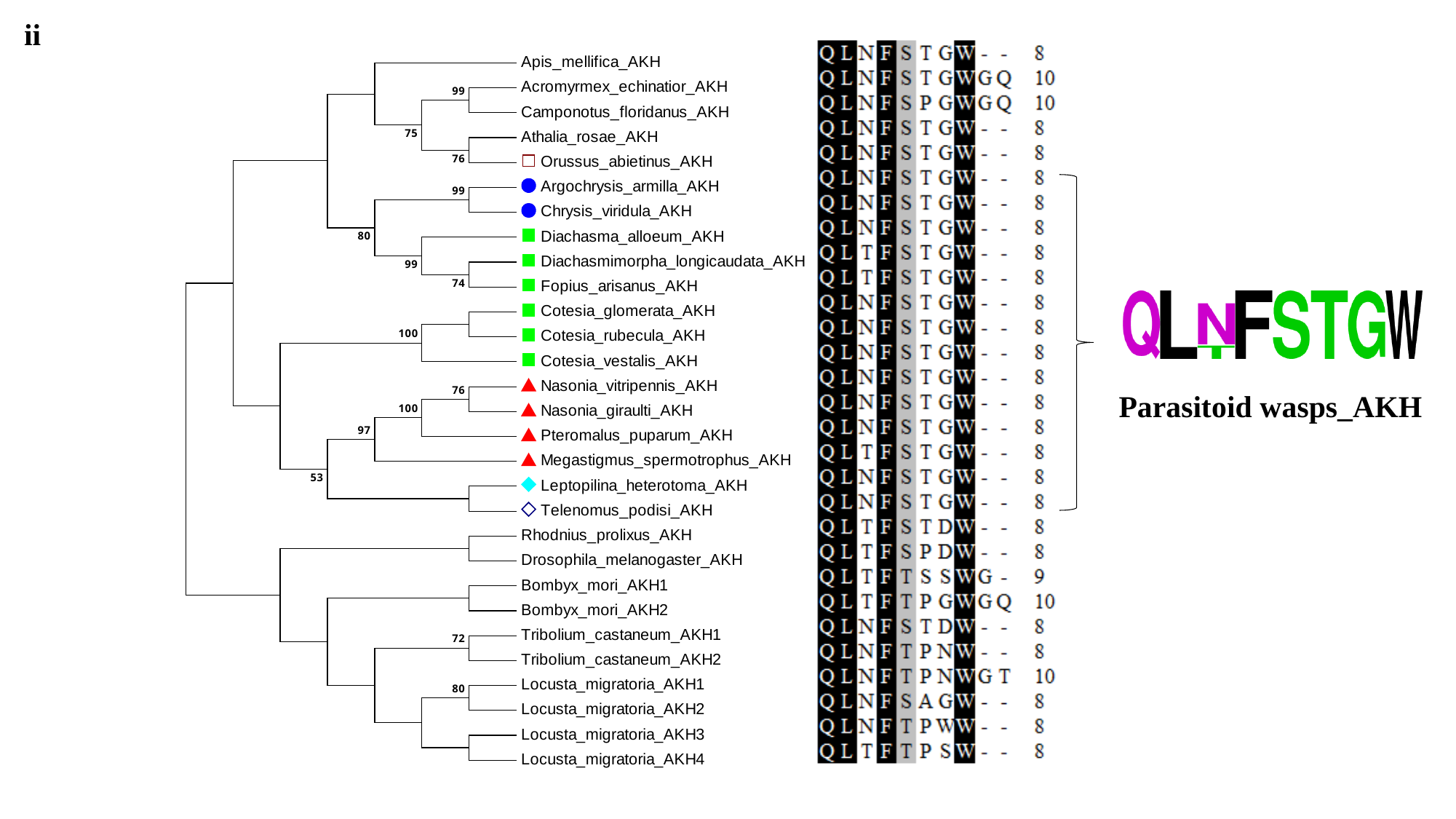

ii
Parasitoid wasps_AKH

## Slide 3
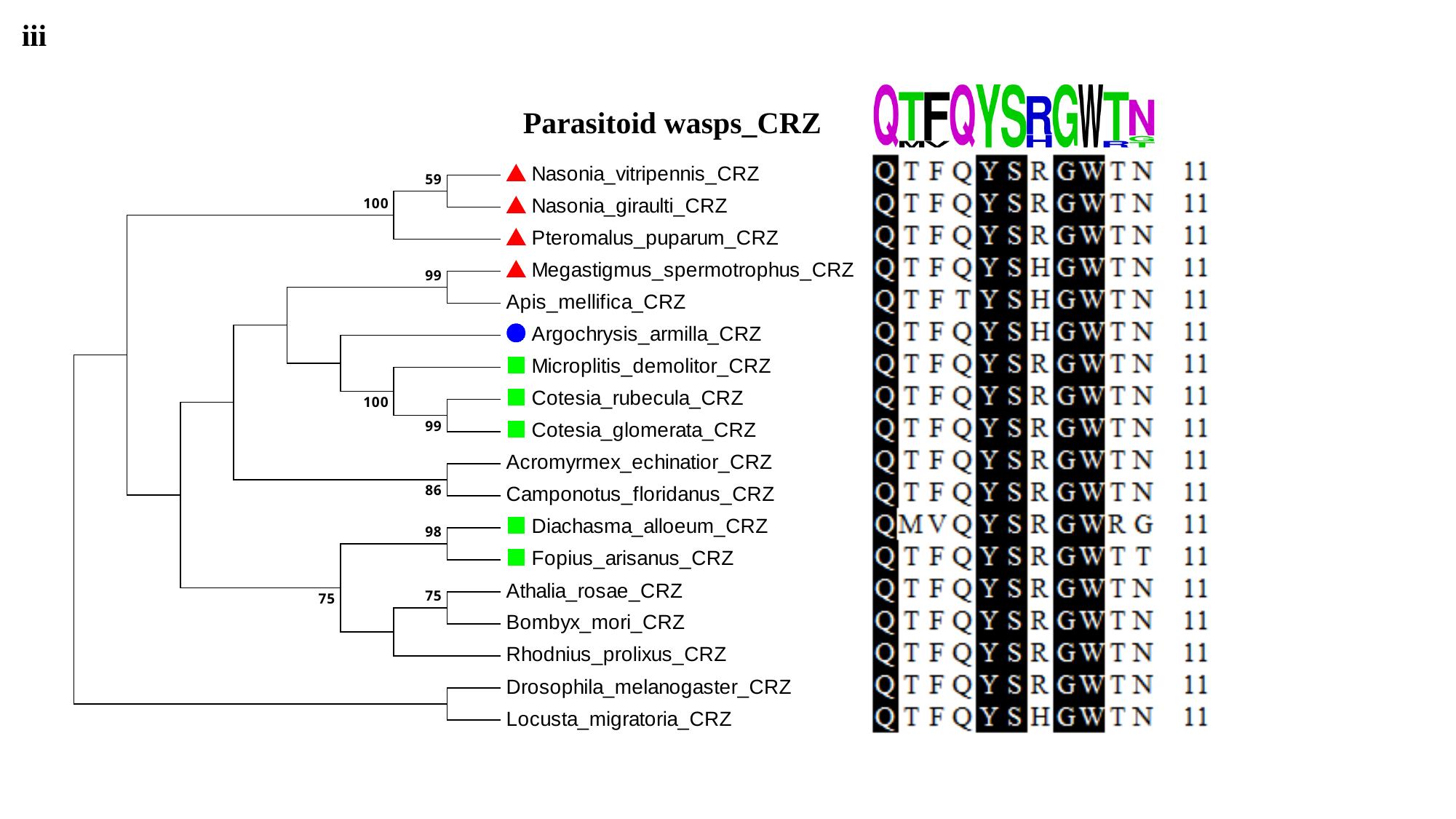

iii
Parasitoid wasps_CRZ

## Slide 4
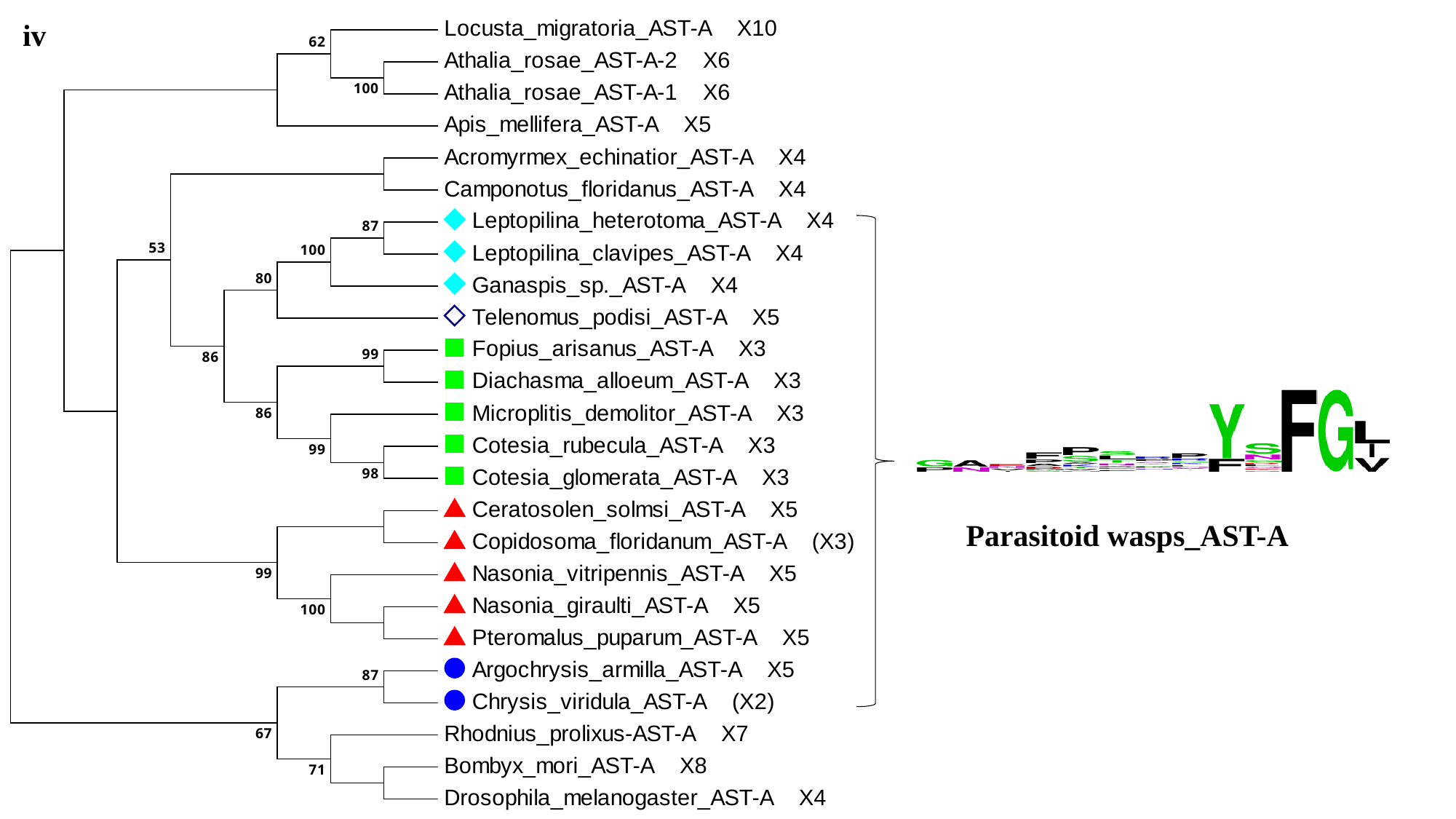

iv
Parasitoid wasps_AST-A

## Slide 5
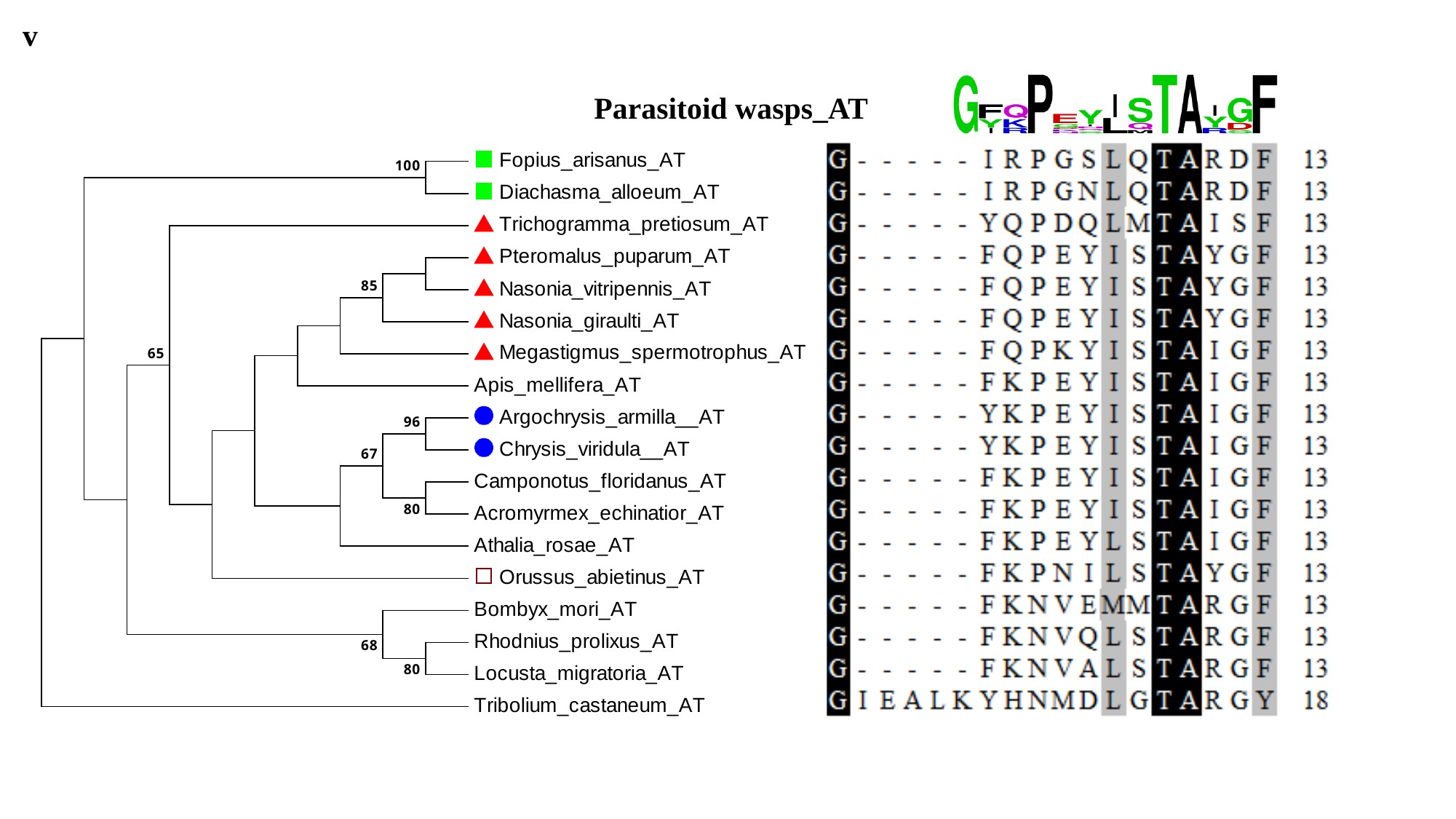

v
Parasitoid wasps_AT

## Slide 6
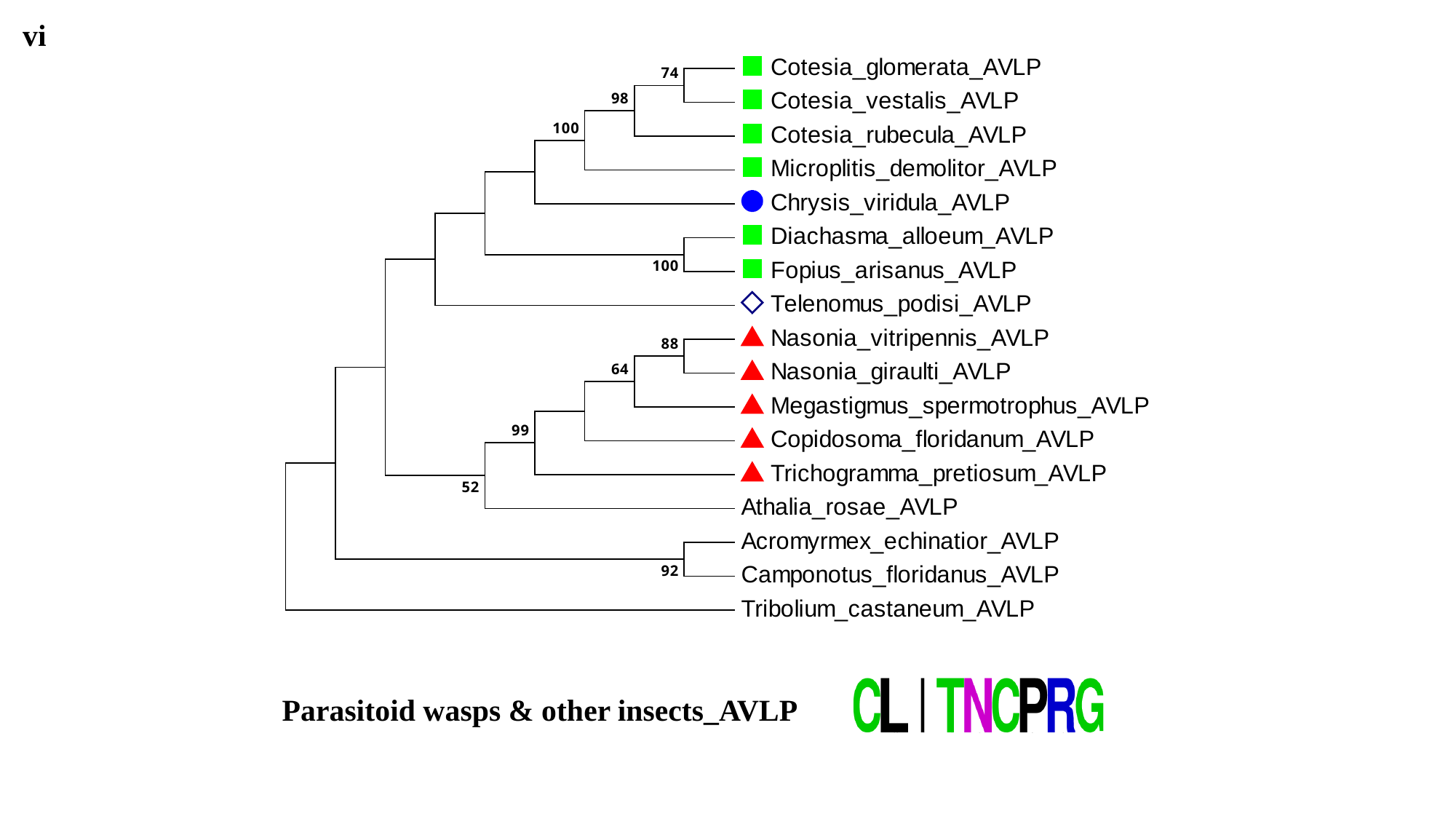

vi
Parasitoid wasps & other insects_AVLP

## Slide 7
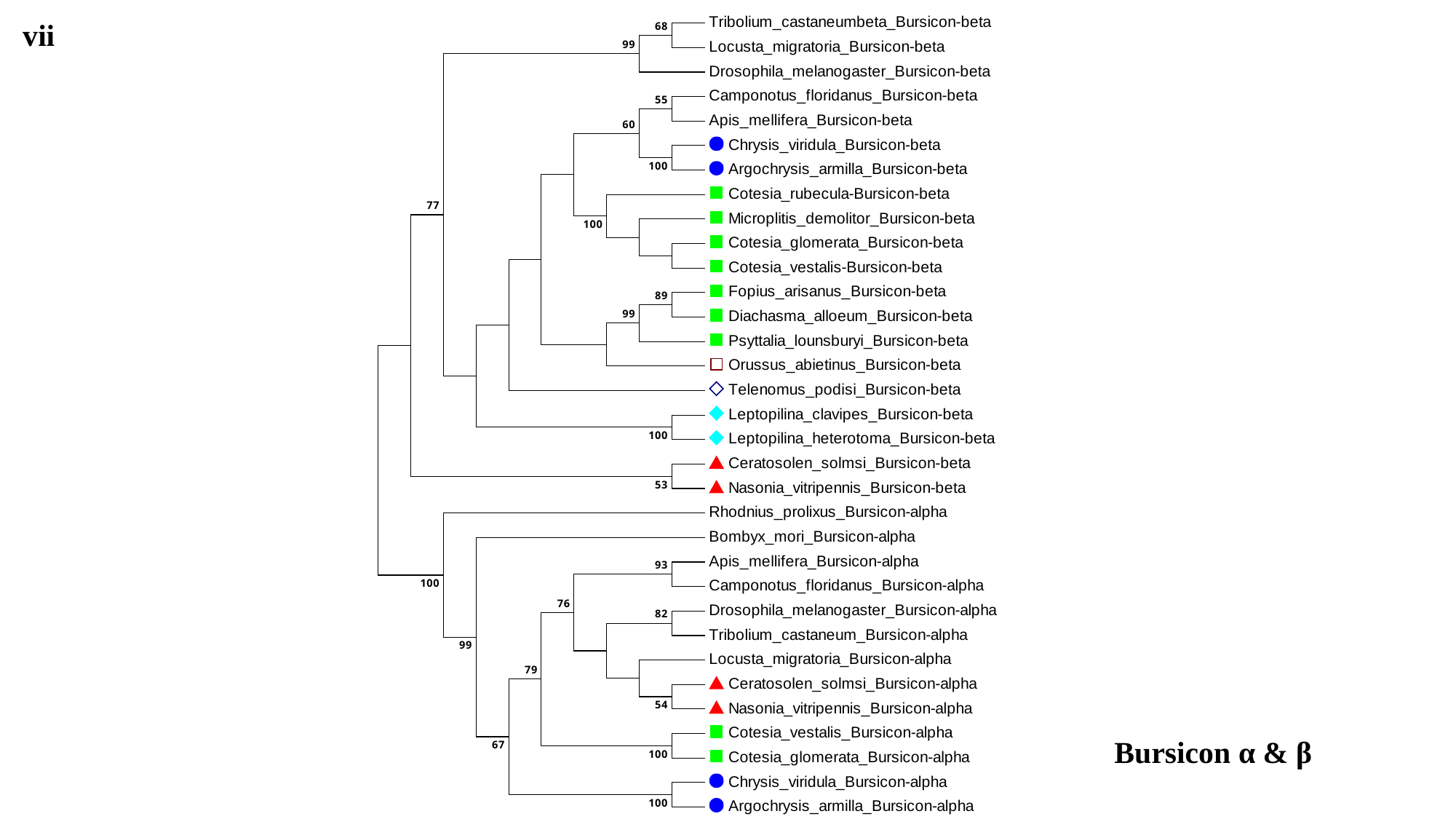

vii
Bursicon α & β

## Slide 8
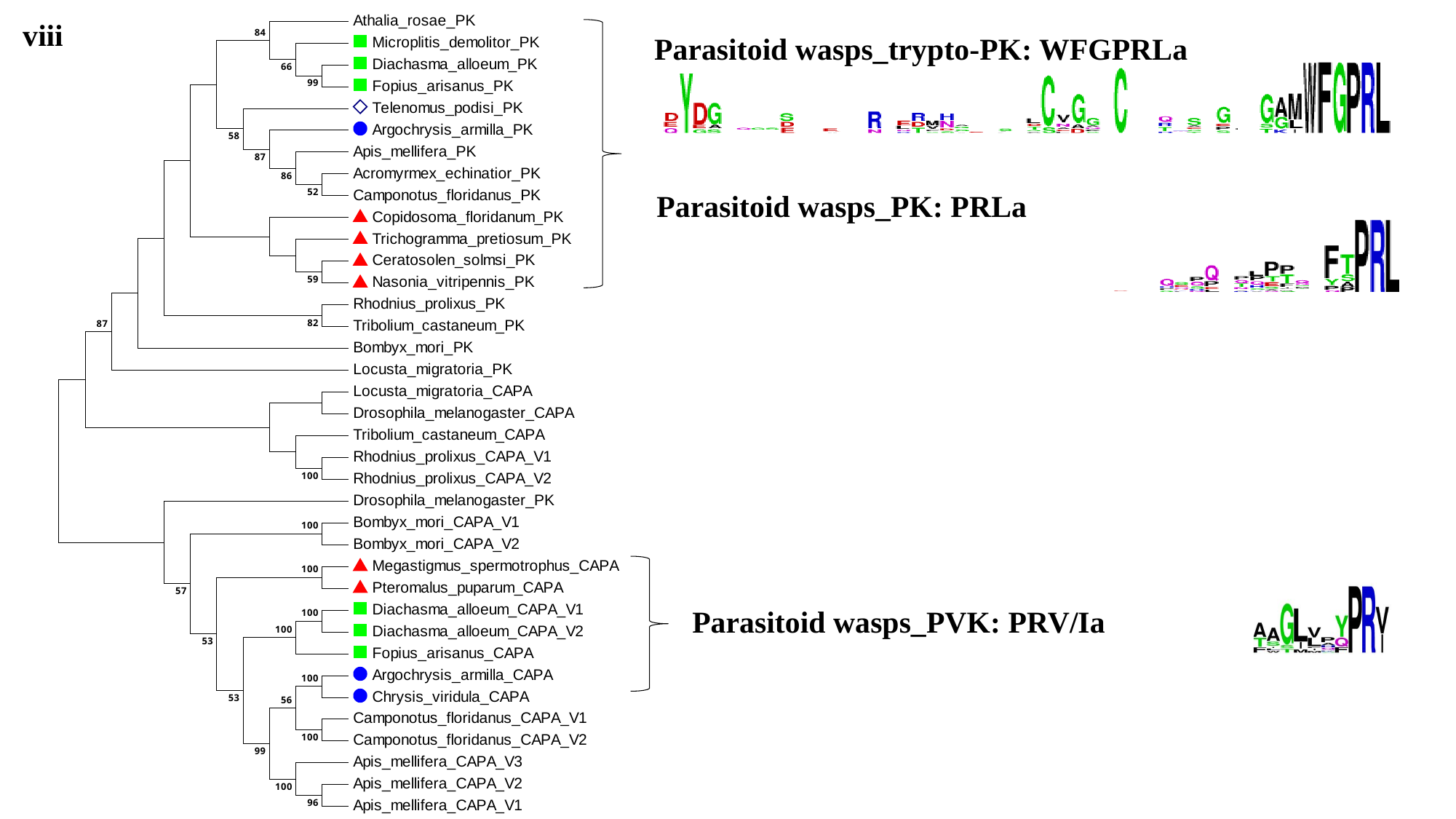

viii
Parasitoid wasps_trypto-PK: WFGPRLa
Parasitoid wasps_PK: PRLa
Parasitoid wasps_PVK: PRV/Ia

## Slide 9
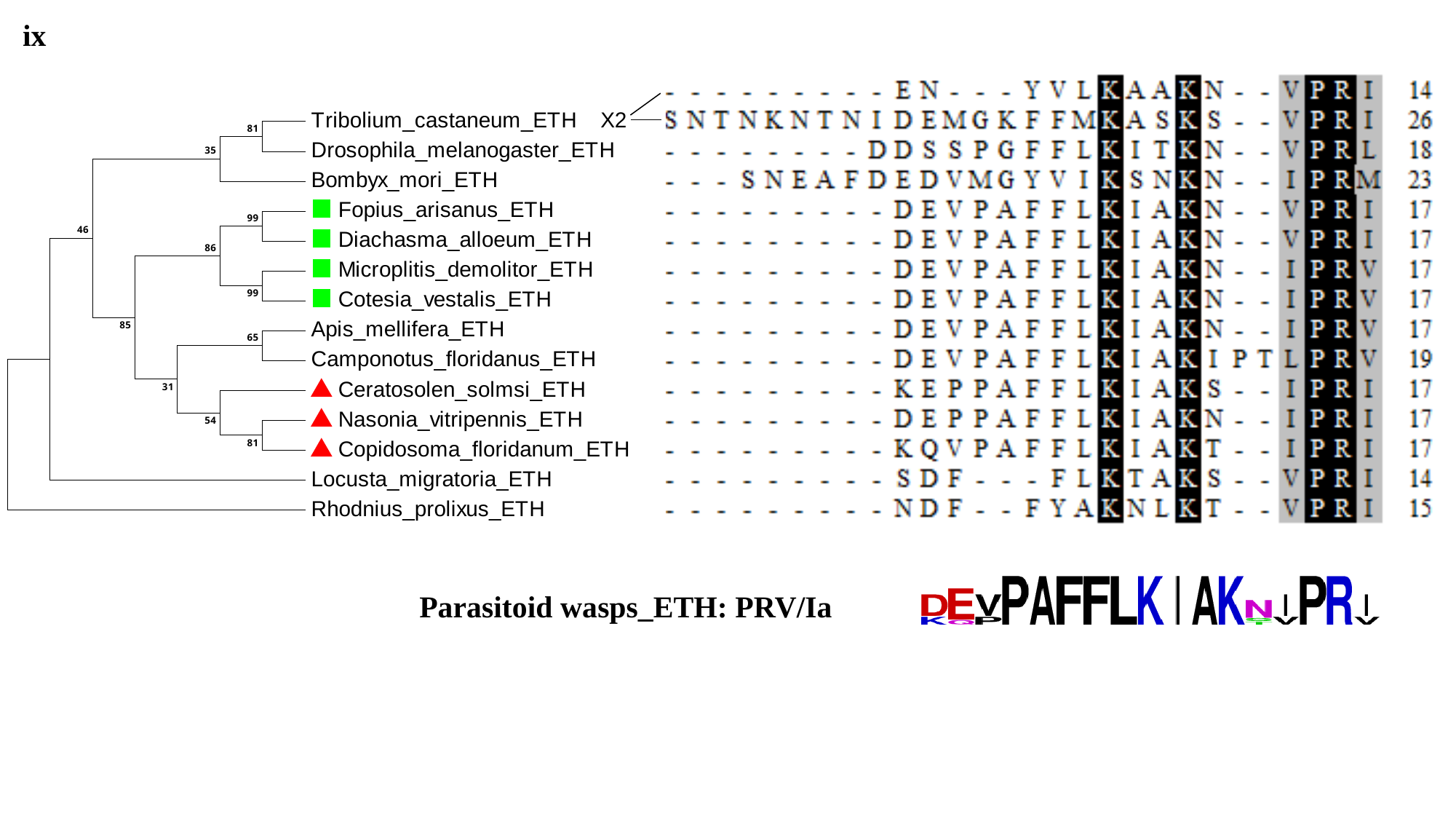

ix
Parasitoid wasps_ETH: PRV/Ia

## Slide 10
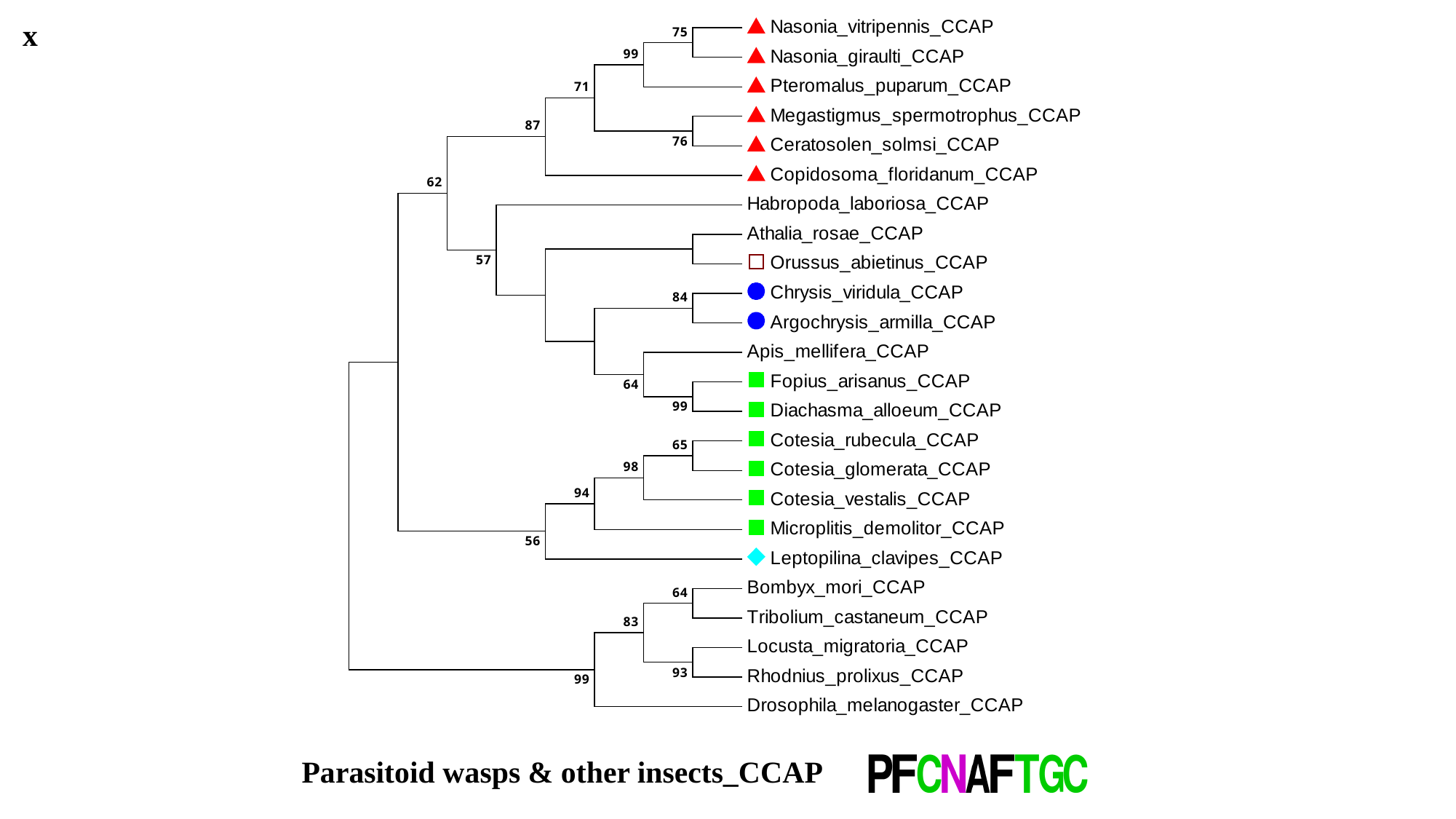

x
Parasitoid wasps & other insects_CCAP

## Slide 11
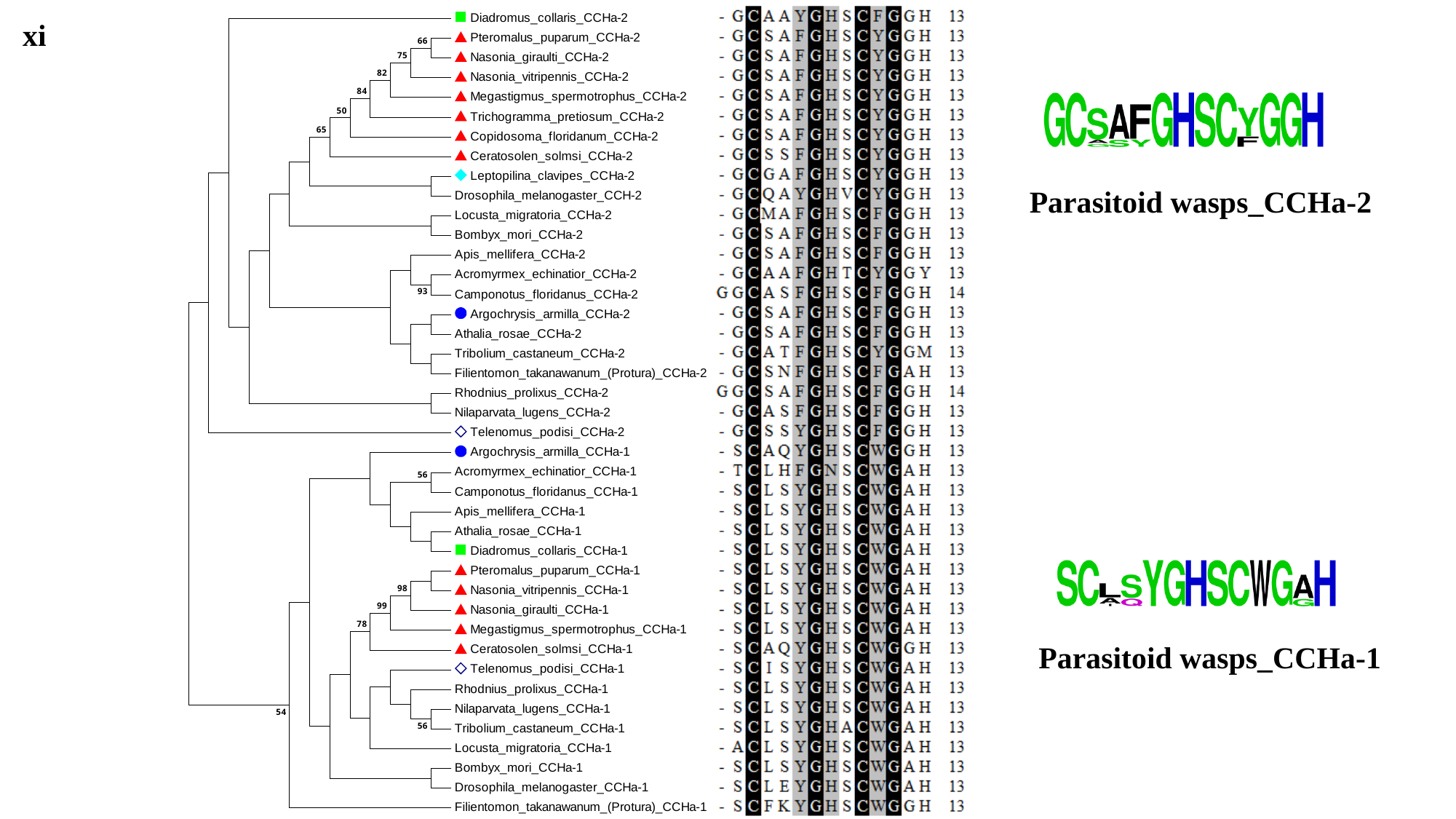

xi
Parasitoid wasps_CCHa-2
Parasitoid wasps_CCHa-1

## Slide 12
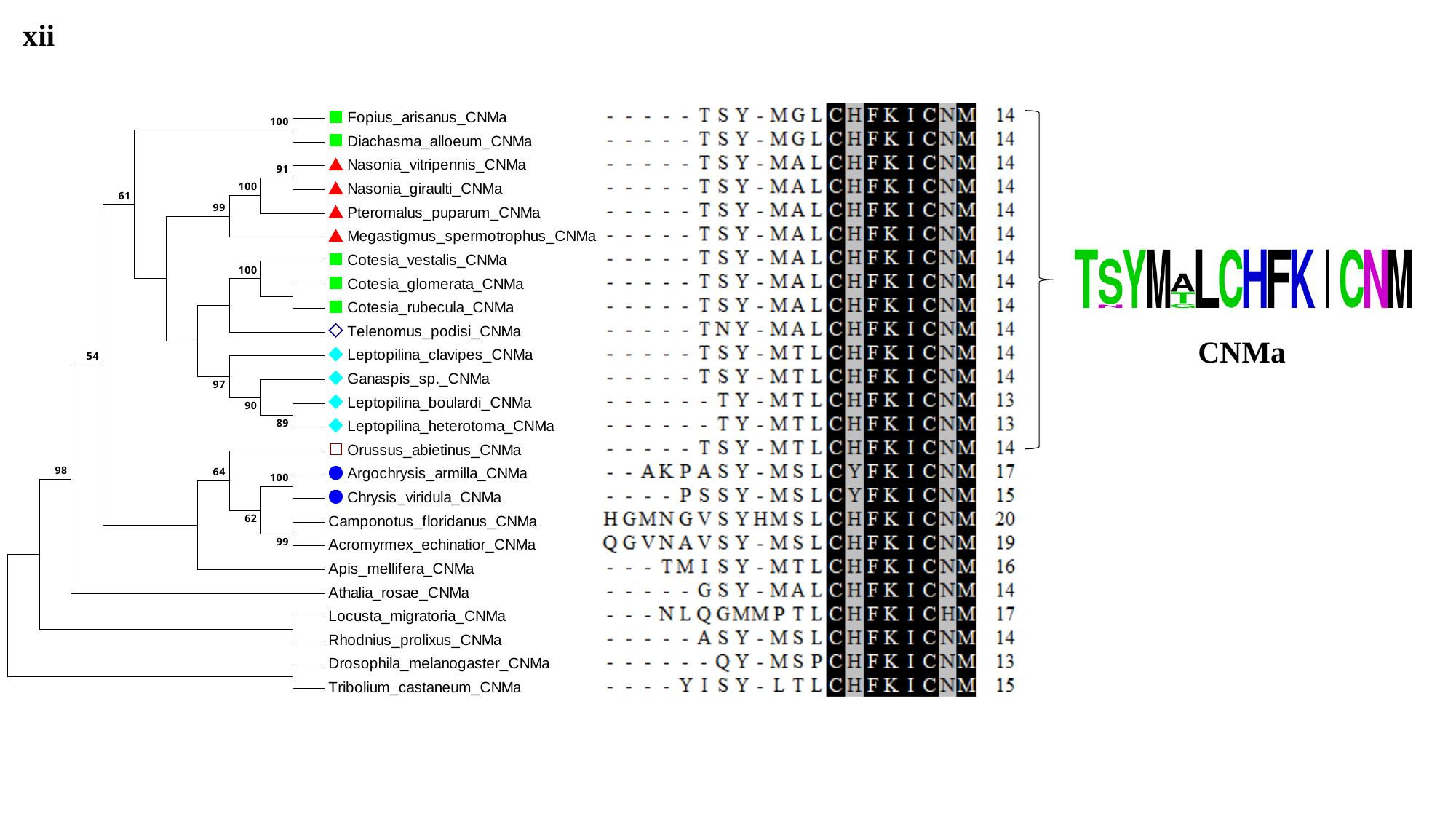

xii
CNMa

## Slide 13
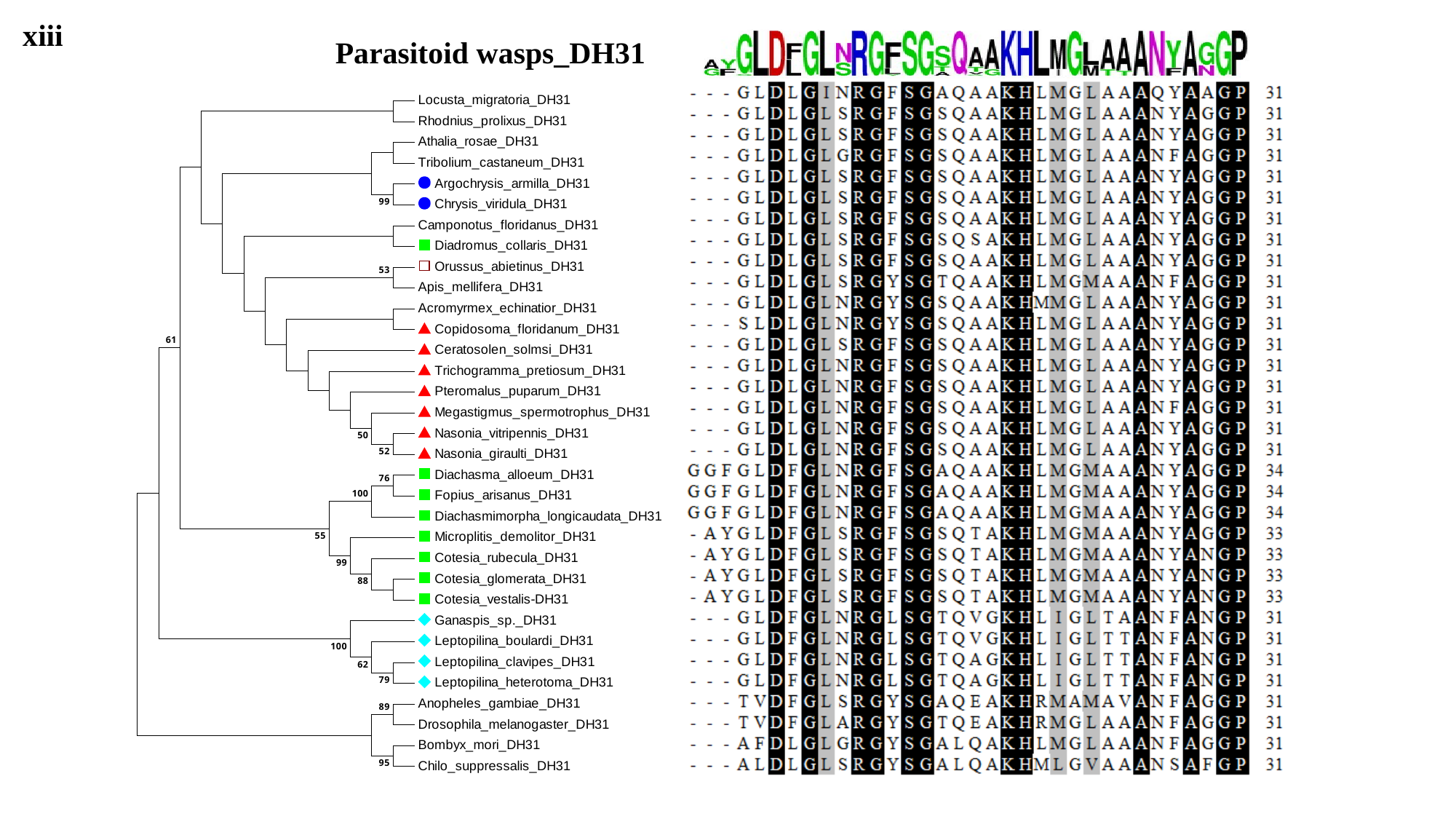

xiii
Parasitoid wasps_DH31

## Slide 14
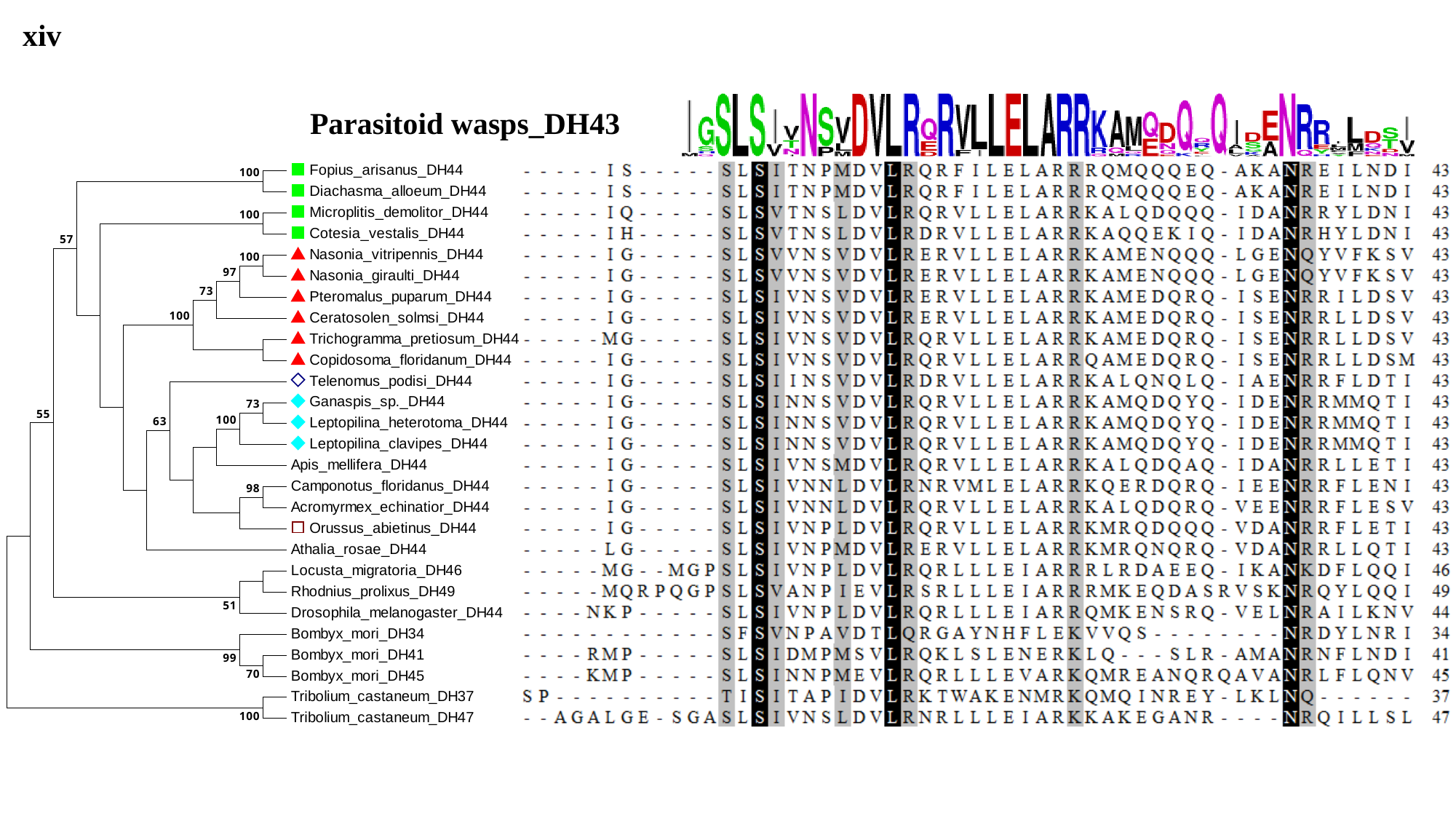

xiv
Parasitoid wasps_DH43

## Slide 15
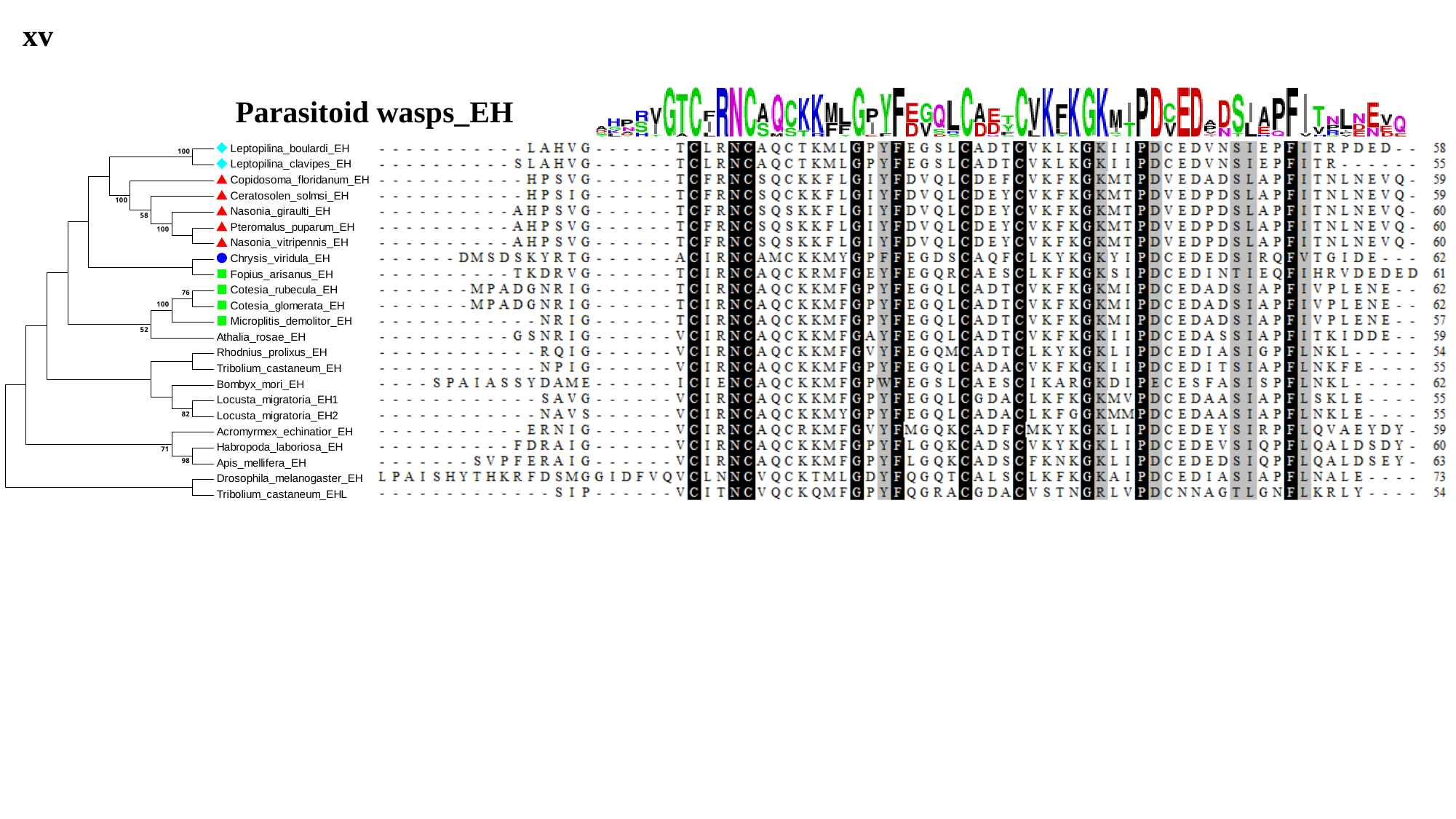

xv
Parasitoid wasps_EH

## Slide 16
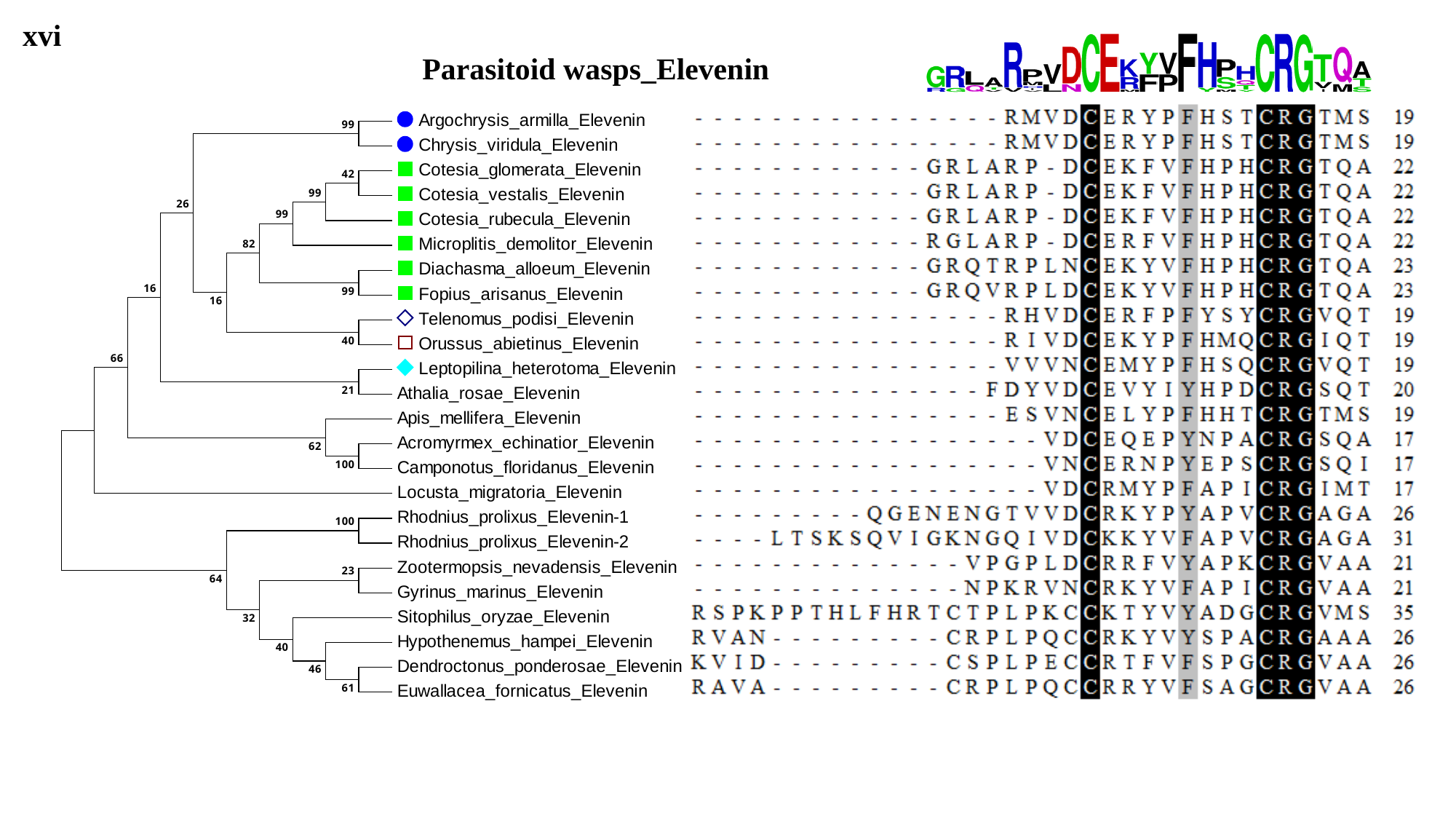

xvi
Parasitoid wasps_Elevenin

## Slide 17
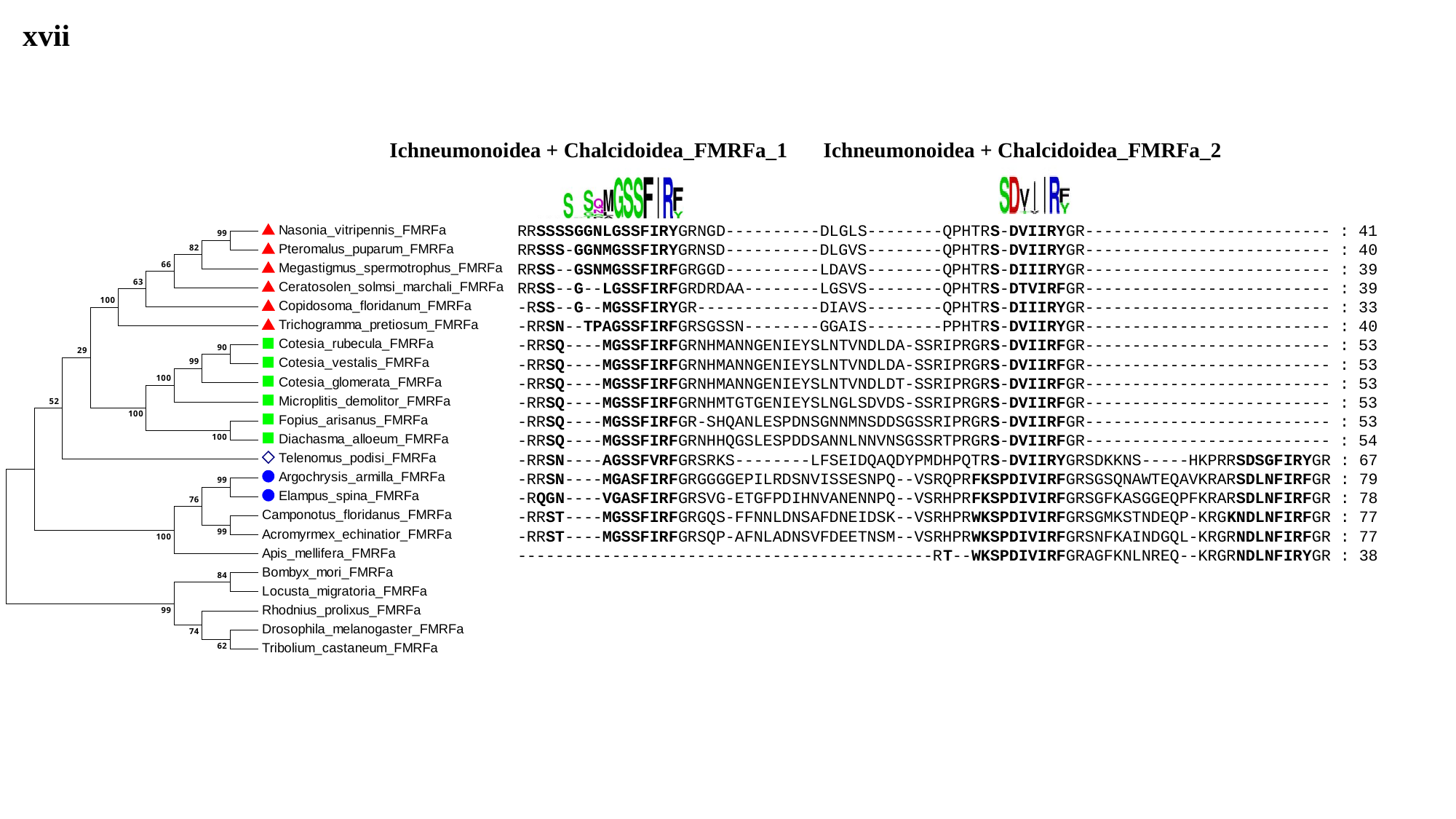

xvii
Ichneumonoidea + Chalcidoidea_FMRFa_1
Ichneumonoidea + Chalcidoidea_FMRFa_2
RRSSSSGGNLGSSFIRYGRNGD----------DLGLS--------QPHTRS-DVIIRYGR-------------------------- : 41
RRSSS-GGNMGSSFIRYGRNSD----------DLGVS--------QPHTRS-DVIIRYGR-------------------------- : 40
RRSS--GSNMGSSFIRFGRGGD----------LDAVS--------QPHTRS-DIIIRYGR-------------------------- : 39
RRSS--G--LGSSFIRFGRDRDAA--------LGSVS--------QPHTRS-DTVIRFGR-------------------------- : 39
-RSS--G--MGSSFIRYGR-------------DIAVS--------QPHTRS-DIIIRYGR-------------------------- : 33
-RRSN--TPAGSSFIRFGRSGSSN--------GGAIS--------PPHTRS-DVIIRYGR-------------------------- : 40
-RRSQ----MGSSFIRFGRNHMANNGENIEYSLNTVNDLDA-SSRIPRGRS-DVIIRFGR-------------------------- : 53
-RRSQ----MGSSFIRFGRNHMANNGENIEYSLNTVNDLDA-SSRIPRGRS-DVIIRFGR-------------------------- : 53
-RRSQ----MGSSFIRFGRNHMANNGENIEYSLNTVNDLDT-SSRIPRGRS-DVIIRFGR-------------------------- : 53
-RRSQ----MGSSFIRFGRNHMTGTGENIEYSLNGLSDVDS-SSRIPRGRS-DVIIRFGR-------------------------- : 53
-RRSQ----MGSSFIRFGR-SHQANLESPDNSGNNMNSDDSGSSRIPRGRS-DVIIRFGR-------------------------- : 53
-RRSQ----MGSSFIRFGRNHHQGSLESPDDSANNLNNVNSGSSRTPRGRS-DVIIRFGR-------------------------- : 54
-RRSN----AGSSFVRFGRSRKS--------LFSEIDQAQDYPMDHPQTRS-DVIIRYGRSDKKNS-----HKPRRSDSGFIRYGR : 67
-RRSN----MGASFIRFGRGGGGEPILRDSNVISSESNPQ--VSRQPRFKSPDIVIRFGRSGSQNAWTEQAVKRARSDLNFIRFGR : 79
-RQGN----VGASFIRFGRSVG-ETGFPDIHNVANENNPQ--VSRHPRFKSPDIVIRFGRSGFKASGGEQPFKRARSDLNFIRFGR : 78
-RRST----MGSSFIRFGRGQS-FFNNLDNSAFDNEIDSK--VSRHPRWKSPDIVIRFGRSGMKSTNDEQP-KRGKNDLNFIRFGR : 77
-RRST----MGSSFIRFGRSQP-AFNLADNSVFDEETNSM--VSRHPRWKSPDIVIRFGRSNFKAINDGQL-KRGRNDLNFIRFGR : 77
--------------------------------------------RT--WKSPDIVIRFGRAGFKNLNREQ--KRGRNDLNFIRYGR : 38

## Slide 18
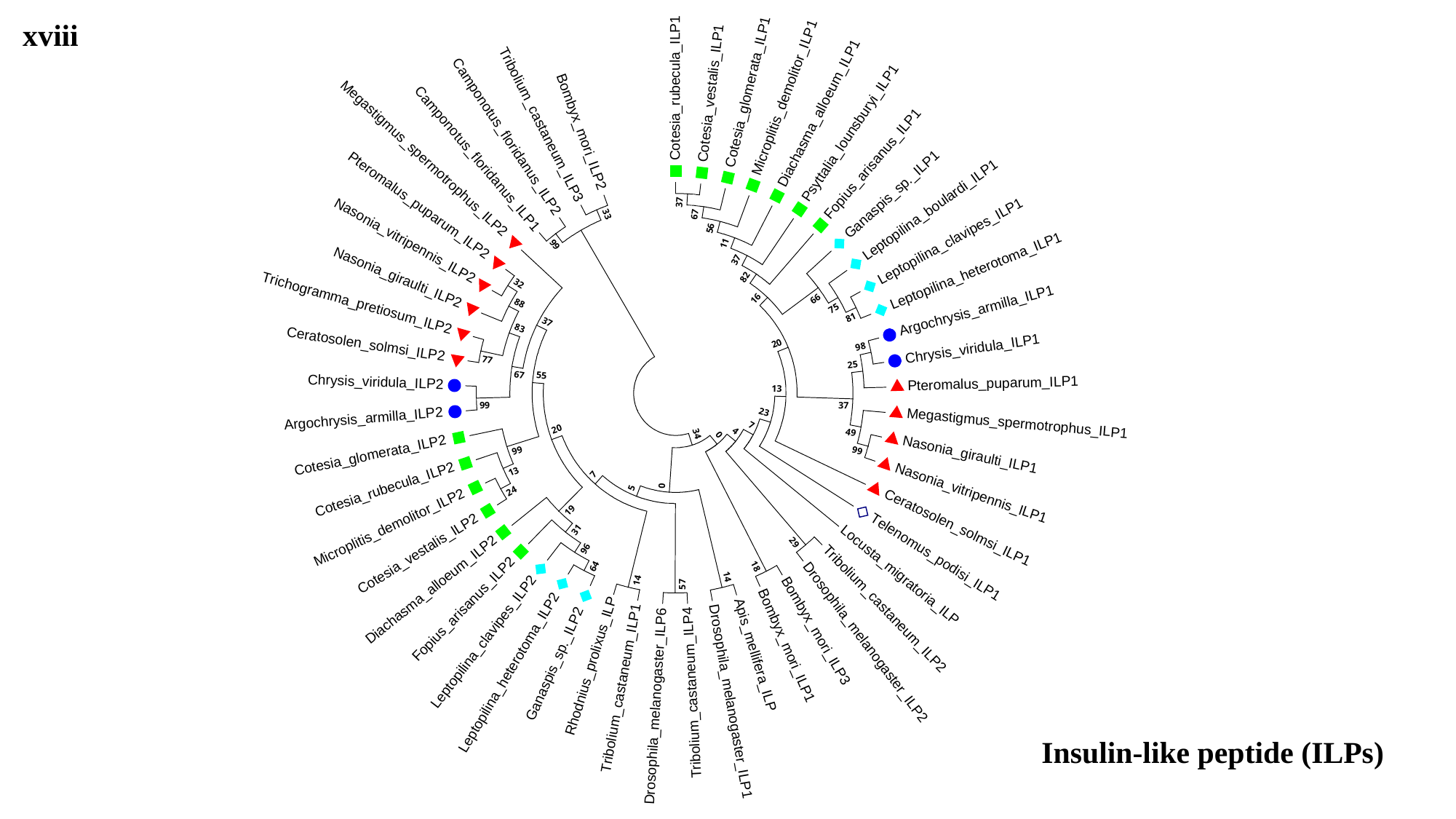

xviii
Insulin-like peptide (ILPs)

## Slide 19
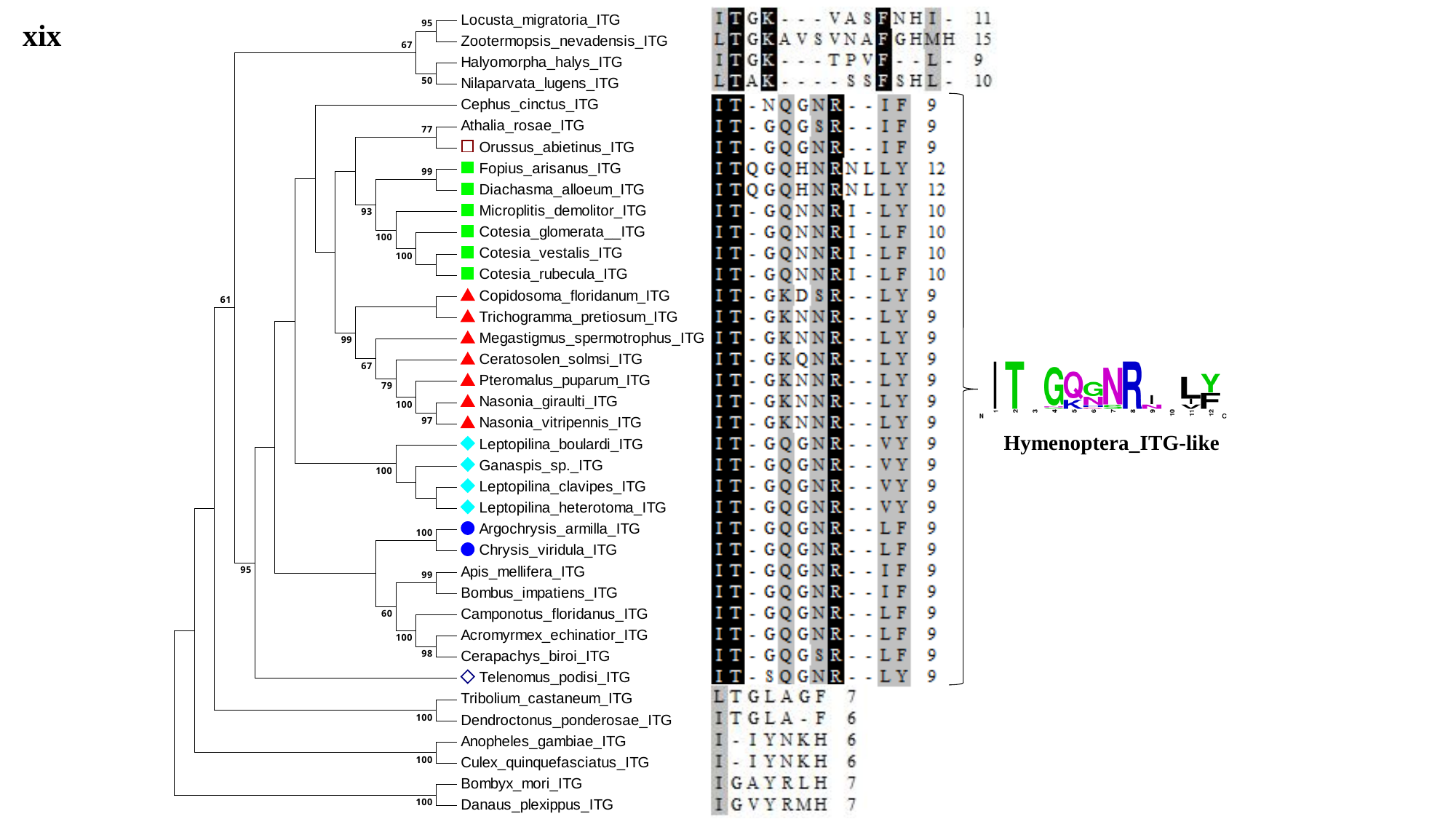

xix
Hymenoptera_ITG-like

## Slide 20
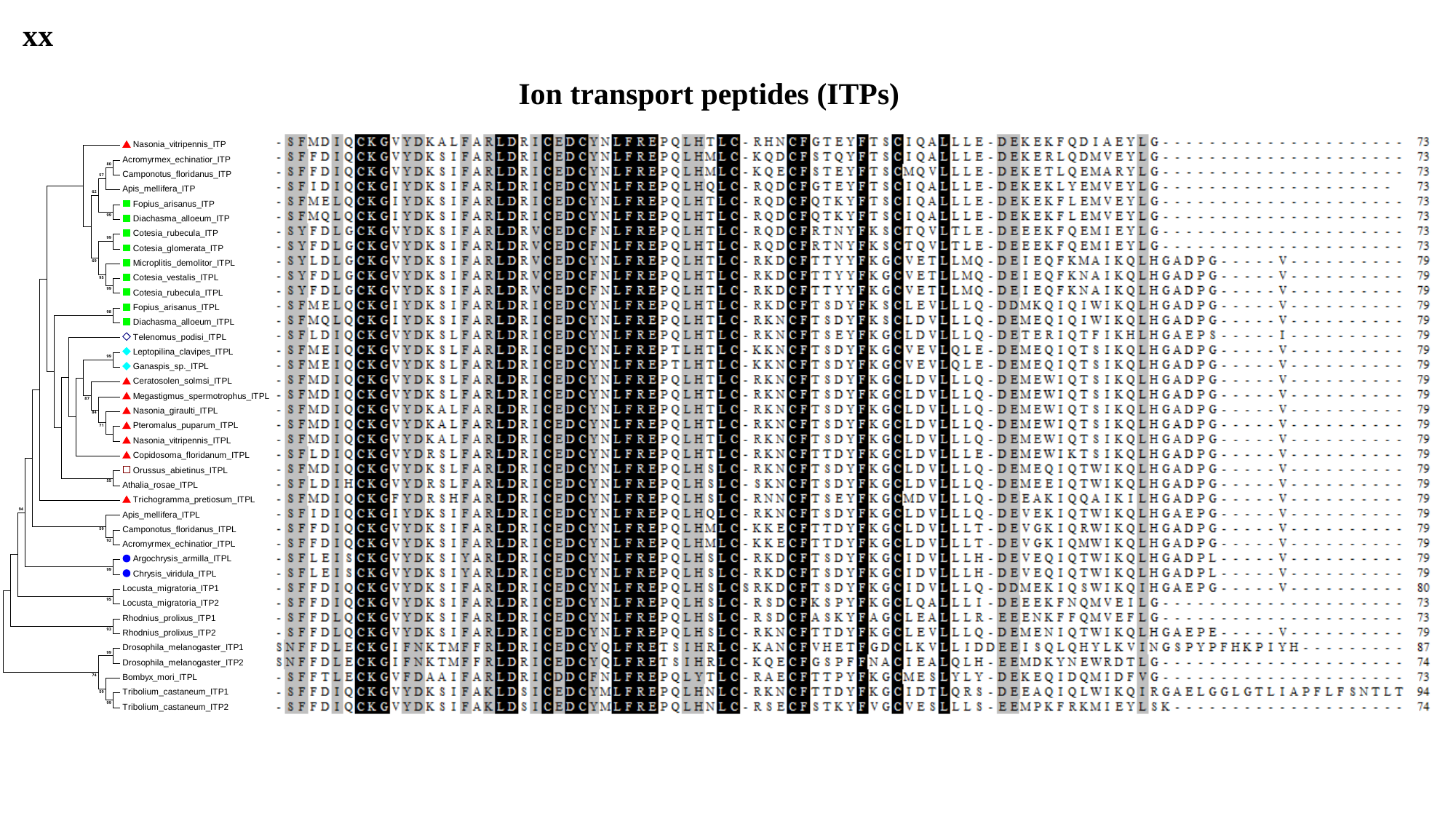

xx
Ion transport peptides (ITPs)

## Slide 21
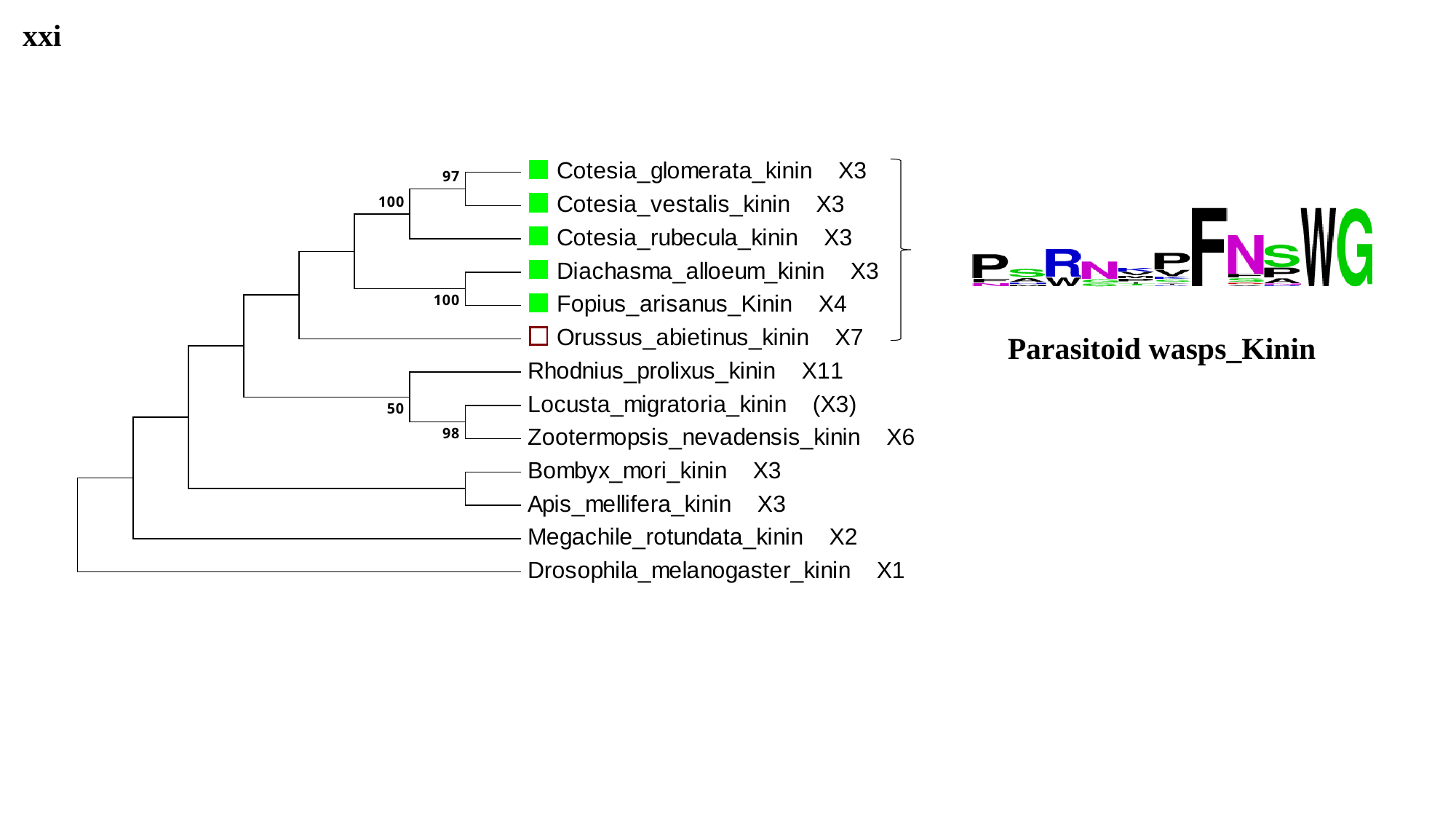

xxi
Parasitoid wasps_Kinin

## Slide 22
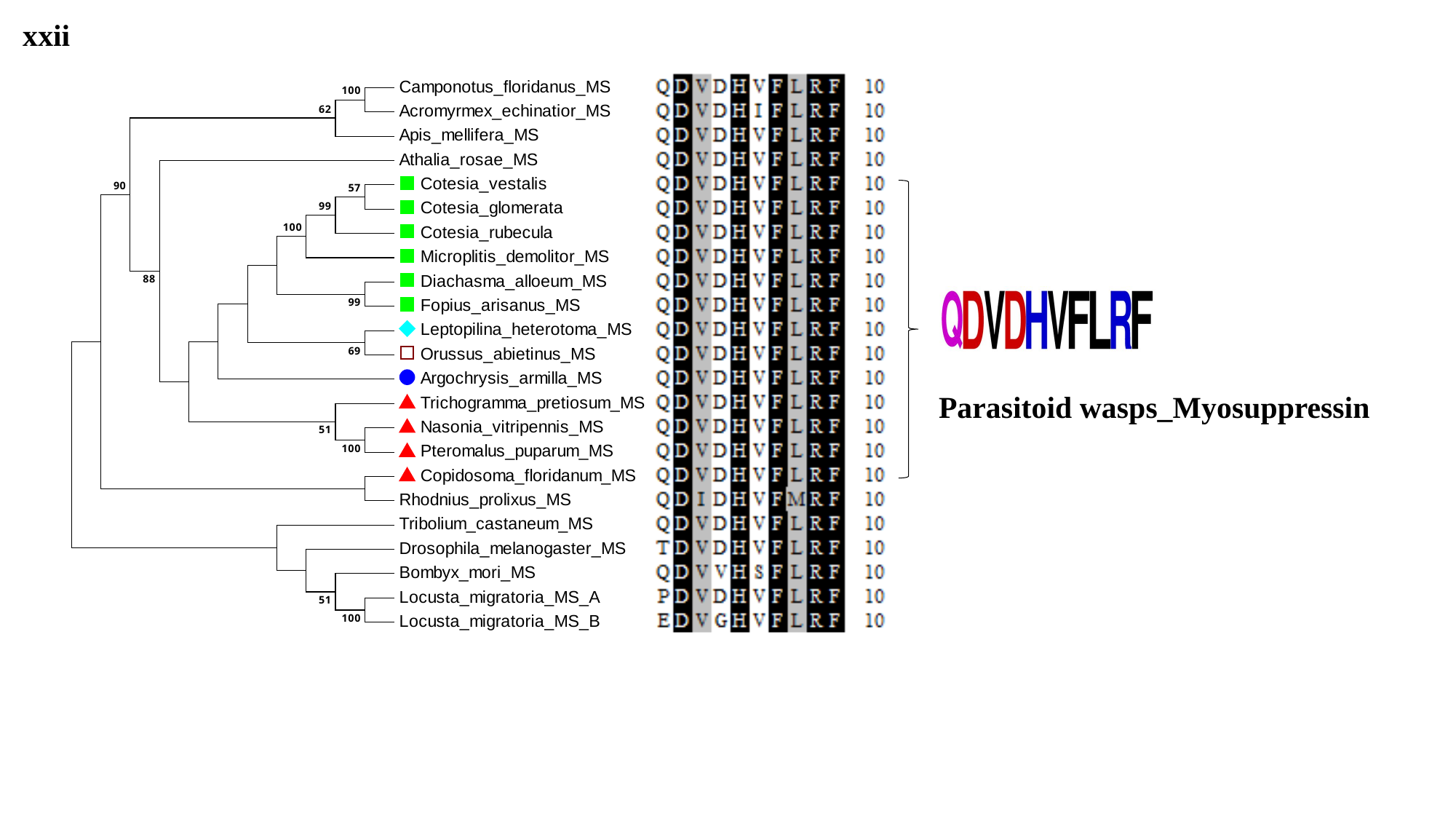

xxii
Parasitoid wasps_Myosuppressin

## Slide 23
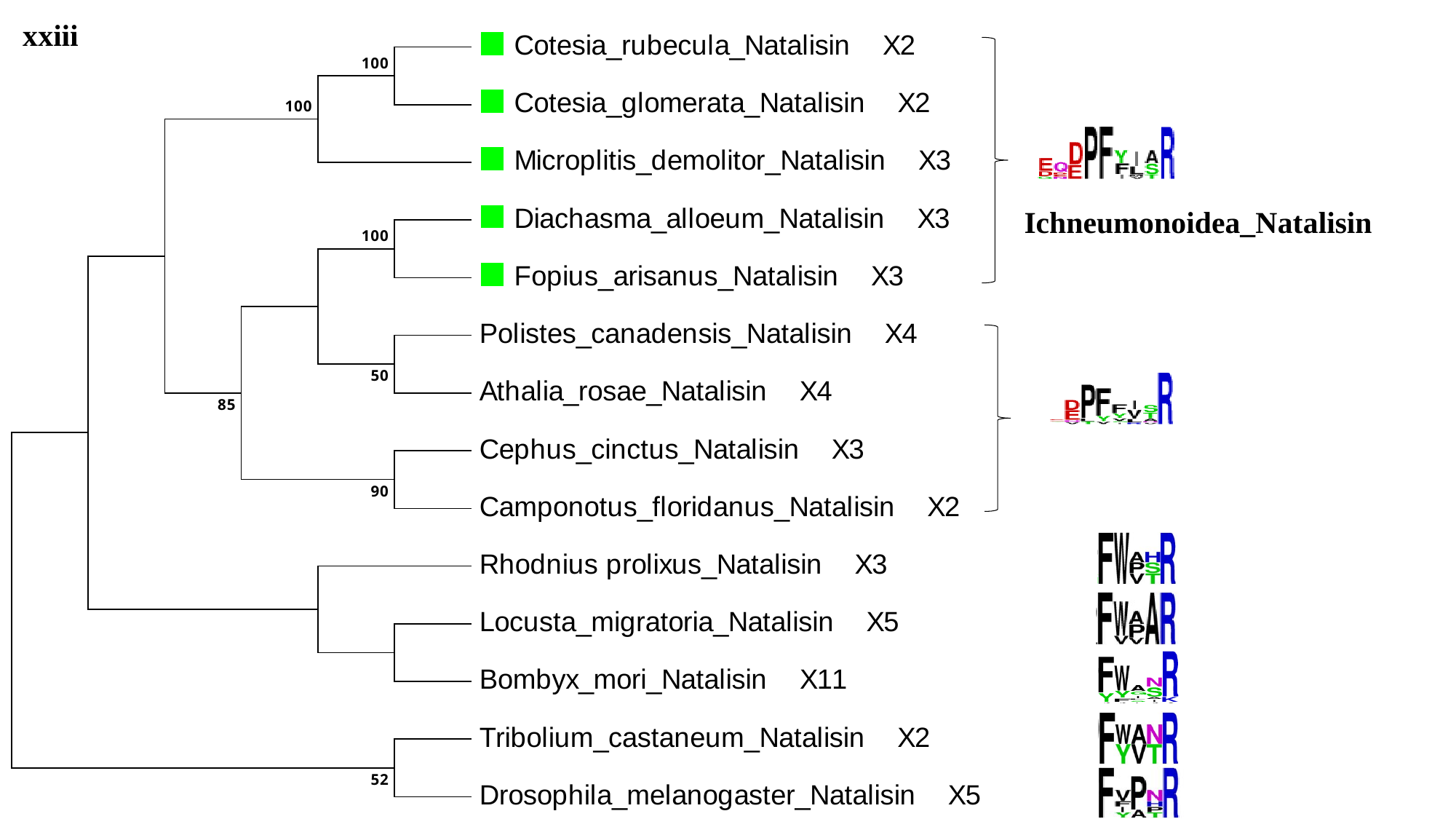

xxiii
Ichneumonoidea_Natalisin

## Slide 24
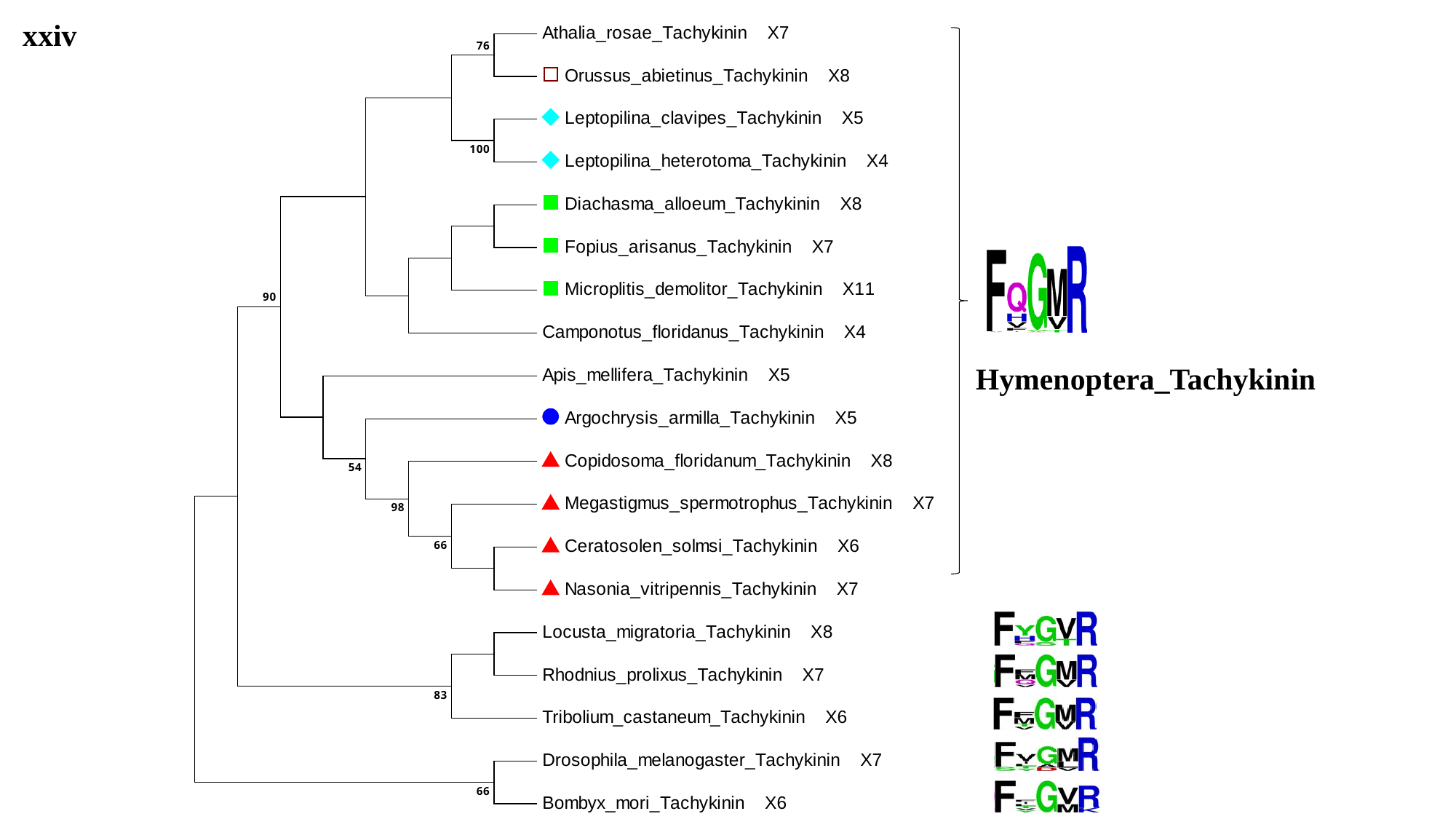

xxiv
Hymenoptera_Tachykinin

## Slide 25
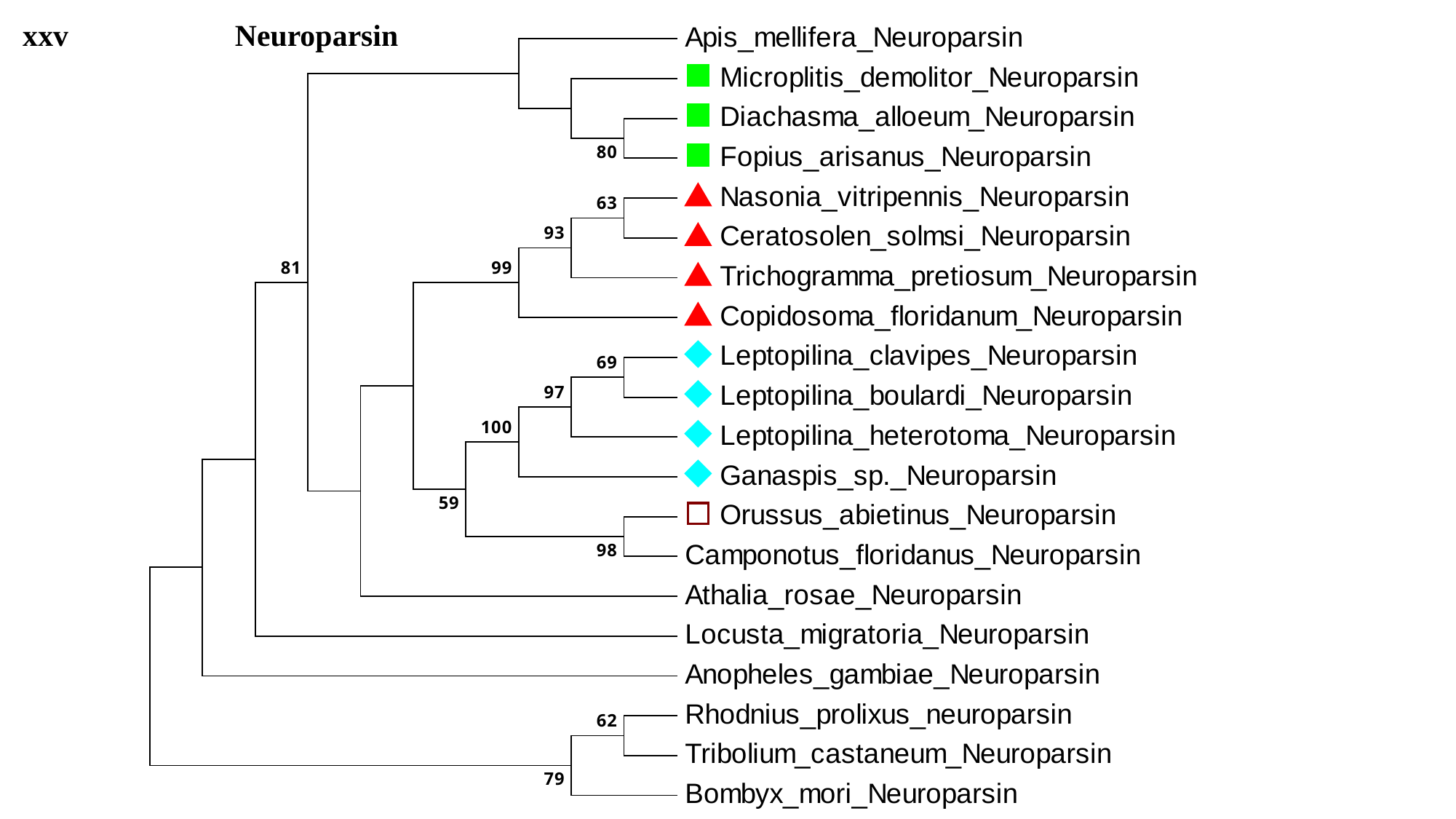

Neuroparsin
xxv

## Slide 26
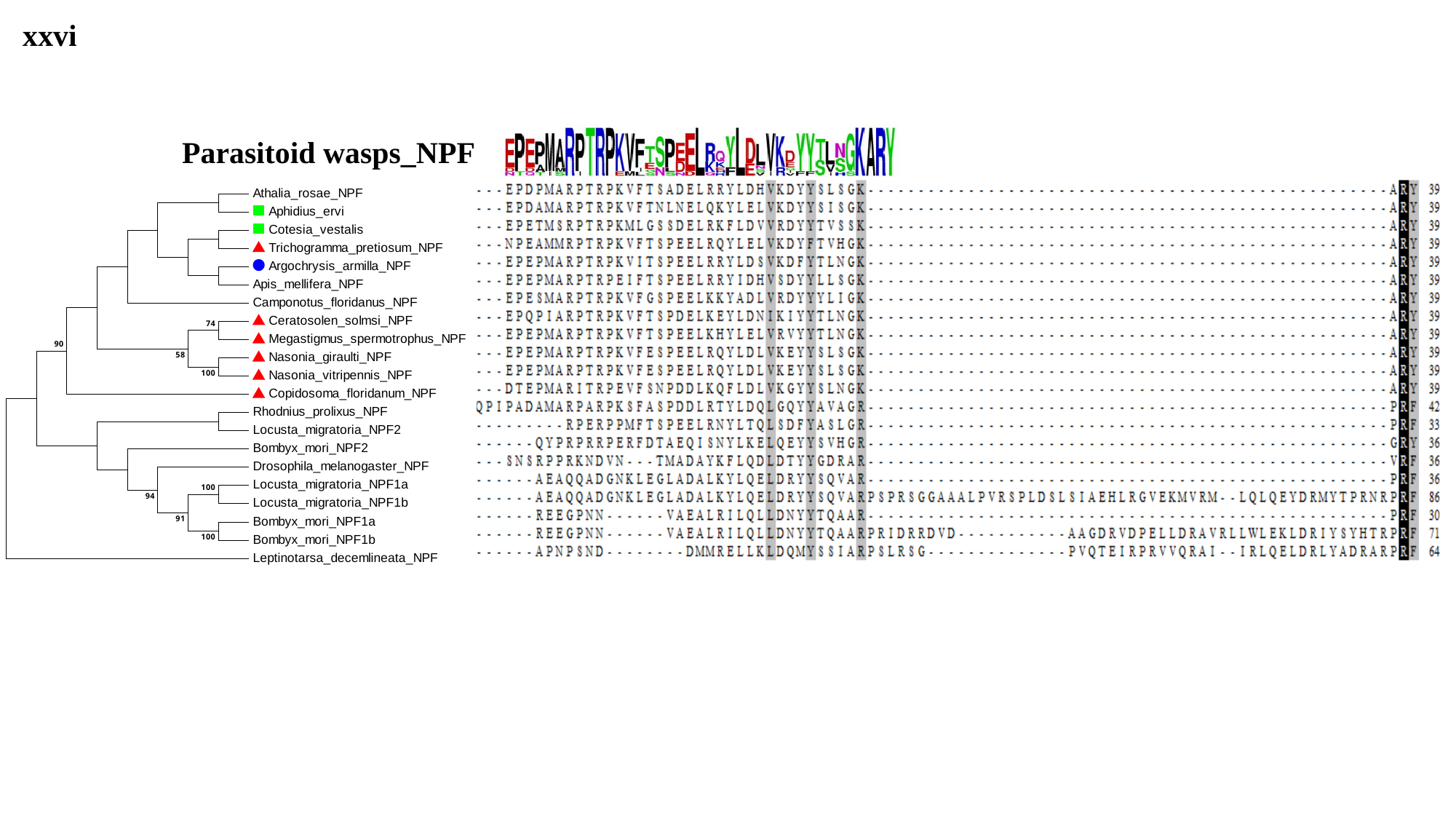

xxvi
Parasitoid wasps_NPF

## Slide 27
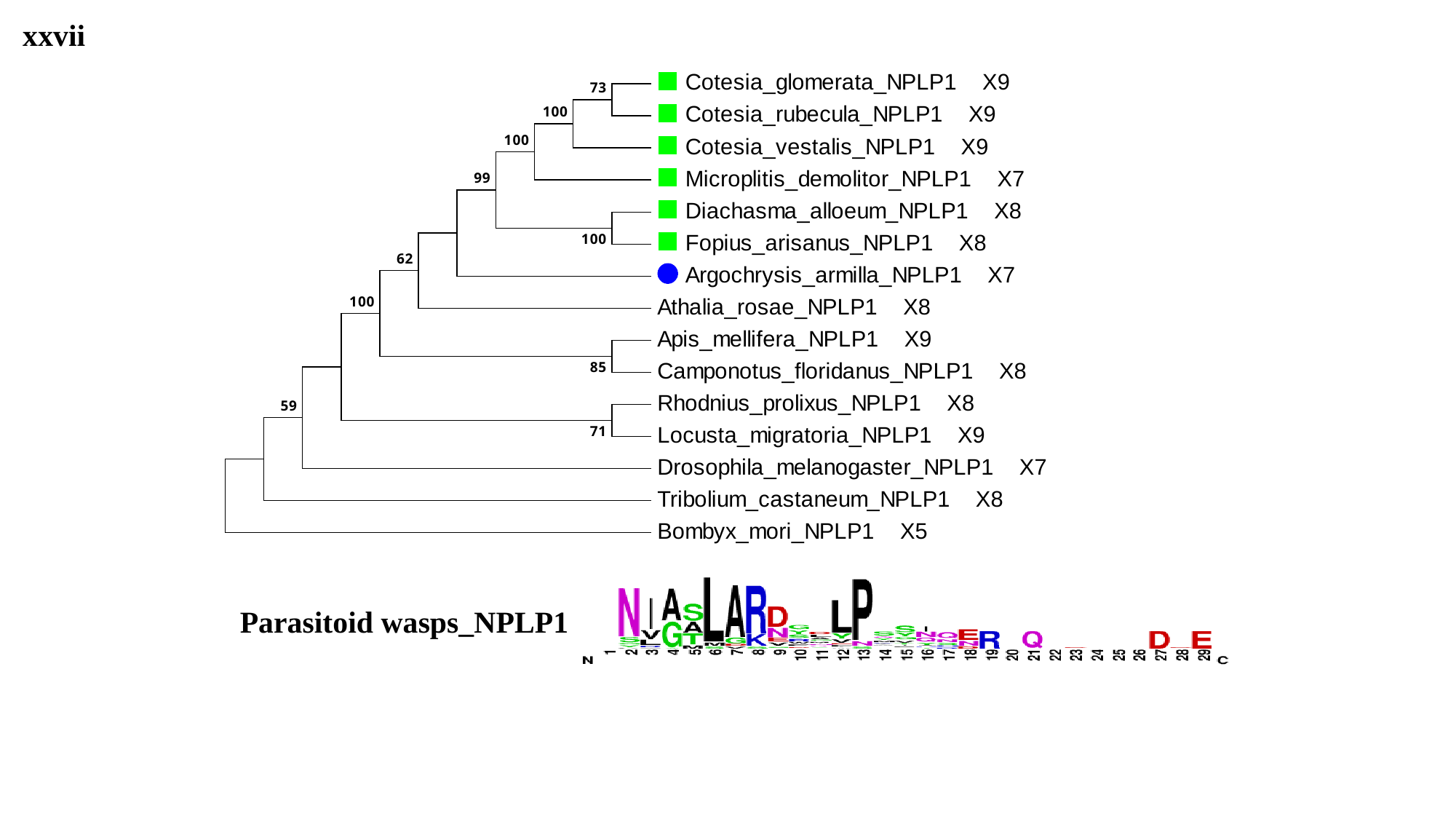

xxvii
Parasitoid wasps_NPLP1

## Slide 28
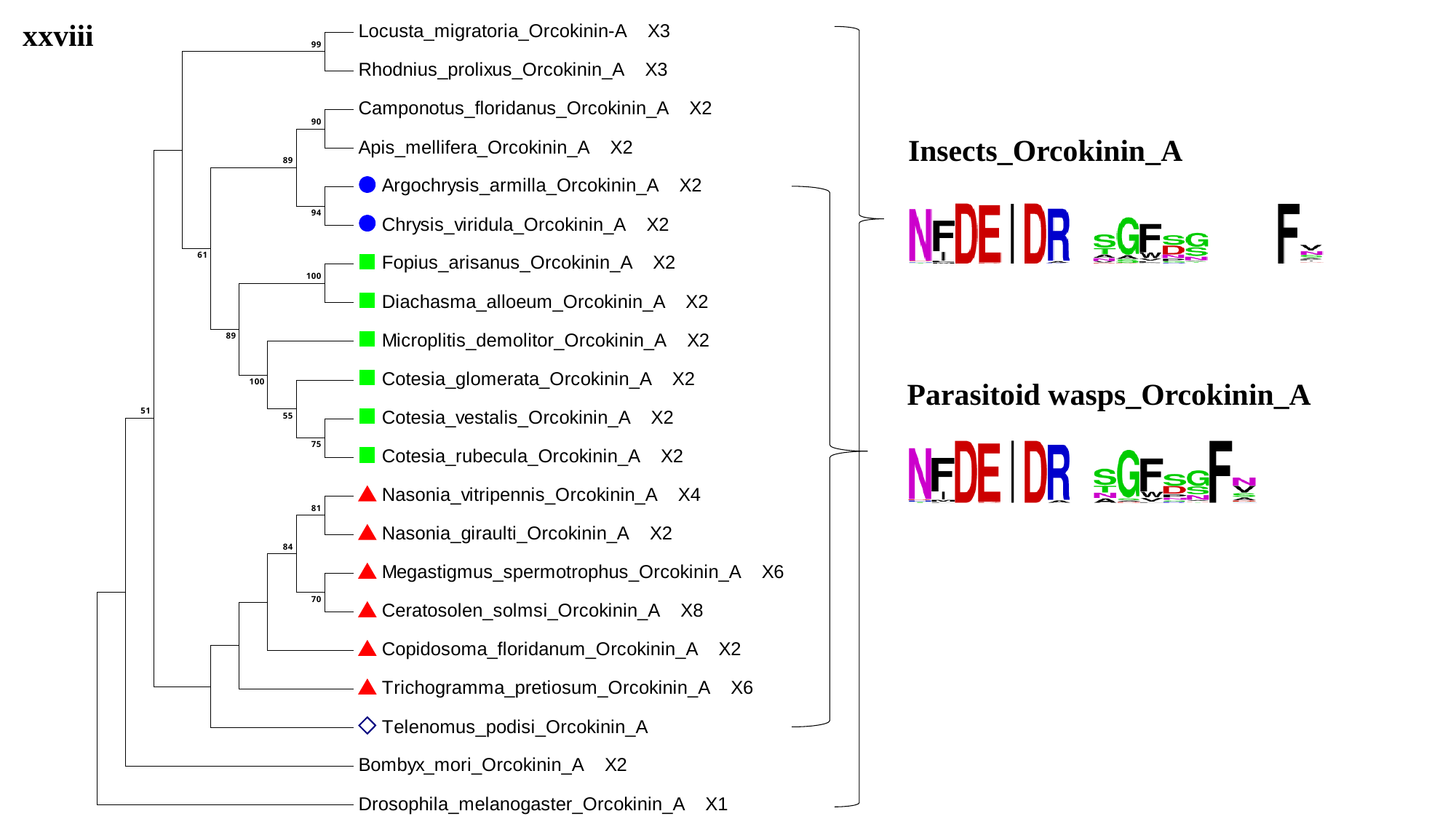

xxviii
Insects_Orcokinin_A
Parasitoid wasps_Orcokinin_A

## Slide 29
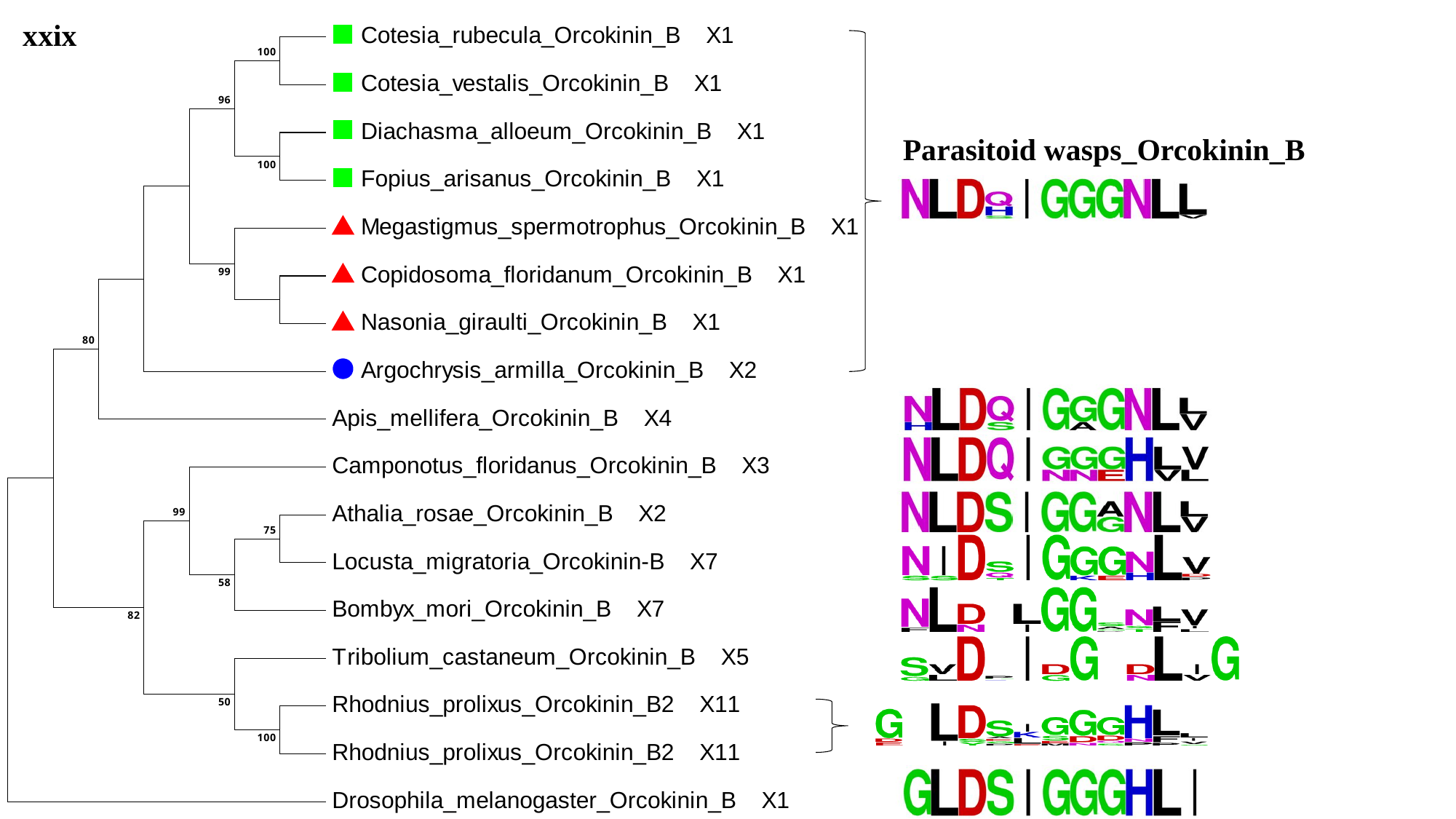

xxix
Parasitoid wasps_Orcokinin_B

## Slide 30
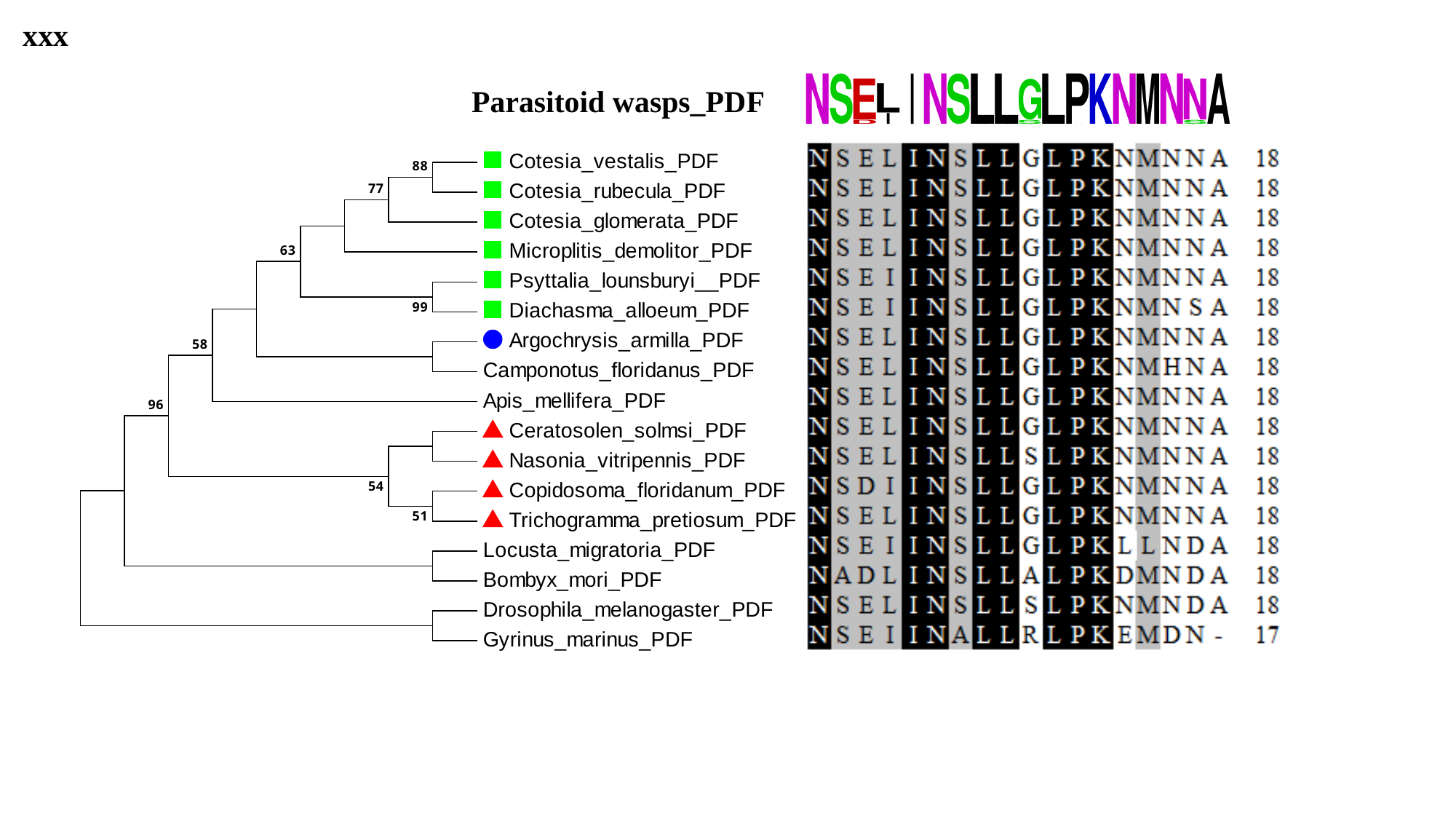

xxx
Parasitoid wasps_PDF

## Slide 31
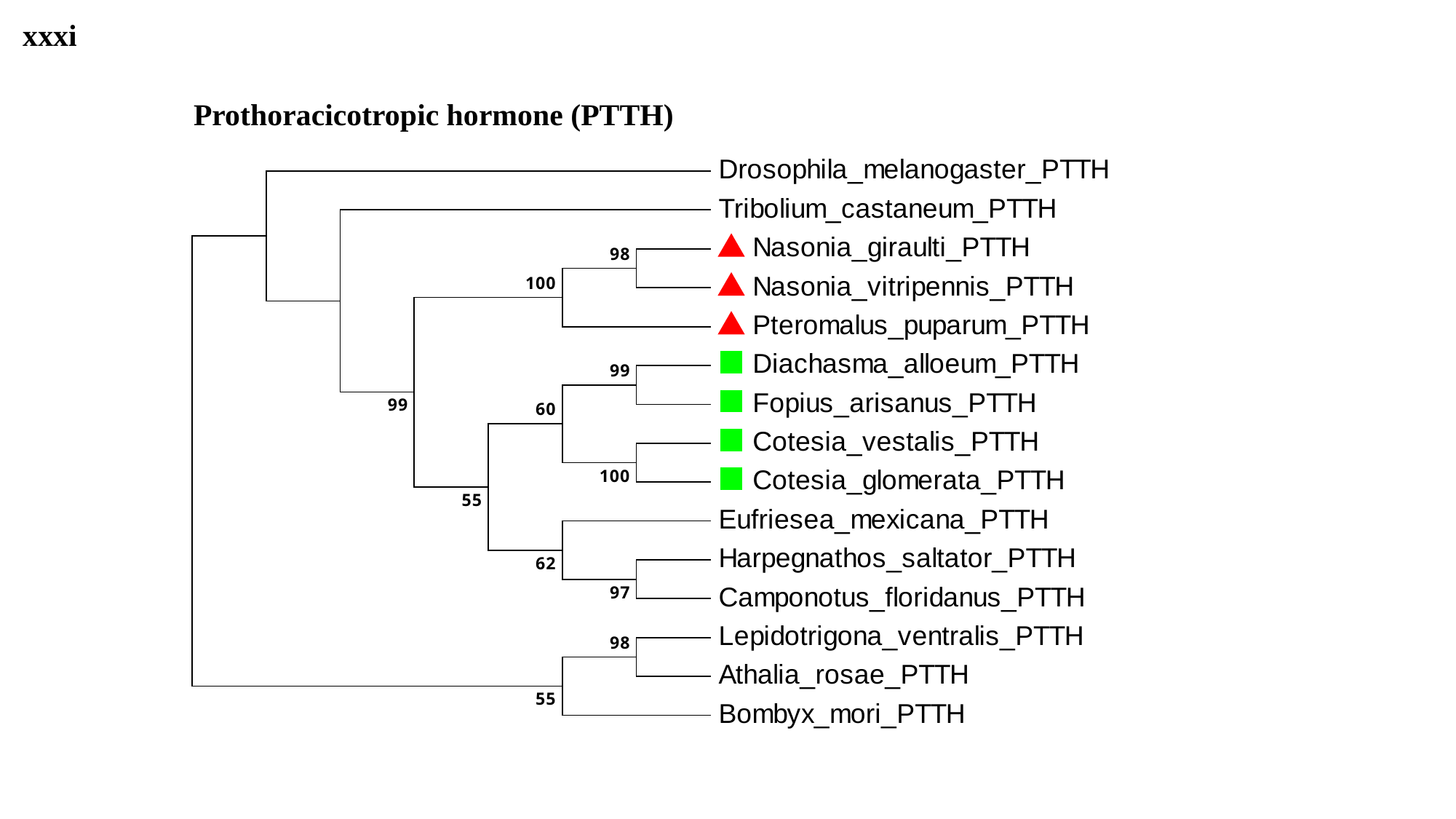

xxxi
Prothoracicotropic hormone (PTTH)

## Slide 32
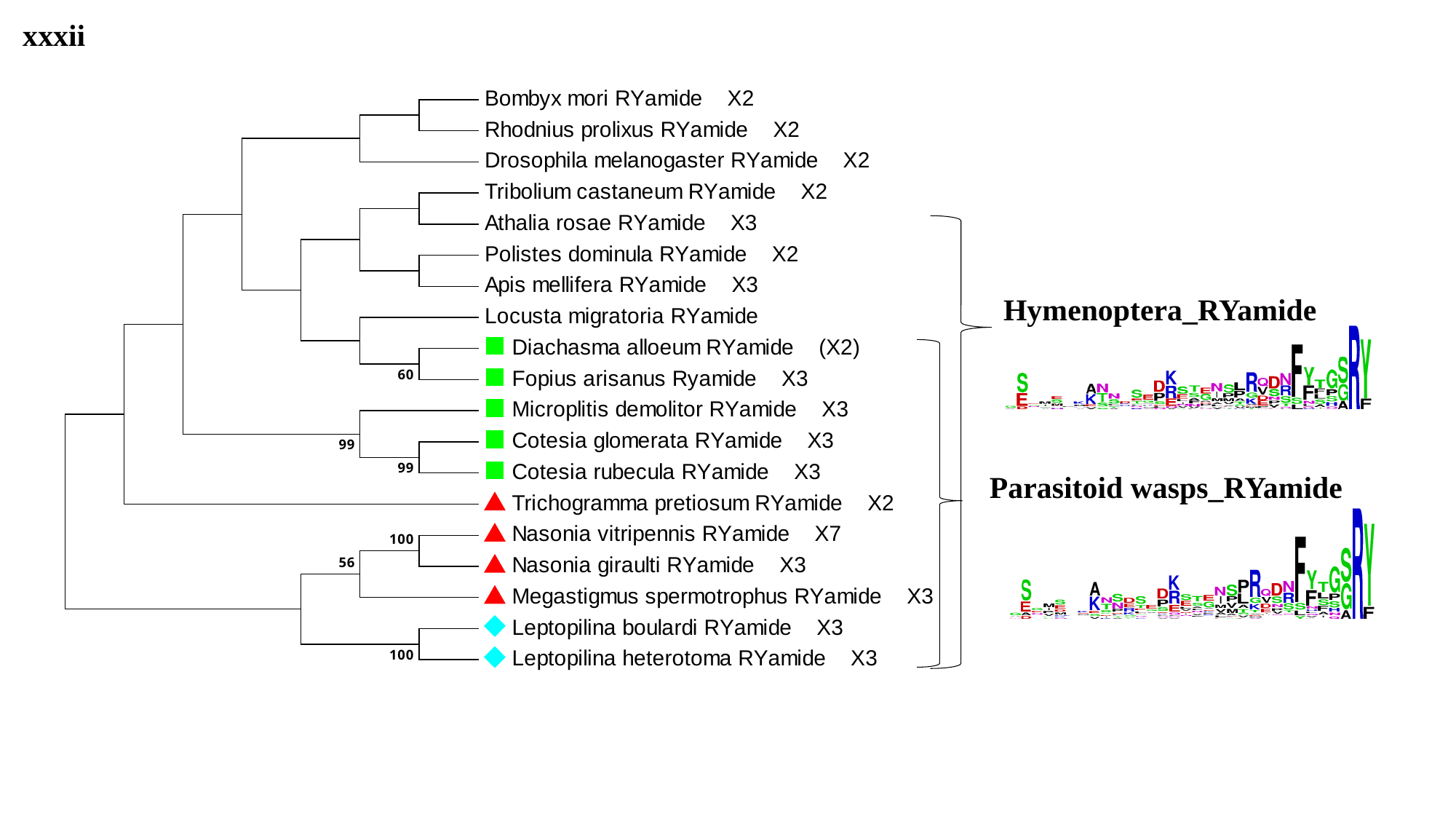

xxxii
Hymenoptera_RYamide
Parasitoid wasps_RYamide

## Slide 33
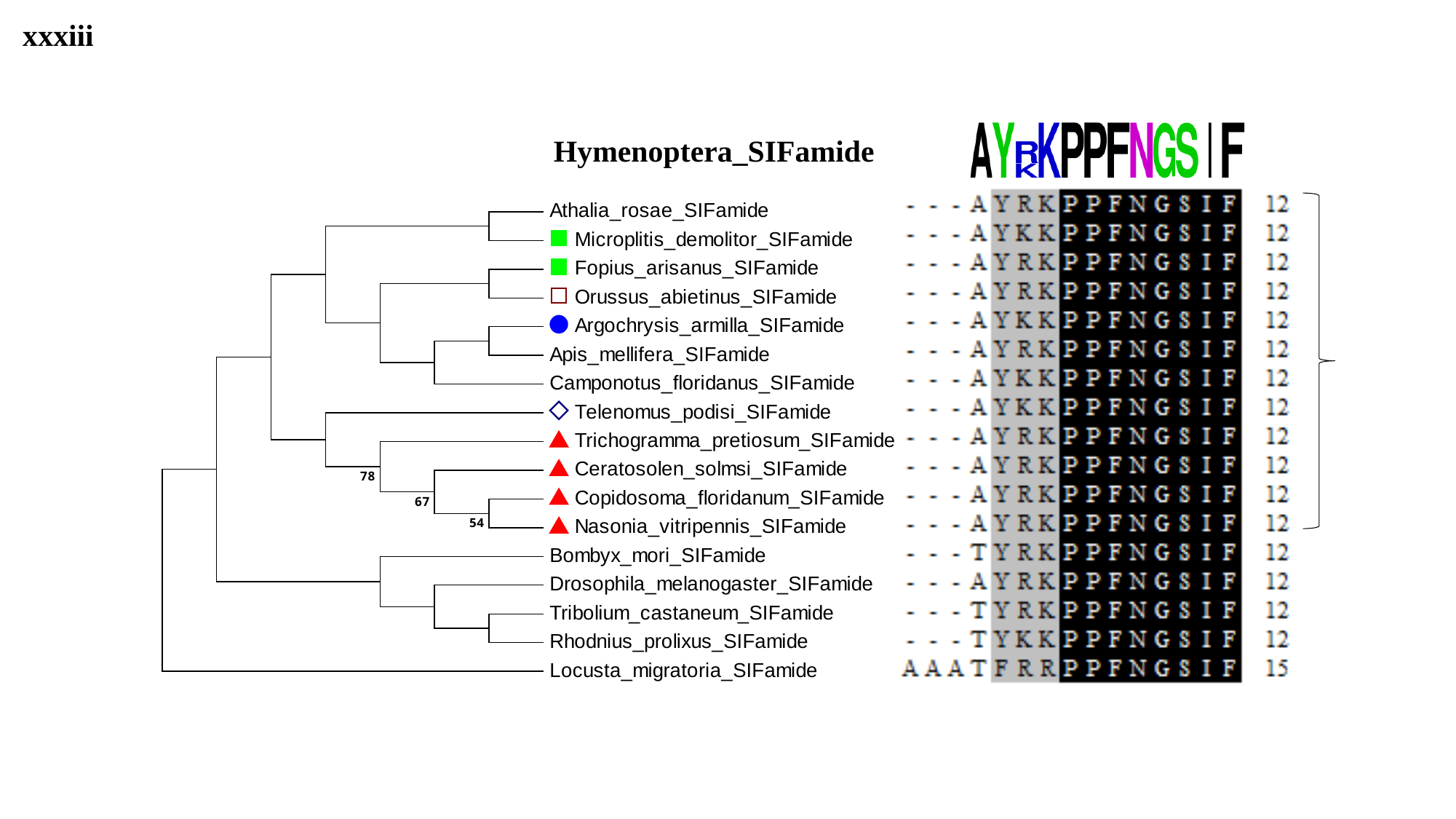

xxxiii
Hymenoptera_SIFamide

## Slide 34
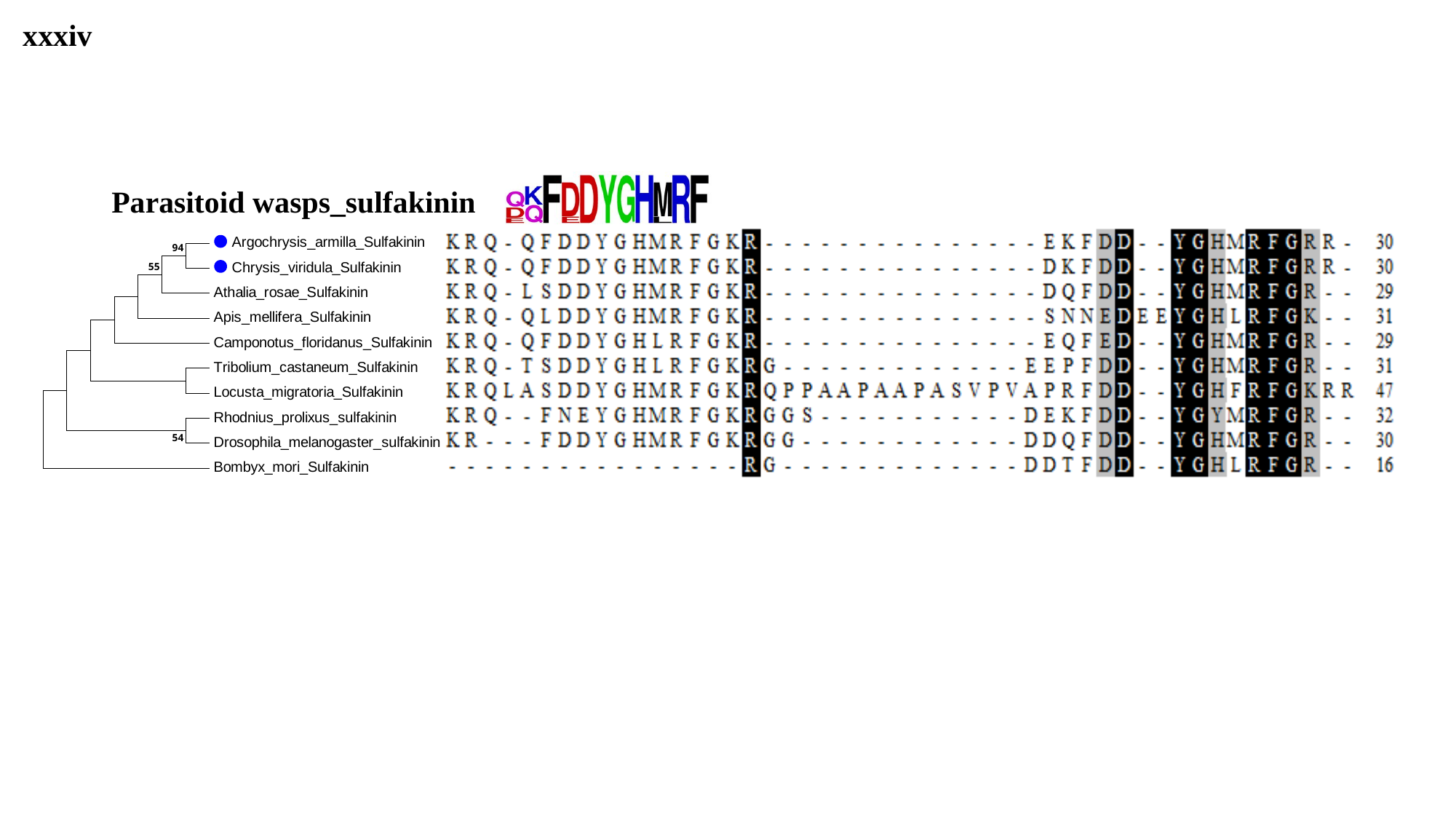

xxxiv
Parasitoid wasps_sulfakinin

## Slide 35
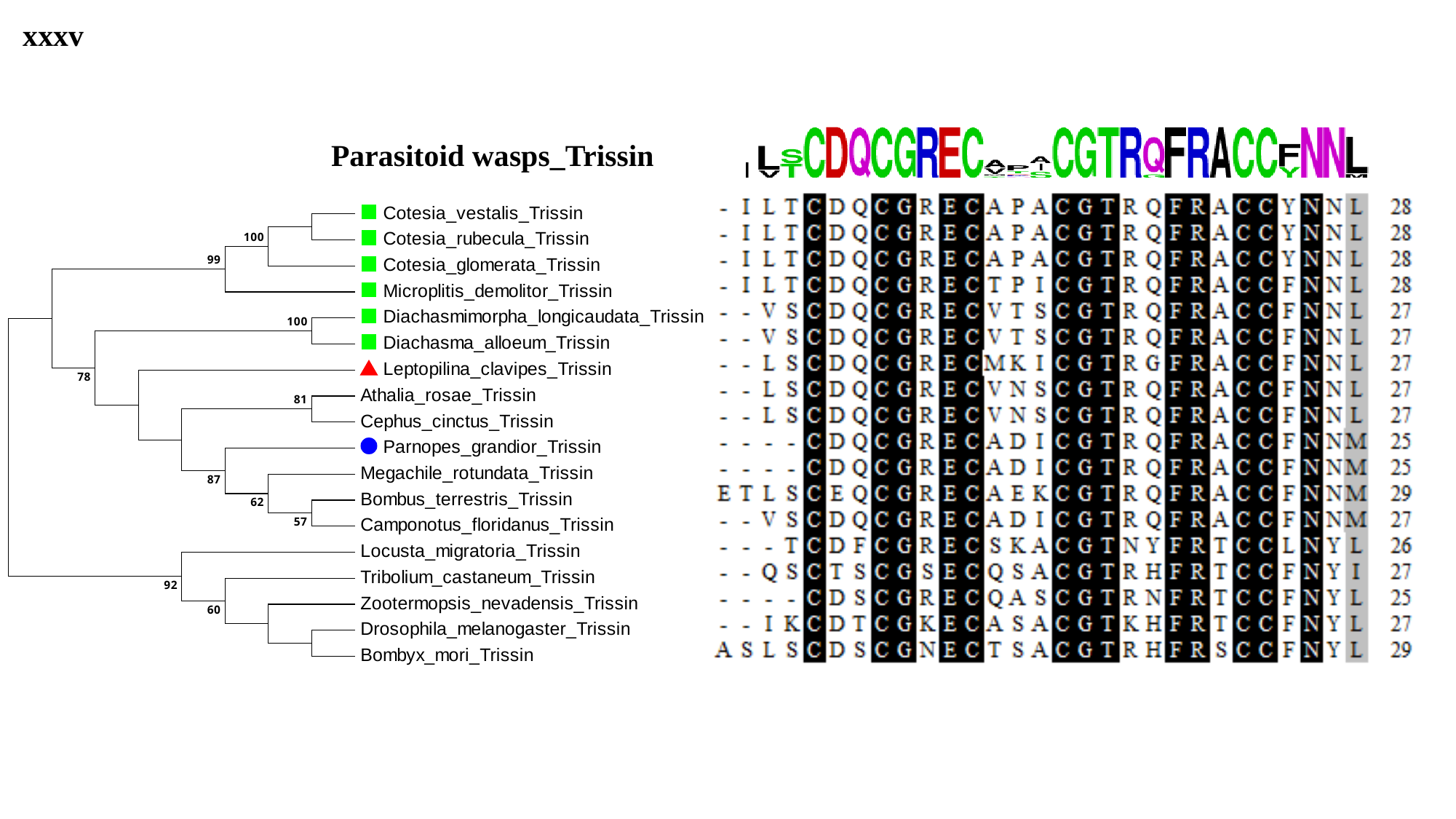

xxxv
Parasitoid wasps_Trissin
